# Supplementary material for: Defective three-dimensional covalent organic frameworks for enhanced hydrogen peroxide photosynthesis and organic transformation
Source: Nat Commun. 2026 Mar 27;17:4505. doi: 10.1038/s41467-026-71137-0 (PMC13187191; doi:10.1038/s41467-026-71137-0)
Supplement: Supplementary file 1 — Supplementary Information [file 41467_2026_71137_MOESM1_ESM.pdf]

Supplementary Information for

Defective Three-dimensional Covalent Organic Frameworks for Enhanced Hydrogen Peroxide Photosynthesis and Organic Transformation

Tengteng Dong,[1] Xiaohui Xu,[2,] Li Chen,[1] Jiani Yang,[1] Mengchao Guo,[1] Mi Zhou,[1] Min Xu,[3] Weichao Xue,[3] Xiancheng Ren,[1] Shuang Li,[1,*] and Chong Cheng[1,4,*]*

[1] College of Polymer Science and Engineering, State Key Laboratory of Advanced Polymer Materials, Sichuan University, Chengdu, 610065, China

[2] Department of Medical Ultrasound, Frontiers Science Center for Disease-Related Molecular Network, West China Hospital, Sichuan University, Chengdu, 610041, China

[3] College of Chemistry, Sichuan University, Chengdu, 610065, China

[4] Department of Endodontics, State Key Laboratory of Oral Diseases, National Center for Stomatology, West China Hospital of Stomatology, Sichuan University, Chengdu, 610041, China

* Corresponding author.

E-mail: (X. Xu) xiaohuixu@scu.edu.cn; (S. Li) shuang.li@scu.edu.cn; (C. Cheng) chong.cheng@scu.edu.cn

Supplementary Figures

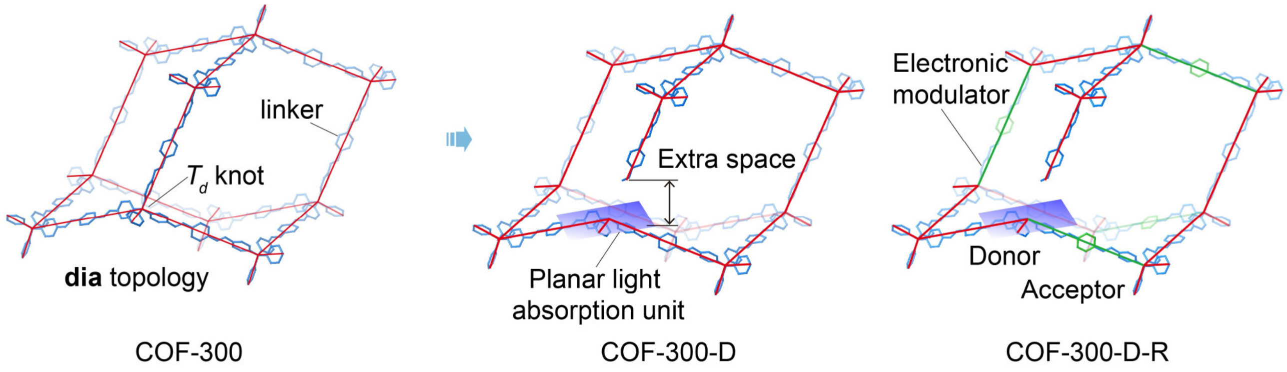

	COF-300	COF-300-D	COF-300-D-R
Structural stability	good	good	good
Light absorption capability	disfavoured	enhanced	enhanced
Charge seperation efficiency	less efficient	less efficient	efficient

Supplementary Fig. 1 Structural diagrams for three COFs and comparisons of their various characteristics.

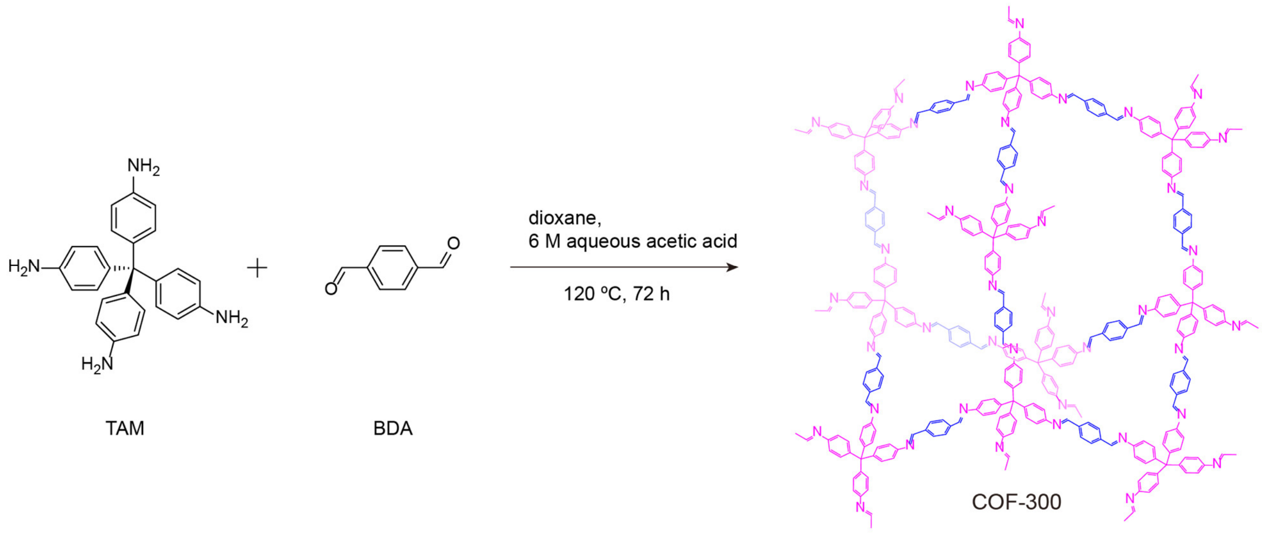

Supplementary Fig. 2 Schematic diagram for the synthesis of COF-300.

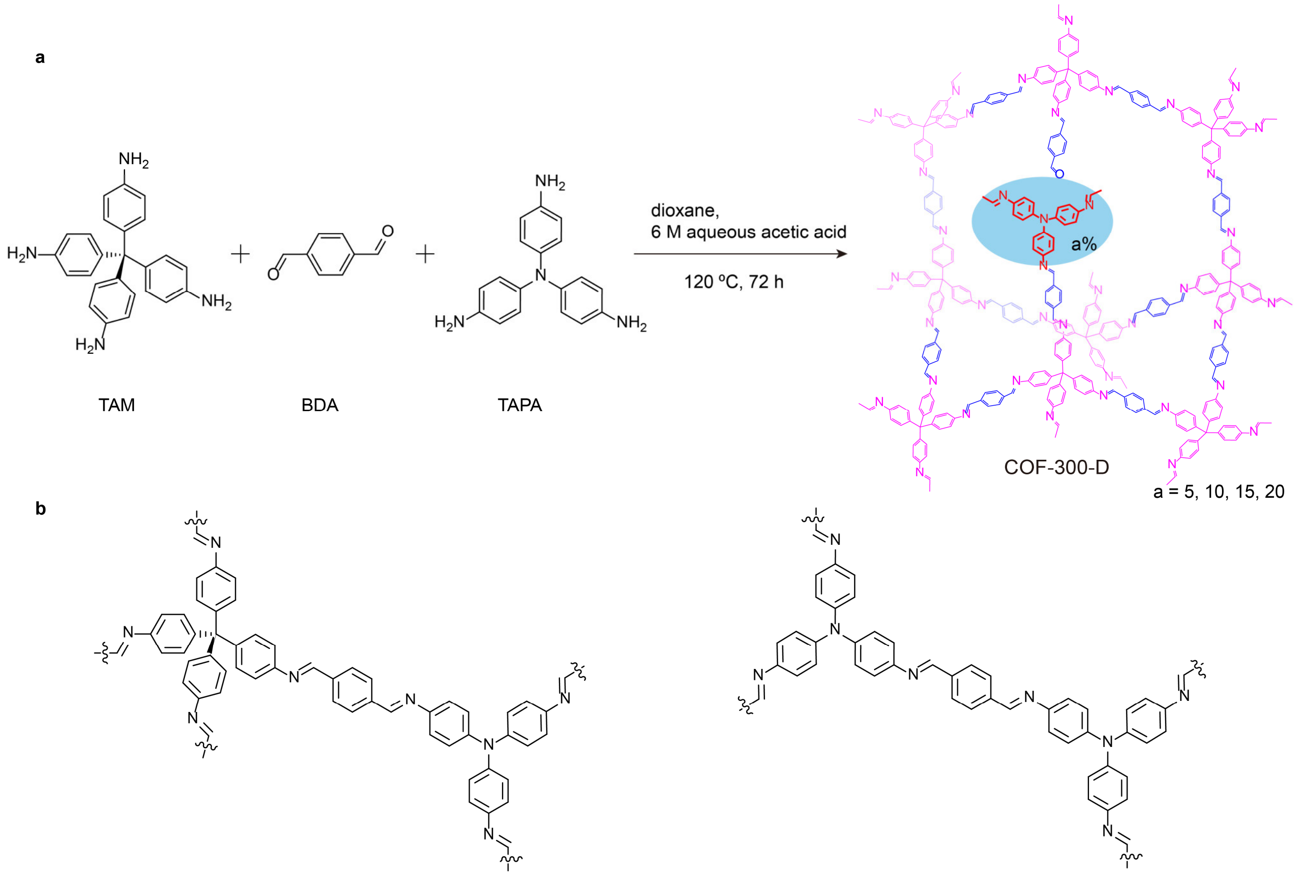

Supplementary Fig. 3 Schematic diagram of the synthesis and possible conformations of COF-300-D. a, Schematic diagram for the synthesis of the series of COFs with different contents of TAPA (COF-300-D) (D = 5%, 10%, 15%, 20%). **b,** Schematic diagram of possible conformations of COF-300-D series. The doping of mixed ligands follows a statistical distribution. With the increase in TAPA content, the probability of both ends of the BDA connector being connected to the plane TAPA nodes increases significantly, which may lead to a decrease in the long-range order of the COF-300-D series with a high doping ratio.

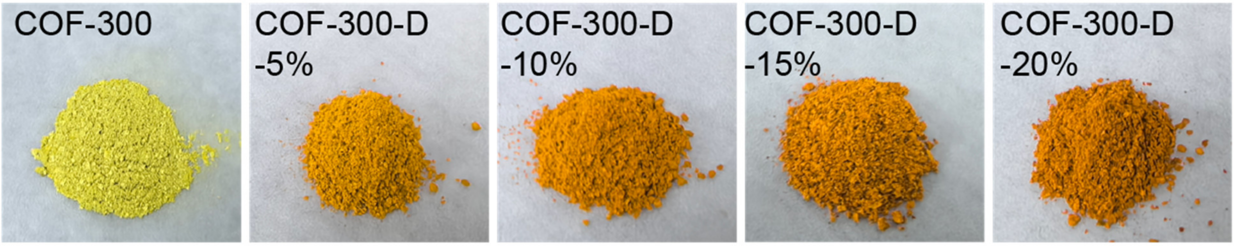

Supplementary Fig. 4 Optical pictures of COF-300, COF-300-D-5%, -10%, -15%, and -20%, respectively.

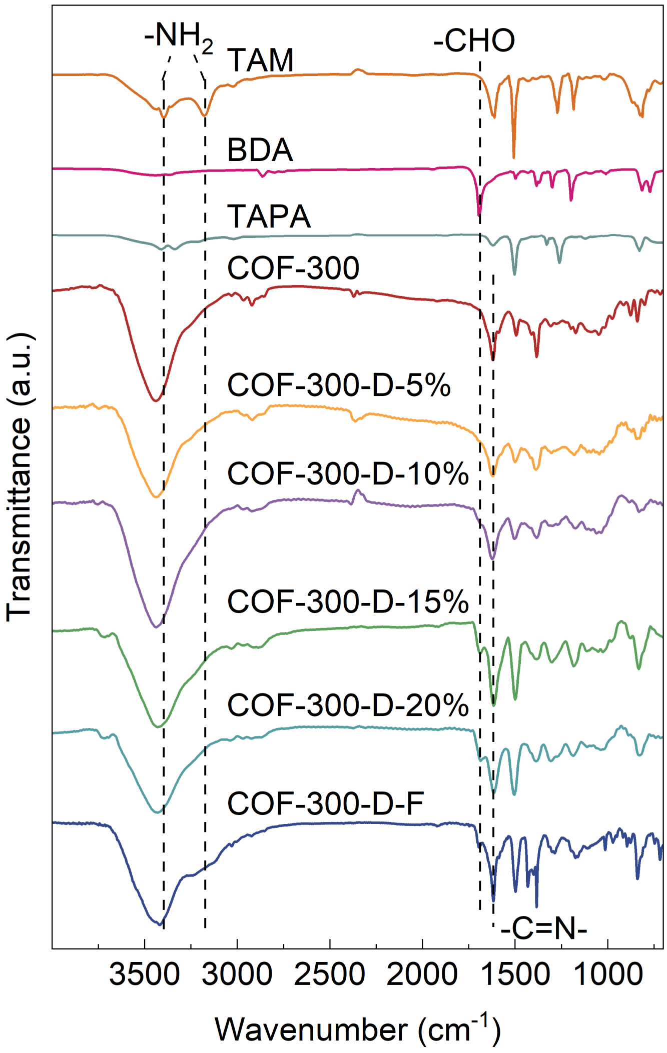

Supplementary Fig. 5 FT-IR spectra of the starting materials (tetrakis(4-aminophenyl)methane (TAM), terephthalaldehyde (BDA), tris(4-aminophenyl)amine (TAPA)) used in synthesis, COF-300, COF-300-D, and the optimized 3D COFs with planar ligands induced defects and fluorine functional groups (COF-300-D-F). The new emergence of an imine C=N stretch vibration of COFs was observed at ~1624 cm^{-1}. IR peaks at ~3399 cm^{-1} for TAM (assigned to the N-H stretching mode of the amino group) and 1697 cm^{-1} for BDA (assigned to the C=O stretching mode of aldehyde) disappeared in that of COF-300, which indicated the complete reaction of raw materials. With the increase of TAPA doping amount, the C=O stretching mode of aldehyde (1697 cm^{-1}) appeared, which proved that TAPA was successfully introduced.

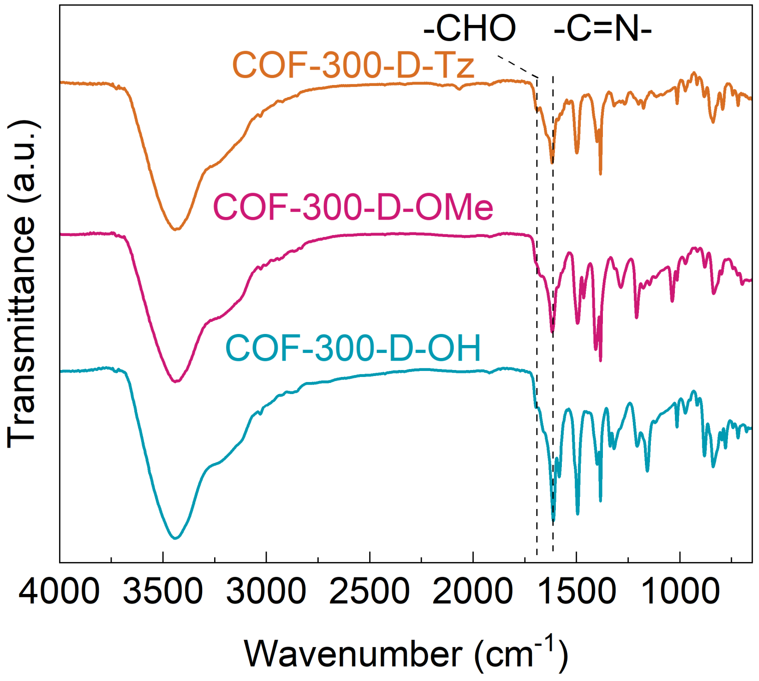

Supplementary Fig. 6 FT-IR spectra of the electronically tuned COF-300-D (COF-300-D-R, R, -Tz, -OMe, or -OH). The two dashed lines, from left to right, represent the vibration bands of C=O and C=N, respectively.

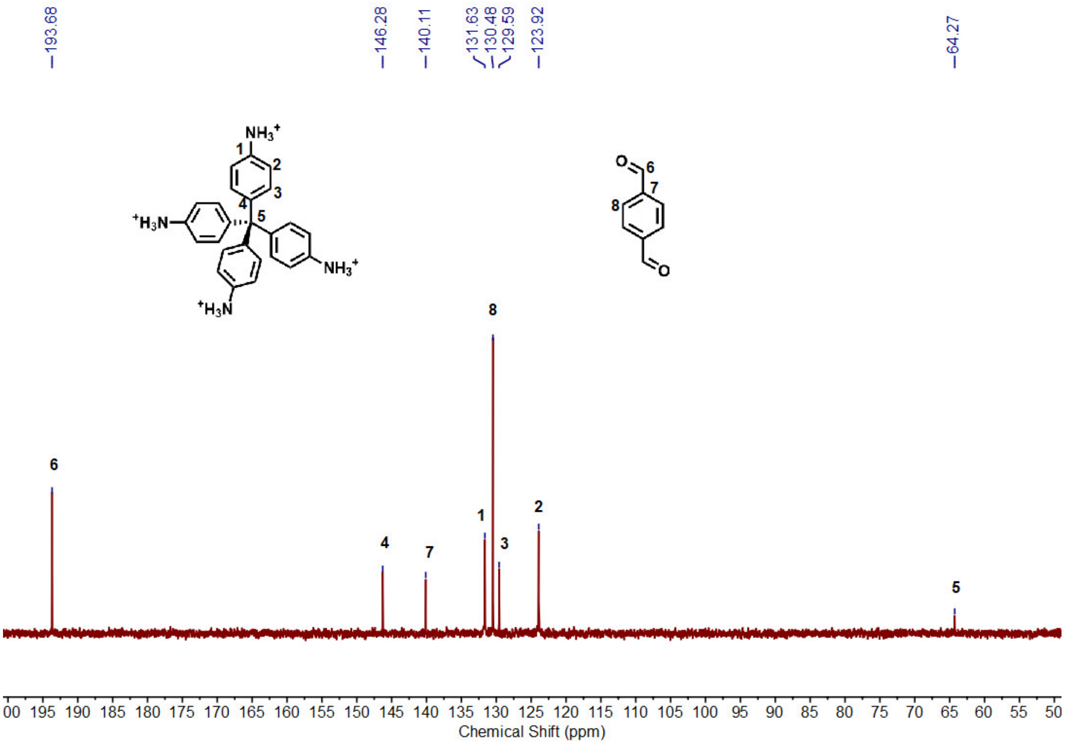

Supplementary Fig. 7 Digestion ^{13}C NMR spectrum of the activated COF-300.

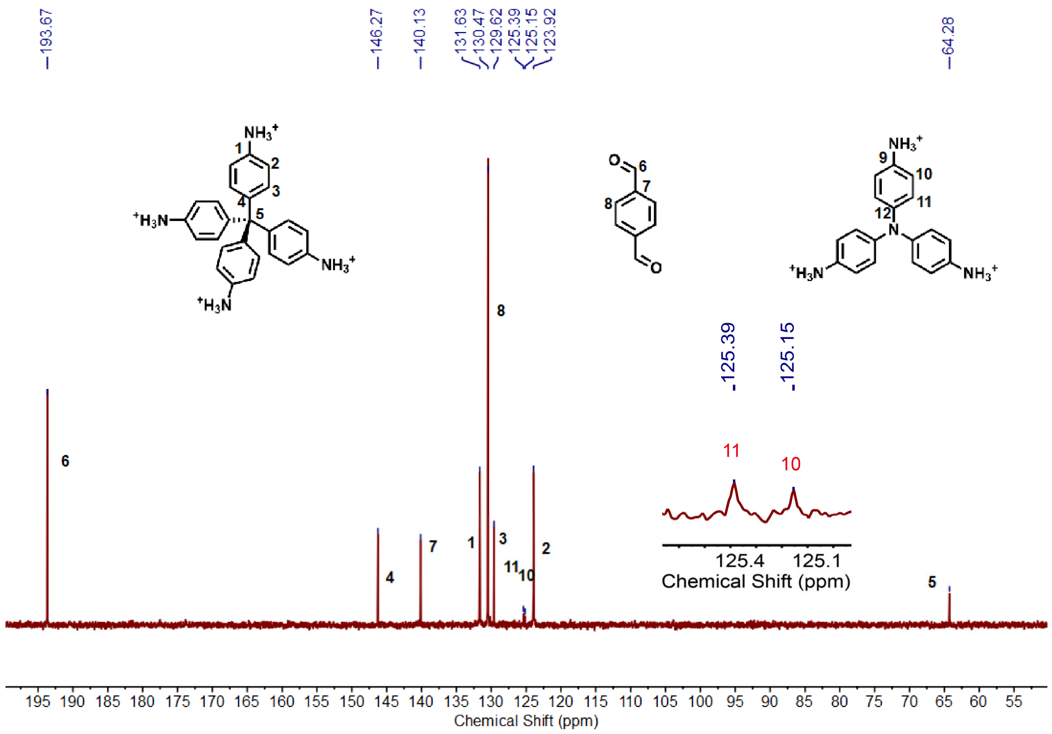

Supplementary Fig. 8 Digestion ^{13}C NMR spectrum of the activated COF-300-D-5%.

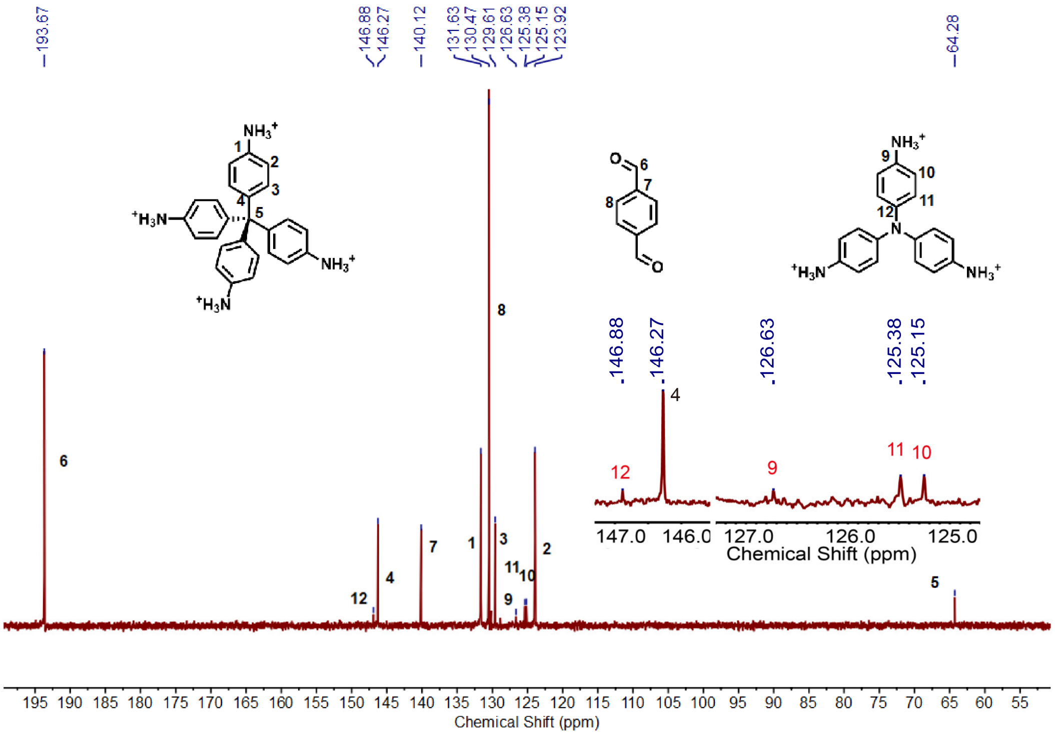

Supplementary Fig. 9 Digestion ^{13}C NMR spectrum of the activated COF-300-D-10%.

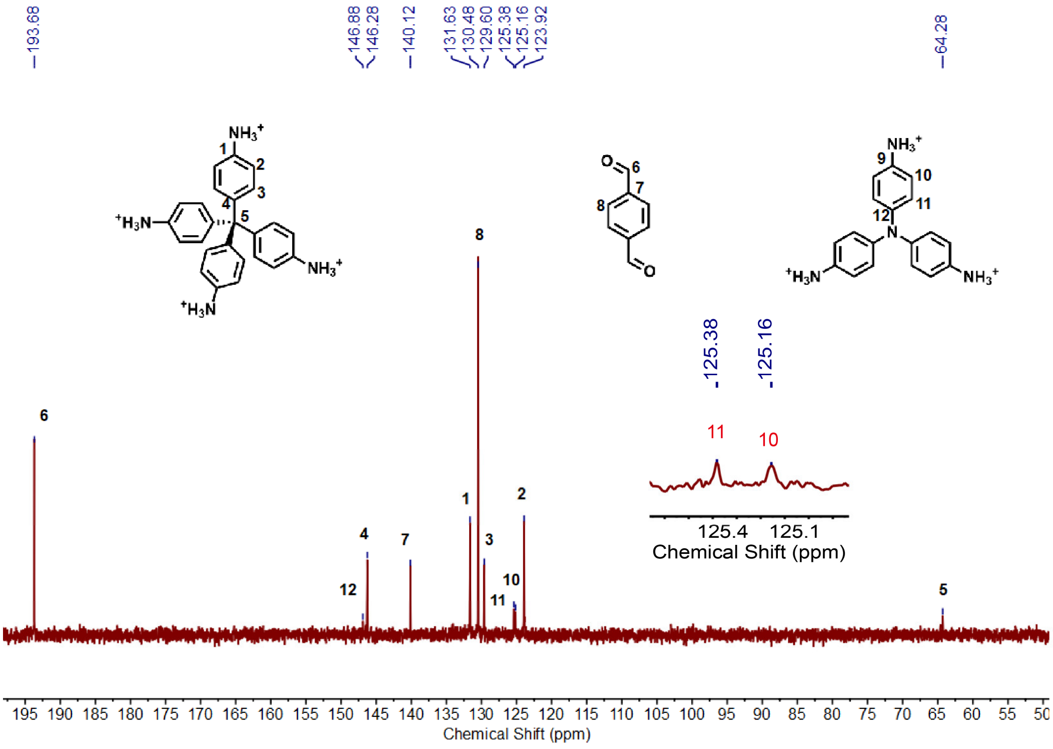

Supplementary Fig. 10 Digestion ^{13}C NMR spectrum of the activated COF-300-D-15%.

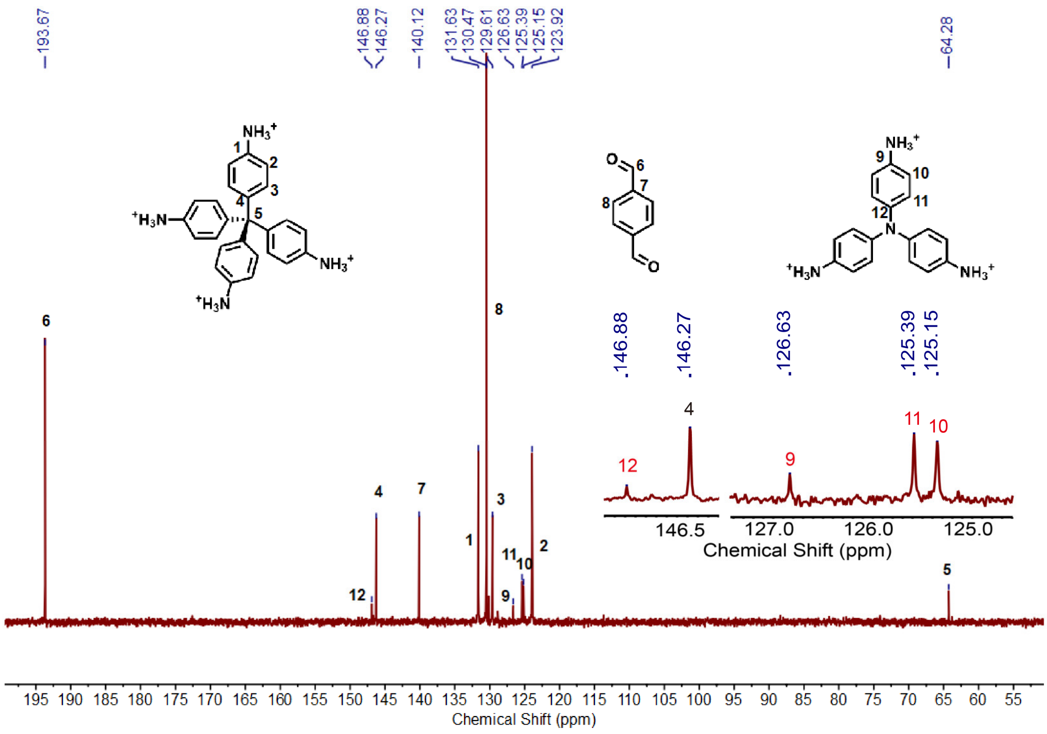

Supplementary Fig. 11 Digestion ^{13}C NMR spectrum of the activated COF-300-D-20%.

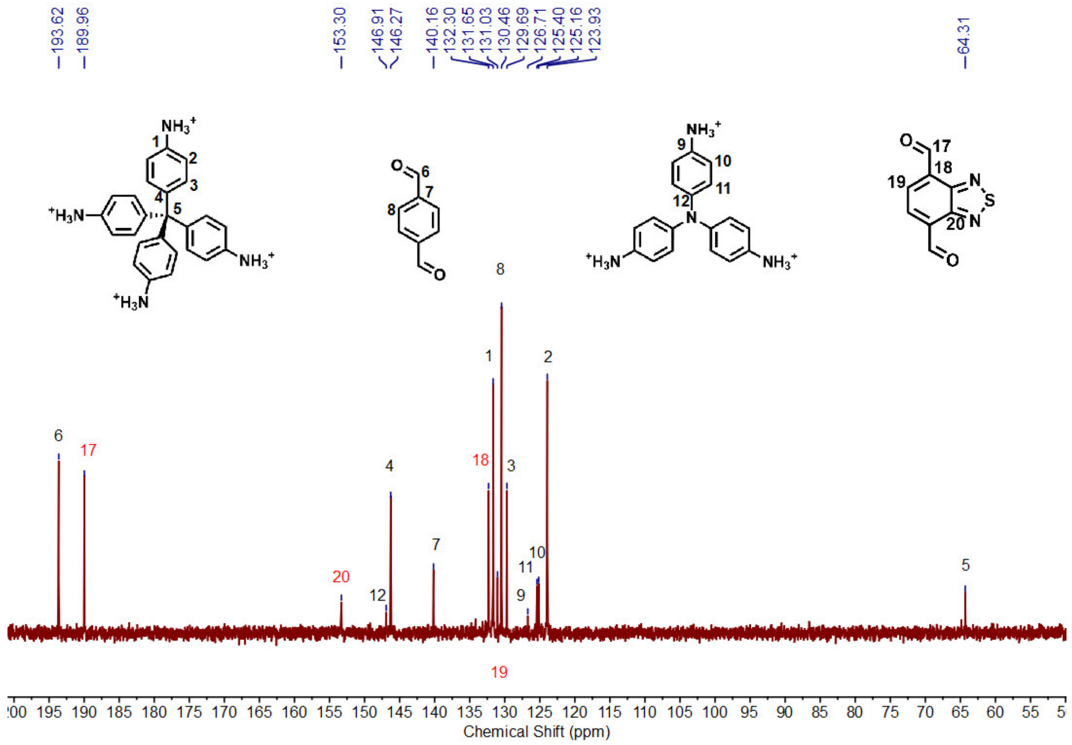

Supplementary Fig. 12 Digestion ^{13}C NMR spectrum of the activated COF-300-D-Tz.

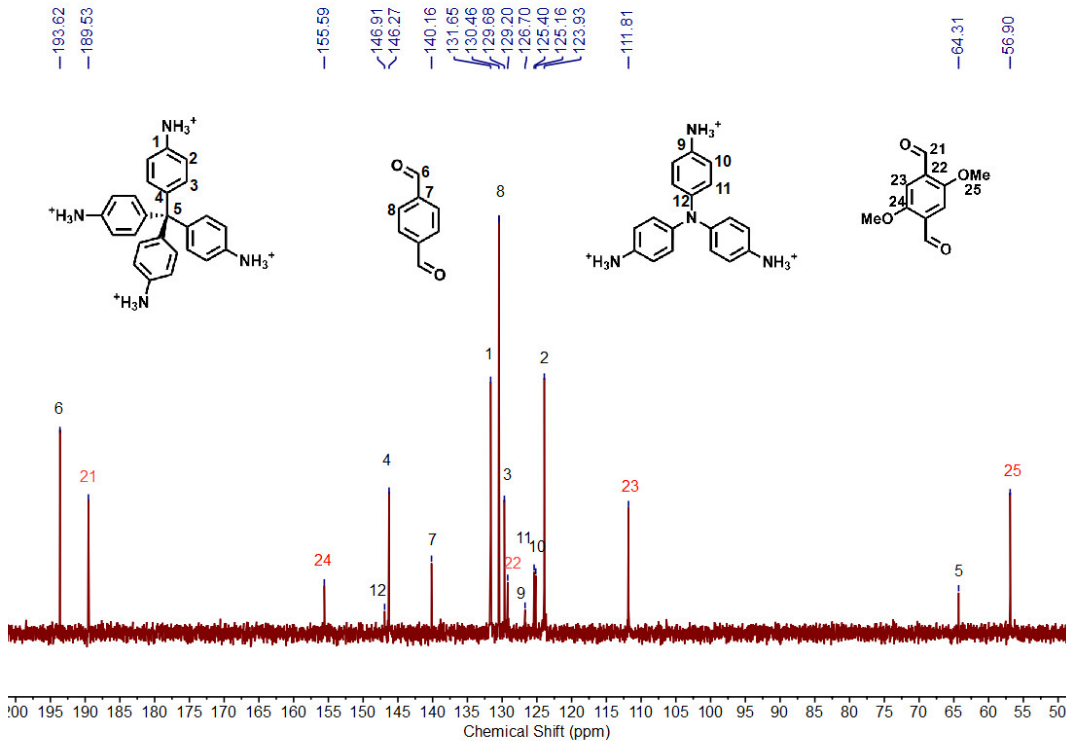

Supplementary Fig. 13 Digestion ^{13}C NMR spectrum of the activated COF-300-D-OMe.

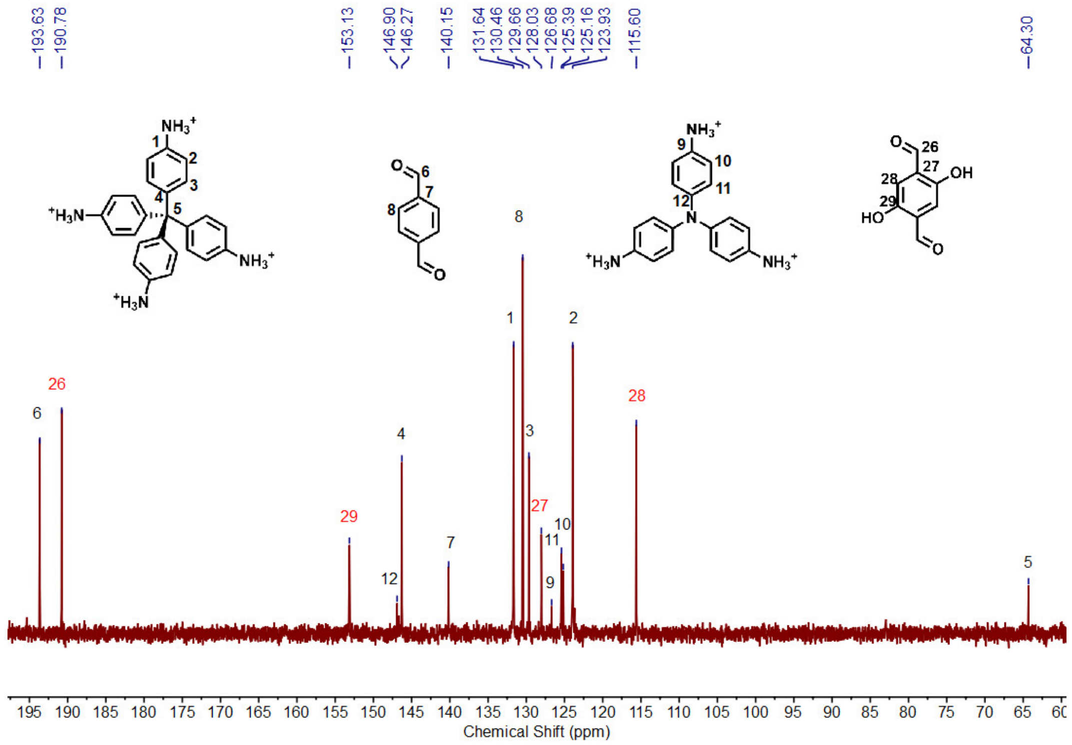

Supplementary Fig. 14 Digestion ^{13}C NMR spectrum of the activated COF-300-D-OH.

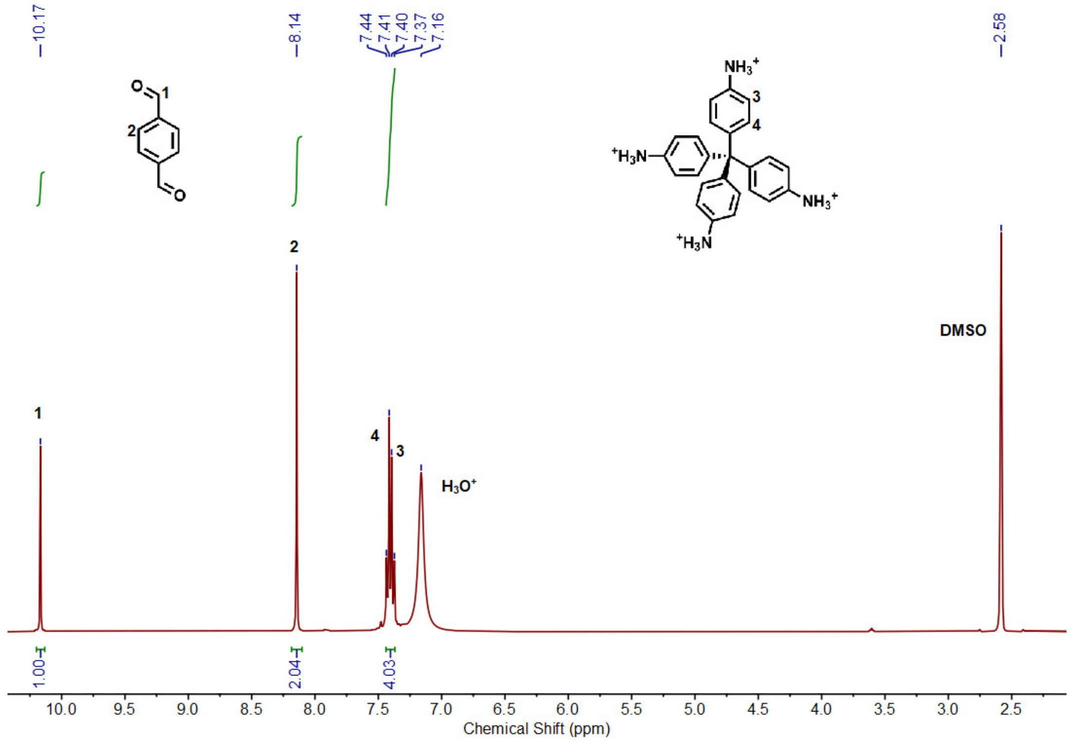

Supplementary Fig. 15 Digestion ^{1}H NMR spectrum of the activated COF-300.

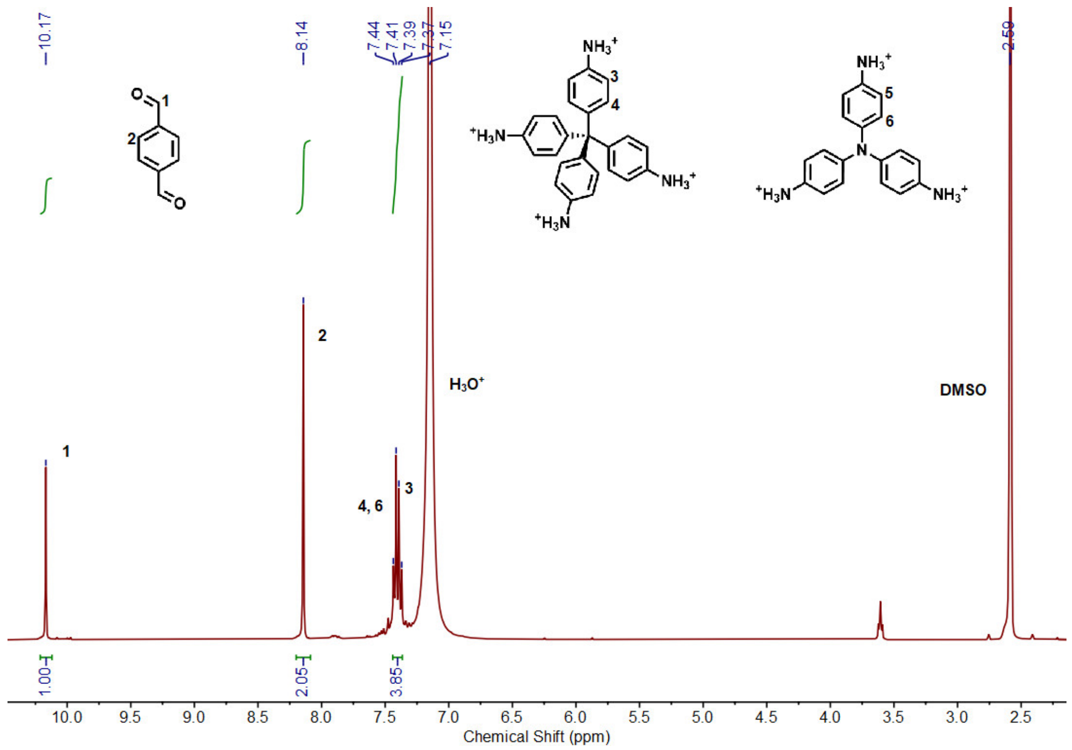

Supplementary Fig. 16 Digestion ^{1}H NMR spectrum of the activated COF-300-D-5%.

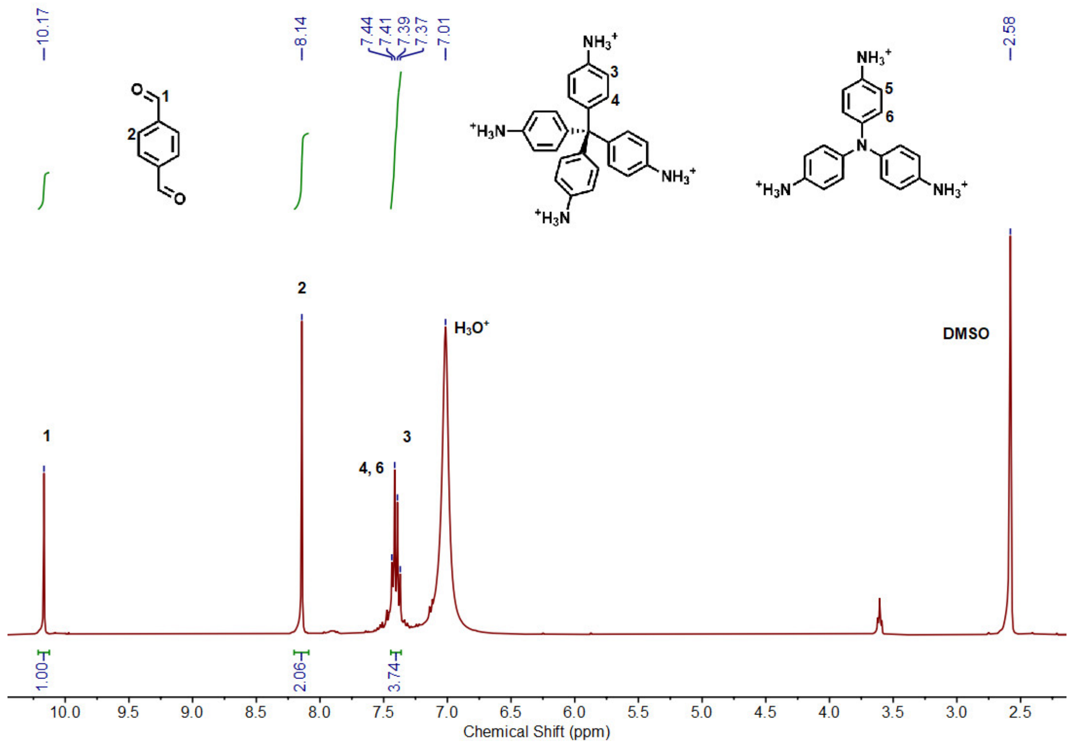

Supplementary Fig. 17 Digestion ^{1}H NMR spectrum of the activated COF-300-D-10%.

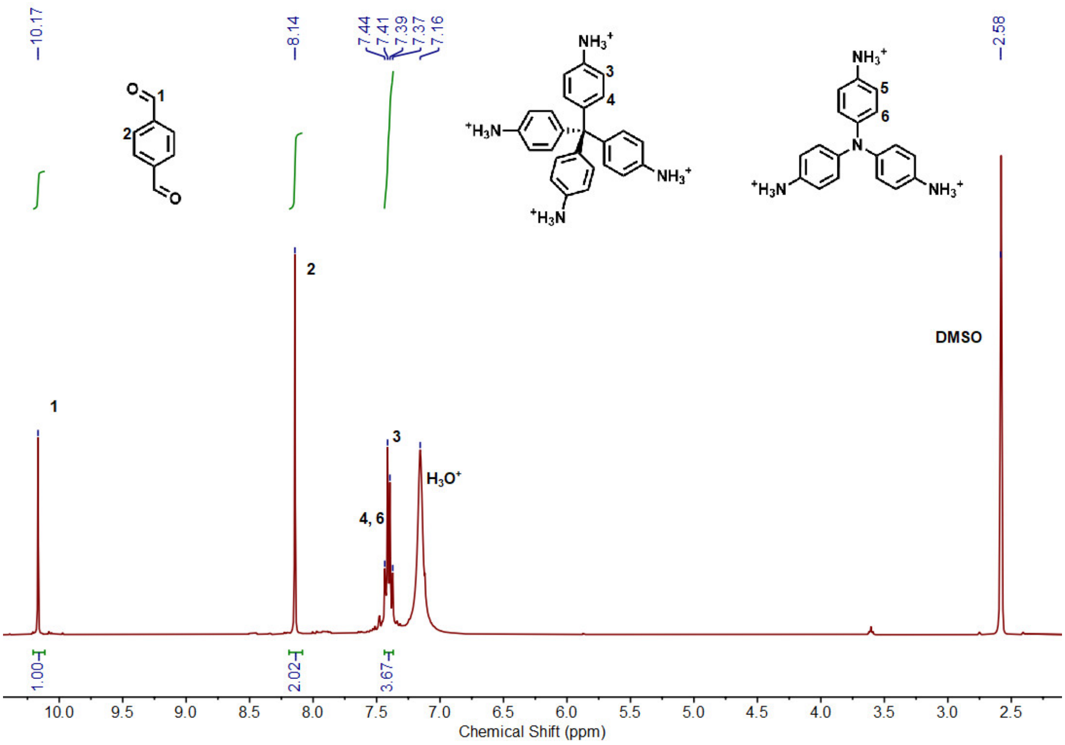

Supplementary Fig. 18 Digestion ^{1}H NMR spectrum of the activated COF-300-D-15%.

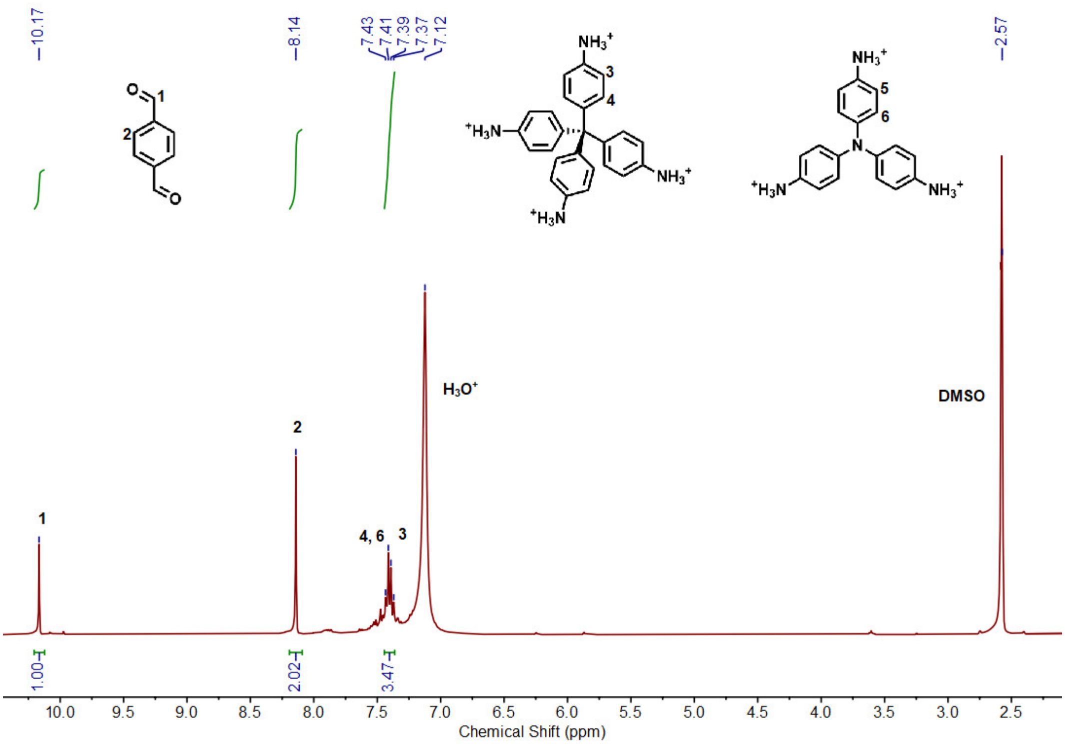

Supplementary Fig. 19 Digestion ^{1}H NMR spectrum of the activated COF-300-D-20%.

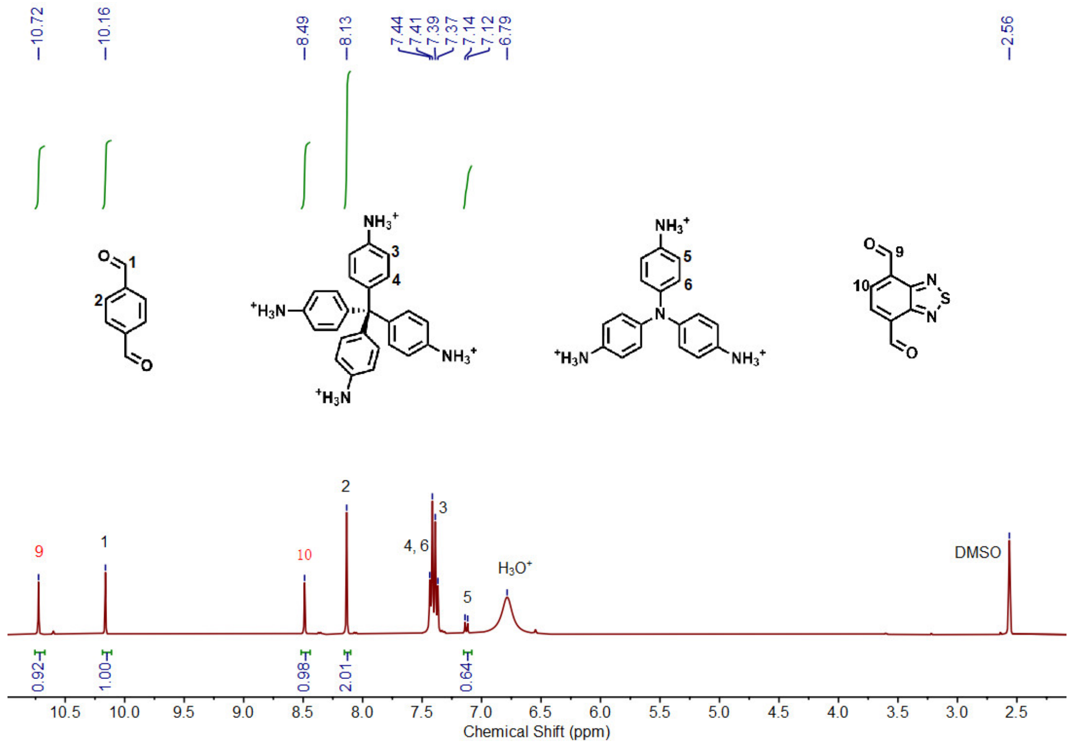

Supplementary Fig. 20 Digestion ^{1}H NMR spectrum of the activated COF-300-D-Tz.

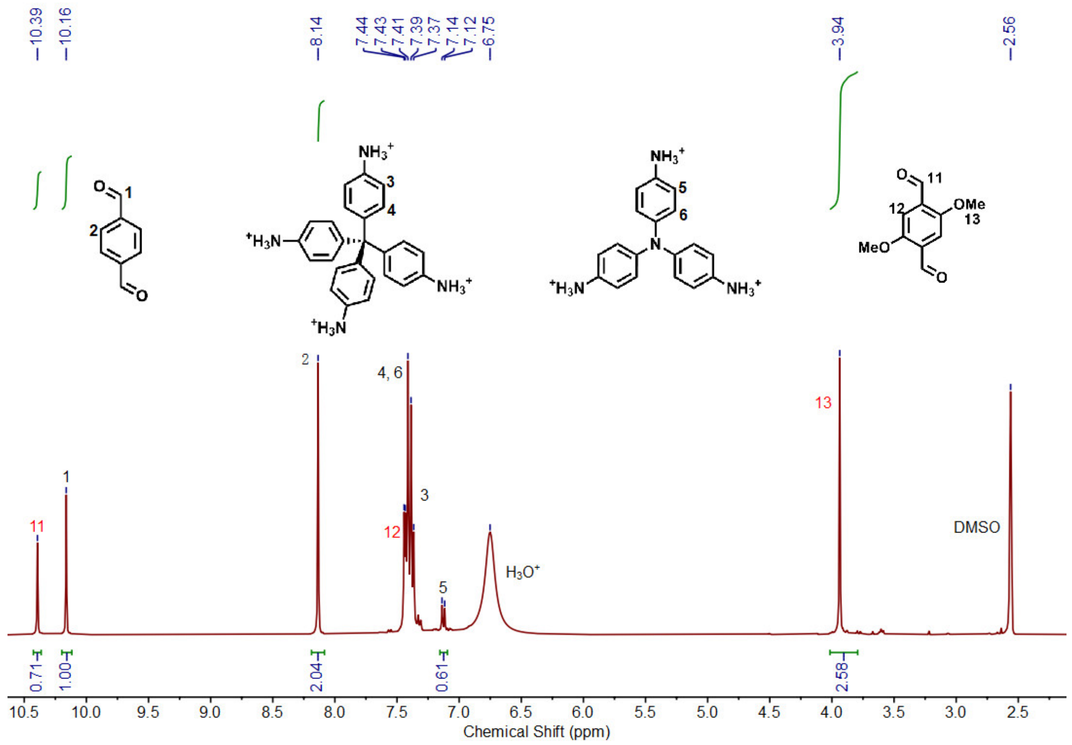

Supplementary Fig. 21 Digestion ^{1}H NMR spectrum of the activated COF-300-D-OMe.

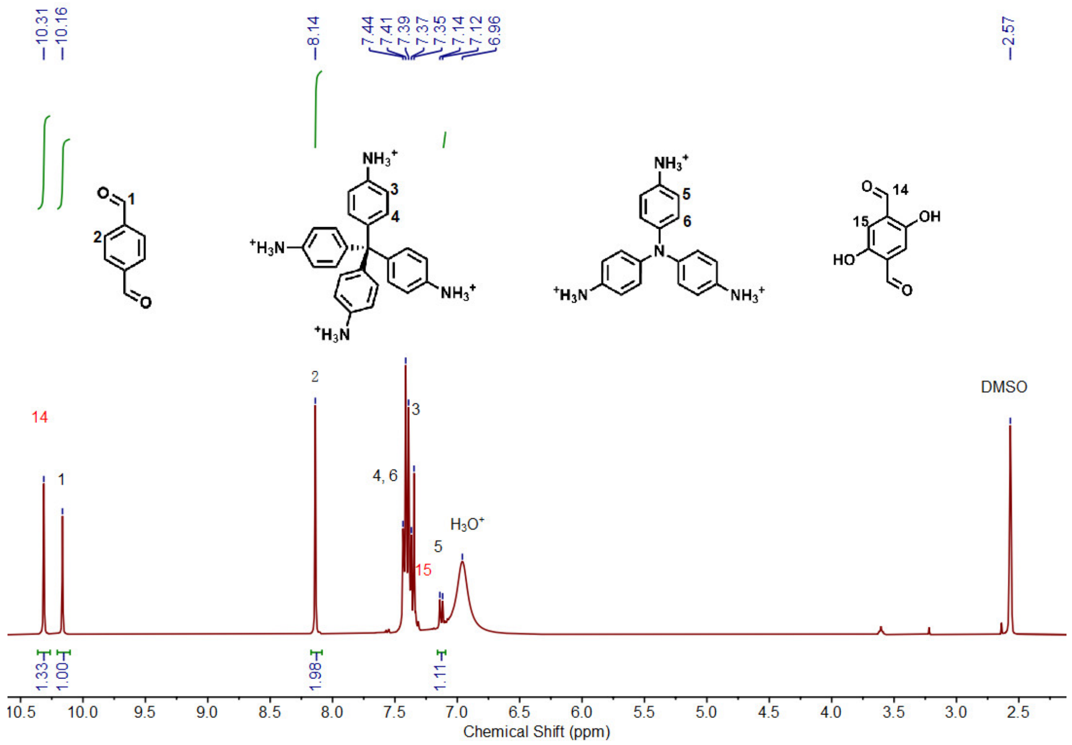

Supplementary Fig. 22 Digestion ^{1}H NMR spectrum of the activated COF-300-D-OH.

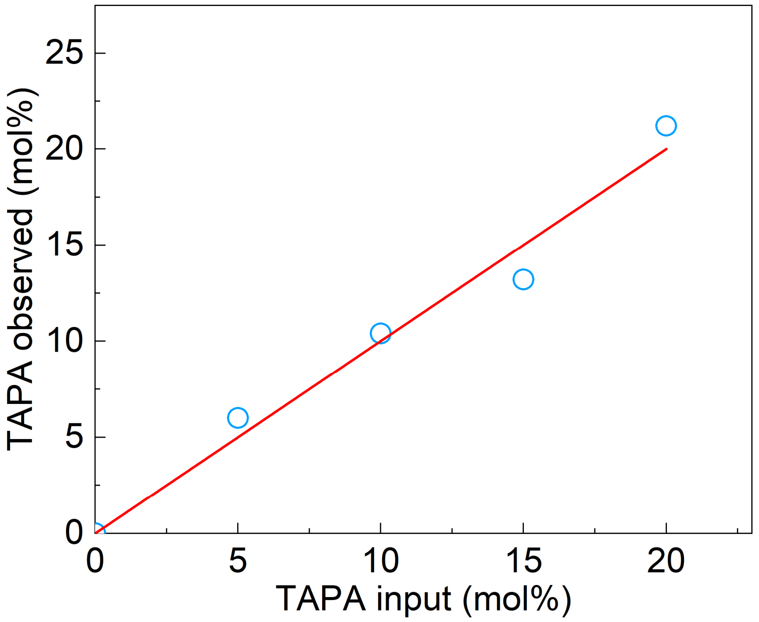

Supplementary Fig. 23 The plot of the observed ratio of TAPA linker determined by ^{1}H NMR versus input ratio in the COF-300-D series.

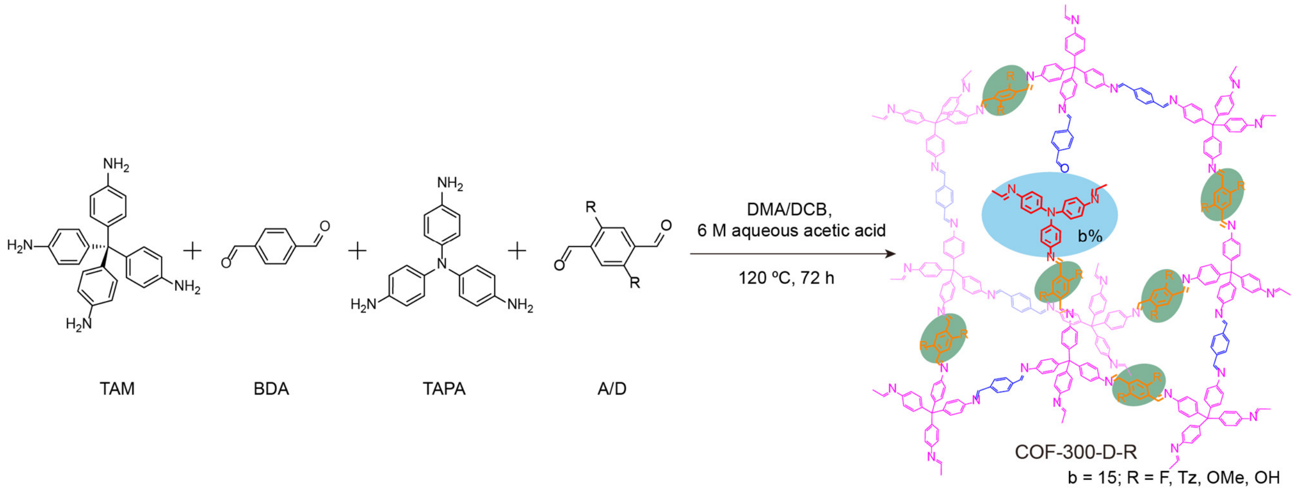

Supplementary Fig. 24 Schematic diagram of the synthesis of COF-300-D-R (D =15%; R = F, Tz, OMe, OH).

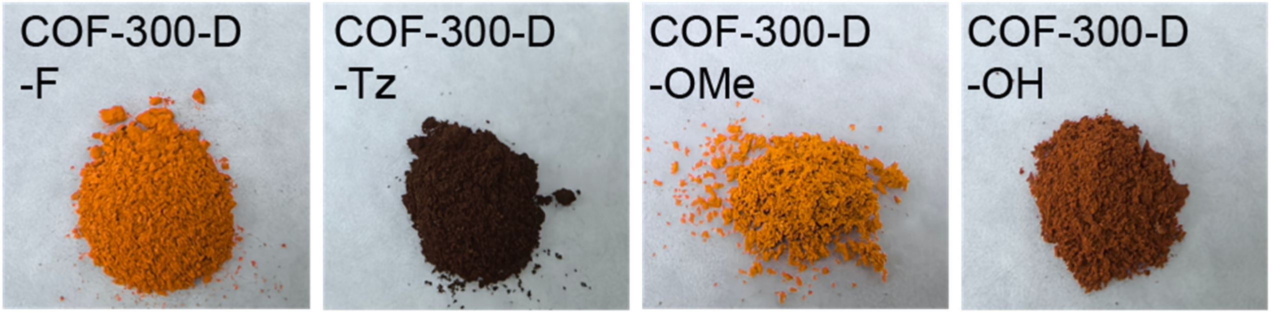

Supplementary Fig. 25 Optical pictures of COF-300-D-F, COF-300-D-Tz, COF-300-D-OMe, and COF-300-D-OH, respectively.

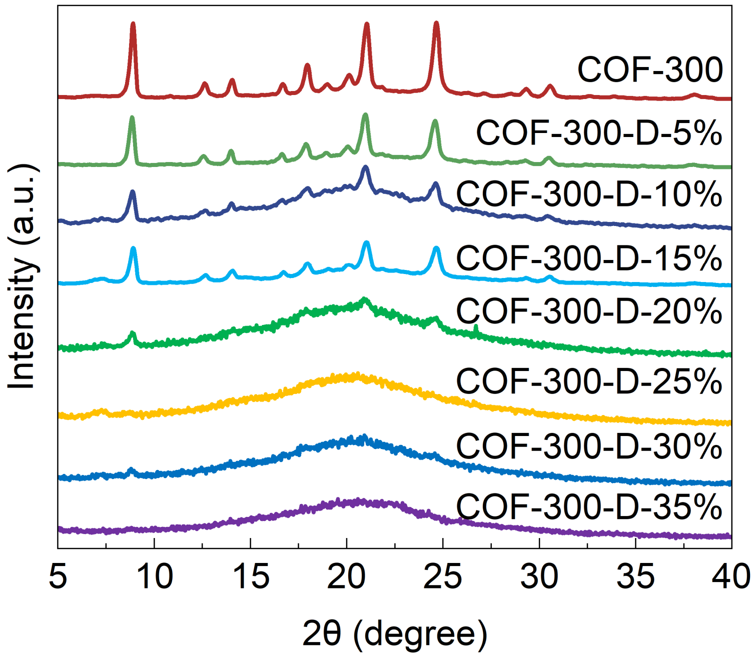

Supplementary Fig. 26 Experimentally obtained PXRD patterns of COFs.

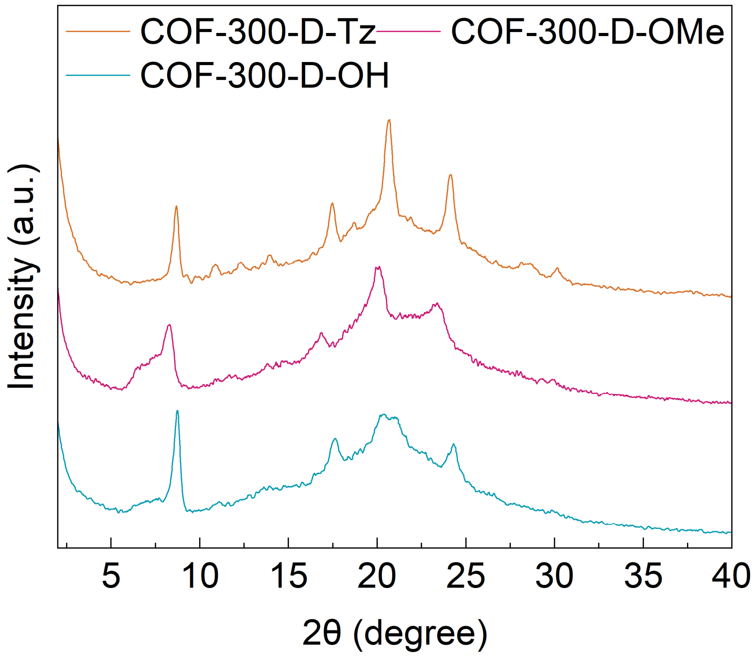

Supplementary Fig. 27 PXRD patterns of COF-300-D-Tz, COF-300-D-OMe, and COF-300-D-OH.

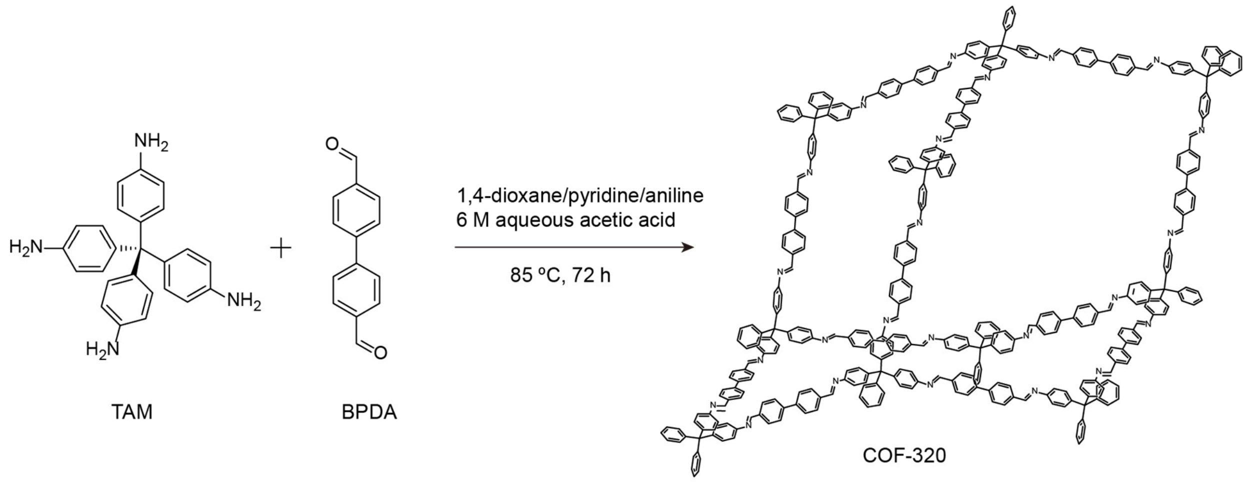

Supplementary Fig. 28 Schematic diagram of the synthesis of COF-320.

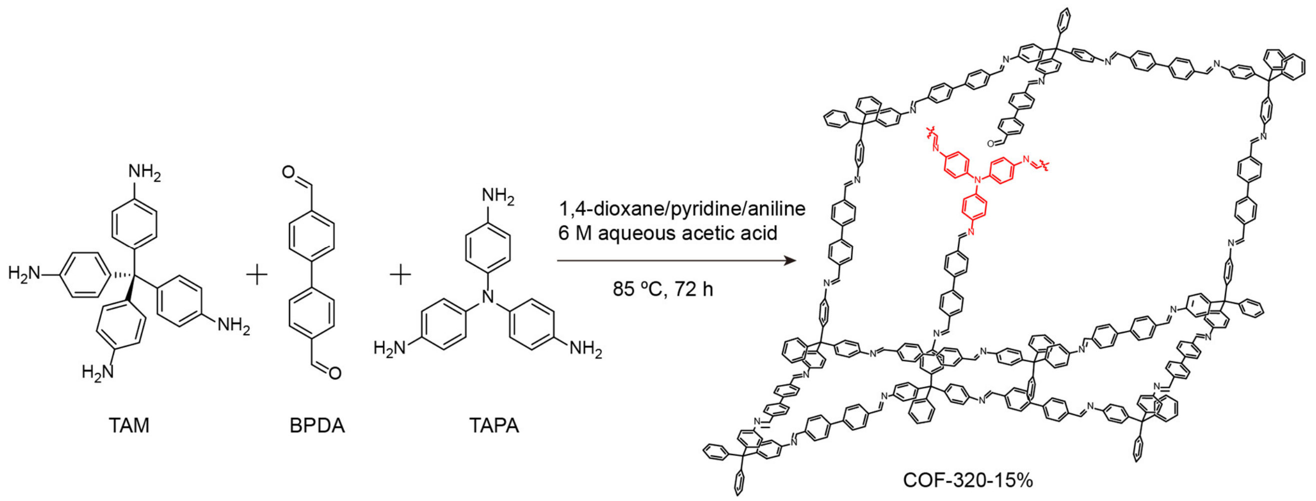

Supplementary Fig. 29 Schematic diagram of the synthesis of COF-320-D-15%. To validate the universal applicability of this defect engineering, COF-320-D-15% and COF-320-D-F were synthesized using an identical protocol.

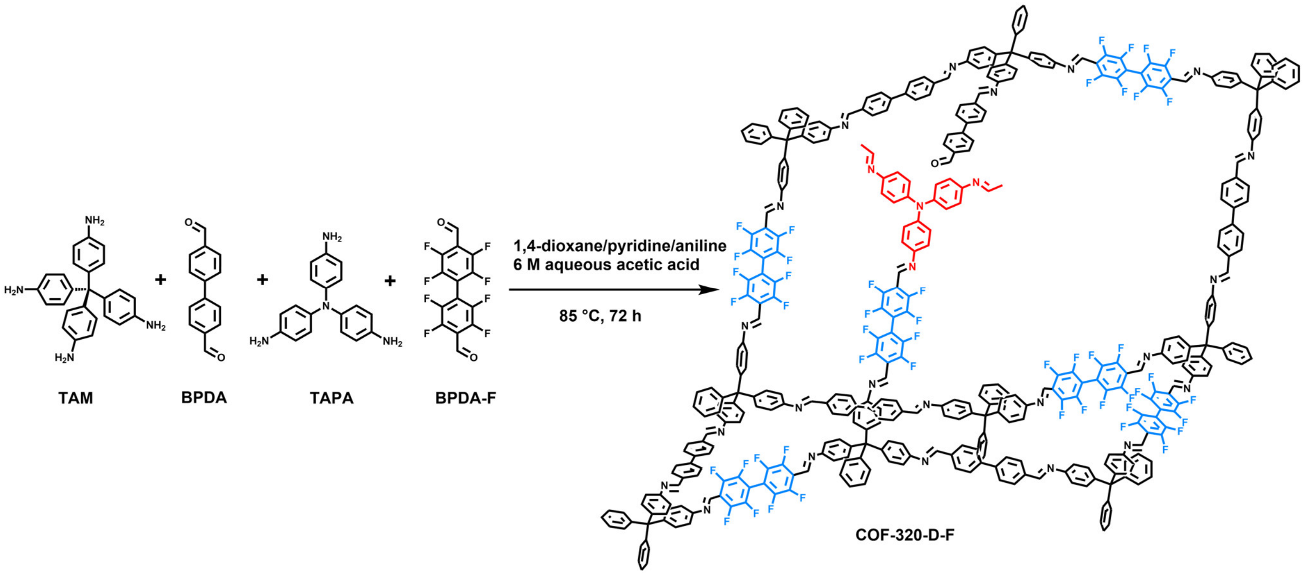

Supplementary Fig. 30 Schematic diagram of the synthesis of COF-320-D-F. To validate the universal applicability of this defect engineering, COF-320-D-15% and COF-320-D-F were synthesized using an identical protocol.

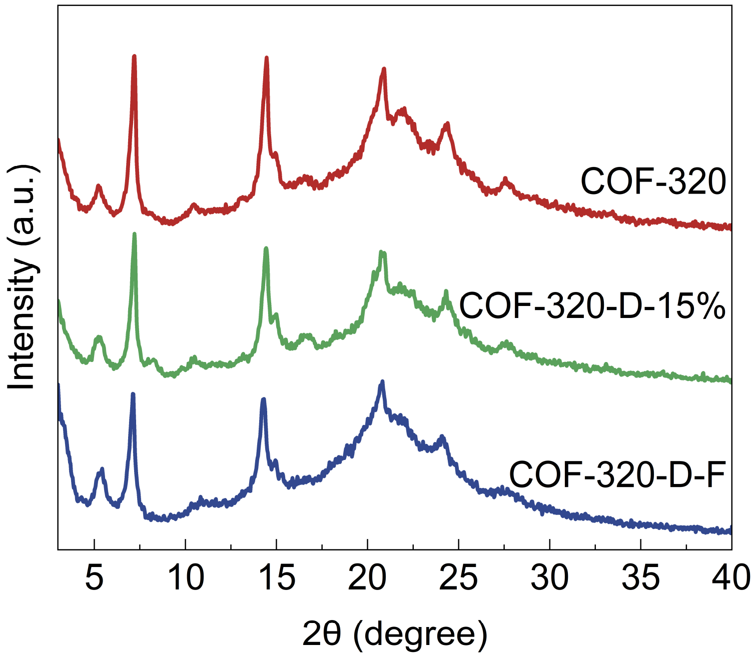

Supplementary Fig. 31 Experimental PXRD patterns of COF-320, COF-320-D-15%, and COF-320-D-F. Structural characterization revealed well-preserved crystallinity with minimal structural alterations.

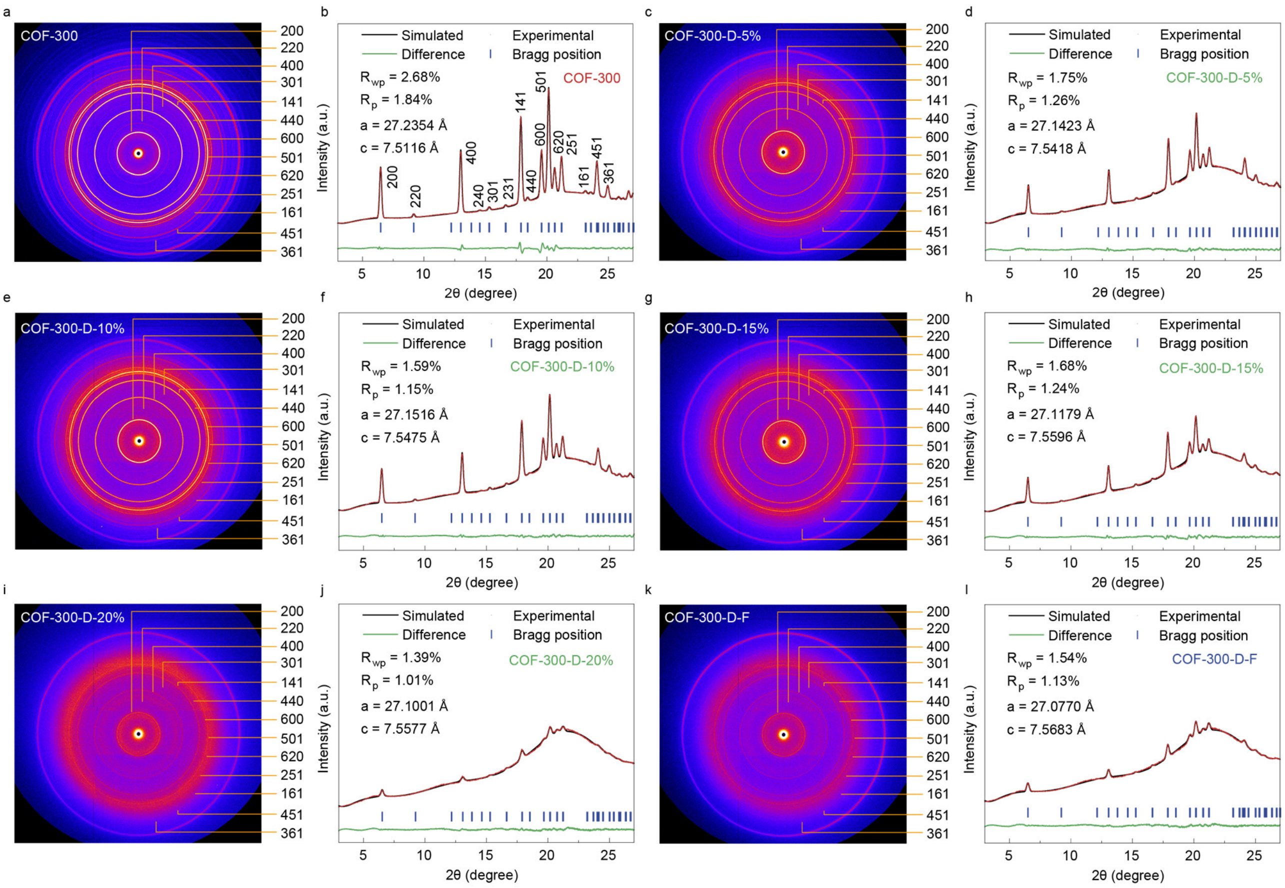

Supplementary Fig. 32 The SAXS characterization of COFs. (**a, c, e, g, i, k**) 2D SAXS image of COFs (λ = 1.54056 Å). (**b, d, f, h, j, l)** Experimentally obtained SAXS results (red dot), Pawley refined patterns (black curve), their differences (green curve), and Bragg position (blue bars) of COFs.

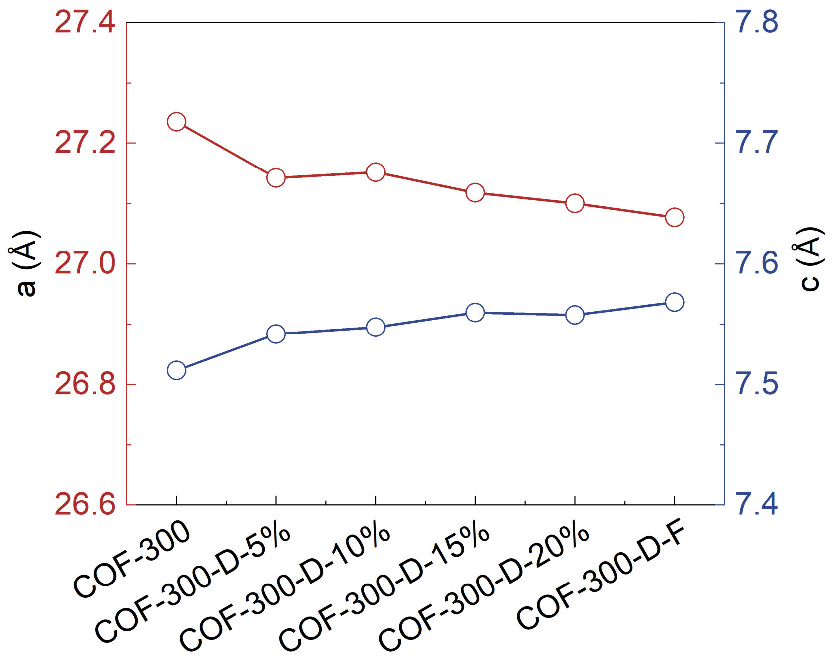

Supplementary Fig. 33 Systematic changes of unit cell parameters ***a*** and ***c*** of COF-300 and its derivatives obtained from Pawley refinements based on SAXS patterns.

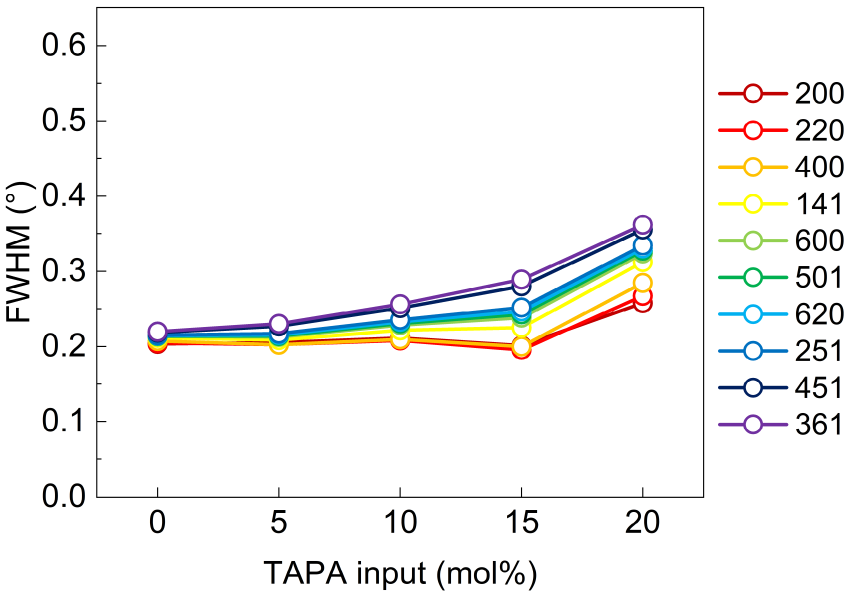

Supplementary Fig. 34 Full width at half maximum (FWHM) of the main diffraction peaks in the SAXS patterns.

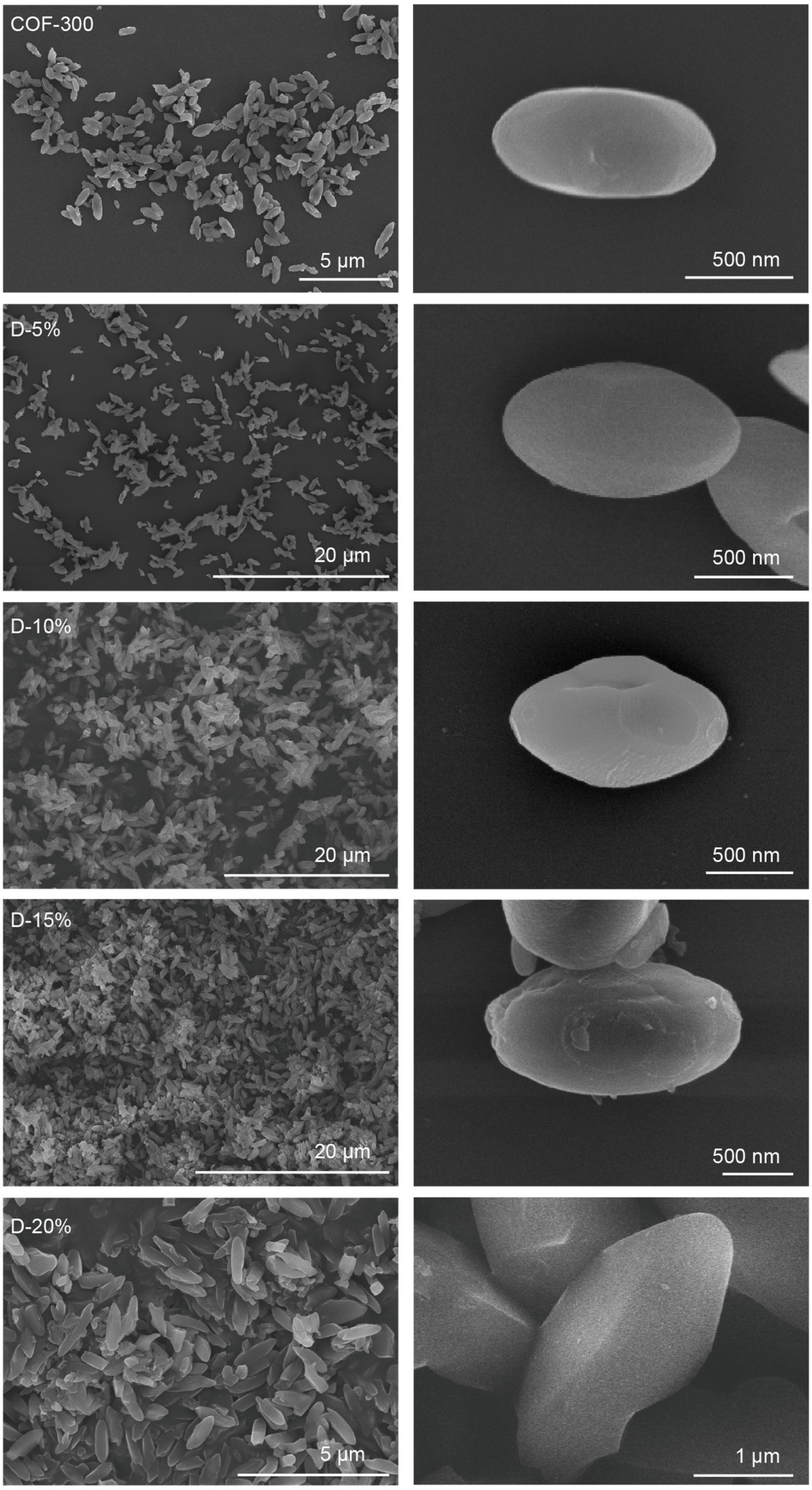

Supplementary Fig. 35 SEM images of COF-300, COF-300-D-5%, COF-300-D-10%, COF-300-D-15%, and COF-300-D-20%, respectively. Experiments were repeated at least three times independently with similar results.

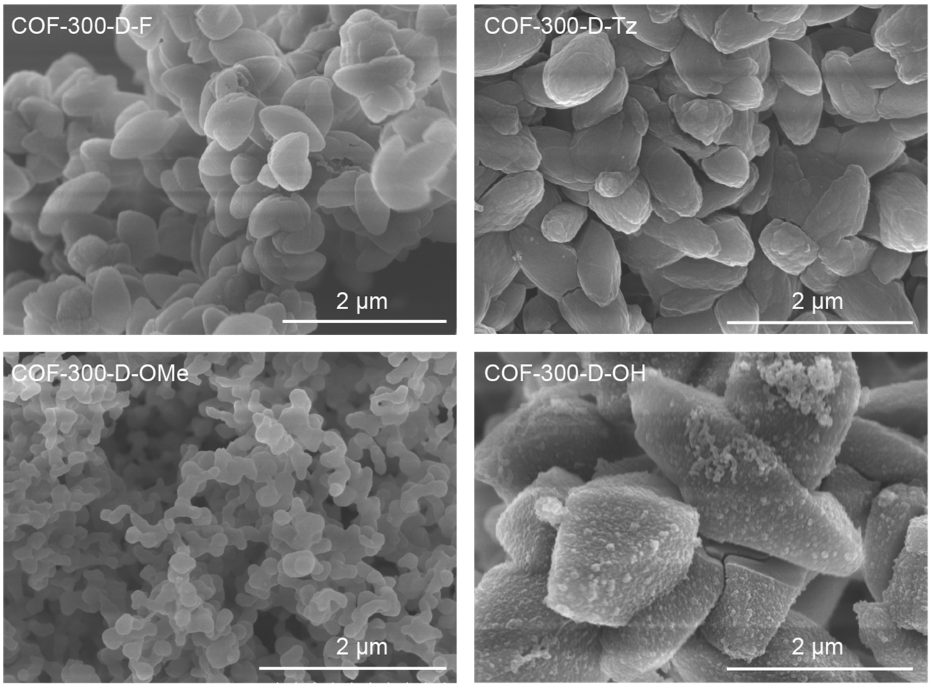

Supplementary Fig. 36 SEM images of COF-300-D-F, COF-300-D-Tz, COF-300-D-OMe, and COF-300-D-OH, respectively. Experiments were repeated at least three times independently with similar results.

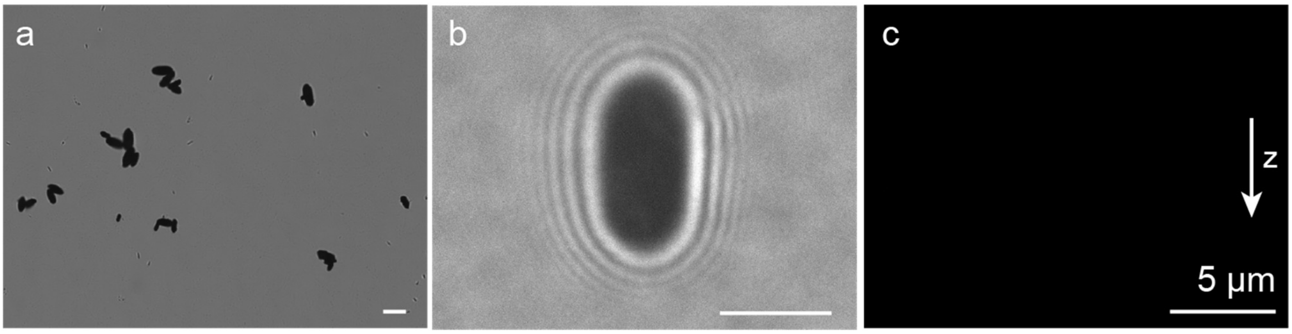

Supplementary Fig. 37 3D tomography of COF-300 by laser scanning confocal microscopy, scale bar 5 μm. a, b, Bright field image, **c,** Consecutive LSCM sections of COF-300. COF-300 exhibits no fluorescence under excitation light of 405 nm. Experiments were repeated at least three times independently with similar results.

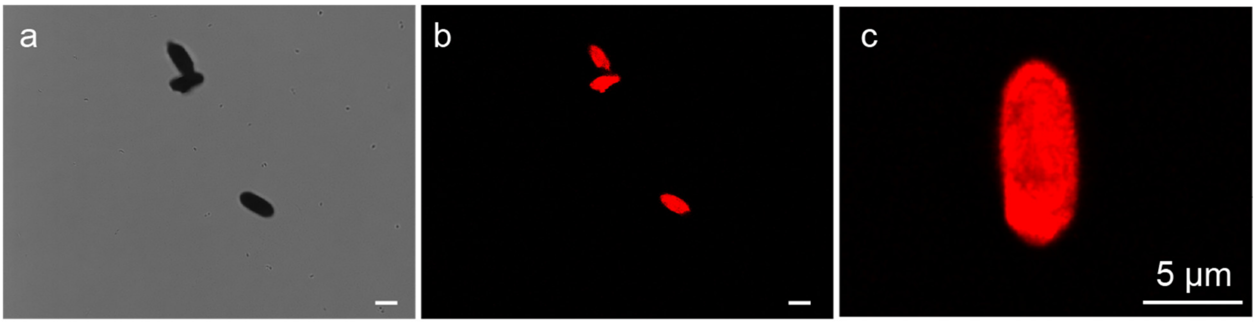

Supplementary Fig. 38 3D tomography of COF-300-D-15% by laser scanning confocal microscopy, scale bar 5 μm. a, Bright field image, **b,** LSCM image, **c,** One slice of consecutive LSCM sections of COF-300-D-15%. Red fluorescence was observed in all layers under excitation light at 405 nm, indicating a homogeneous distribution of TAPA across the entire crystal. Experiments were repeated at least three times independently with similar results.

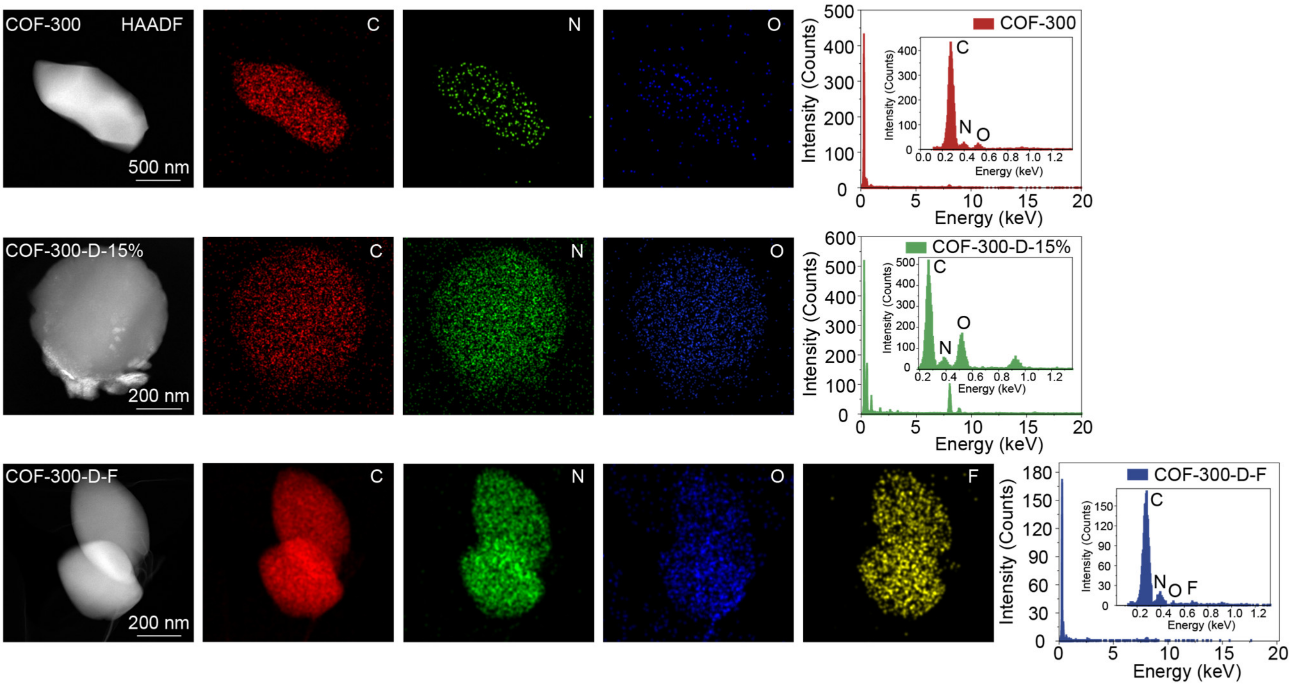

Supplementary Fig. 39 High-angle annular dark field image (HAADF) images and energy dispersive spectroscopy (EDS) elemental mappings of COF-300, COF-300-D-15%, and COF-300-D-F, respectively. Experiments were repeated at least three times independently with similar results.

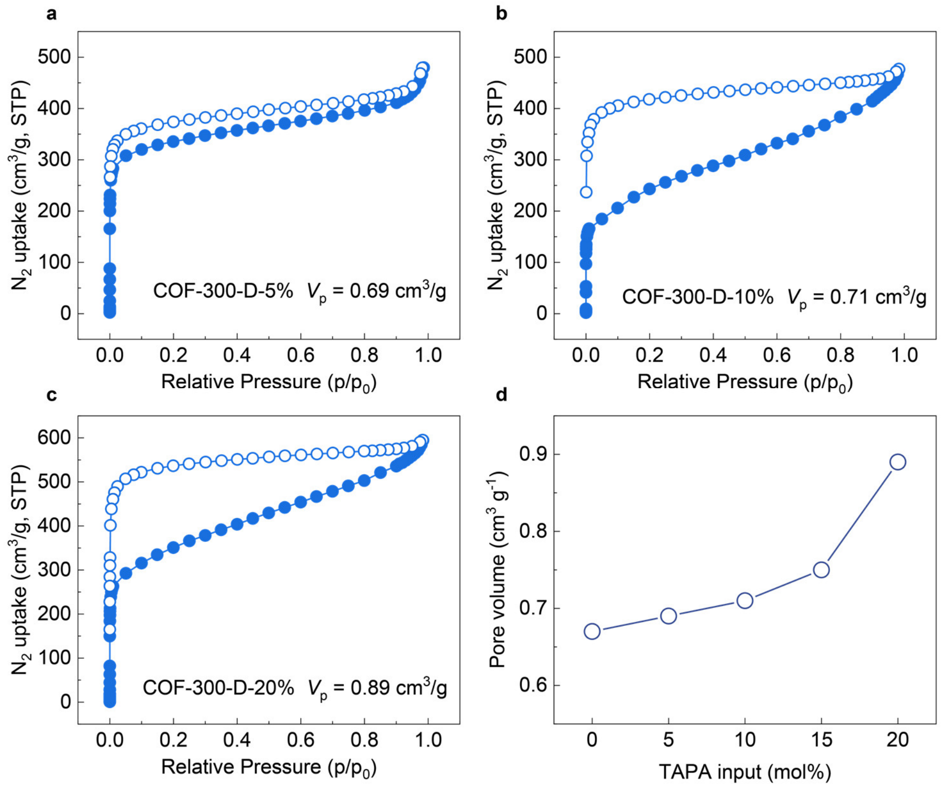

Supplementary Fig. 40 Nitrogen sorption isotherms of COFs. (a-c) Nitrogen sorption isotherms of COF-300-D-5% (**a**), COF-300-D-10% (**b**), and COF-300-D-20% (**c**) (solid circle, adsorption; open circle, desorption). *P*, pressure; P_0, saturation pressure. **d,** Pore volumes of COF-300 and COF-300-D series increased gradually while the TAPA content increased.

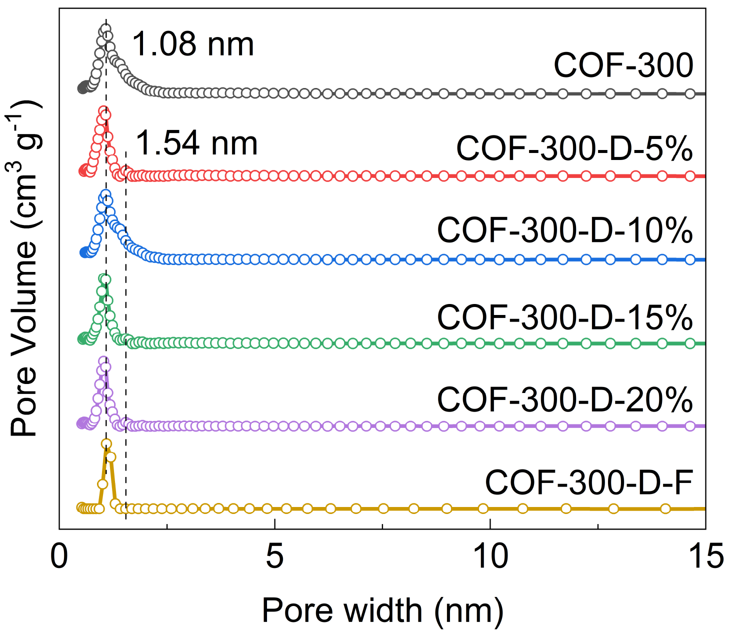

Supplementary Fig. 41 Pore size distributions of COFs.

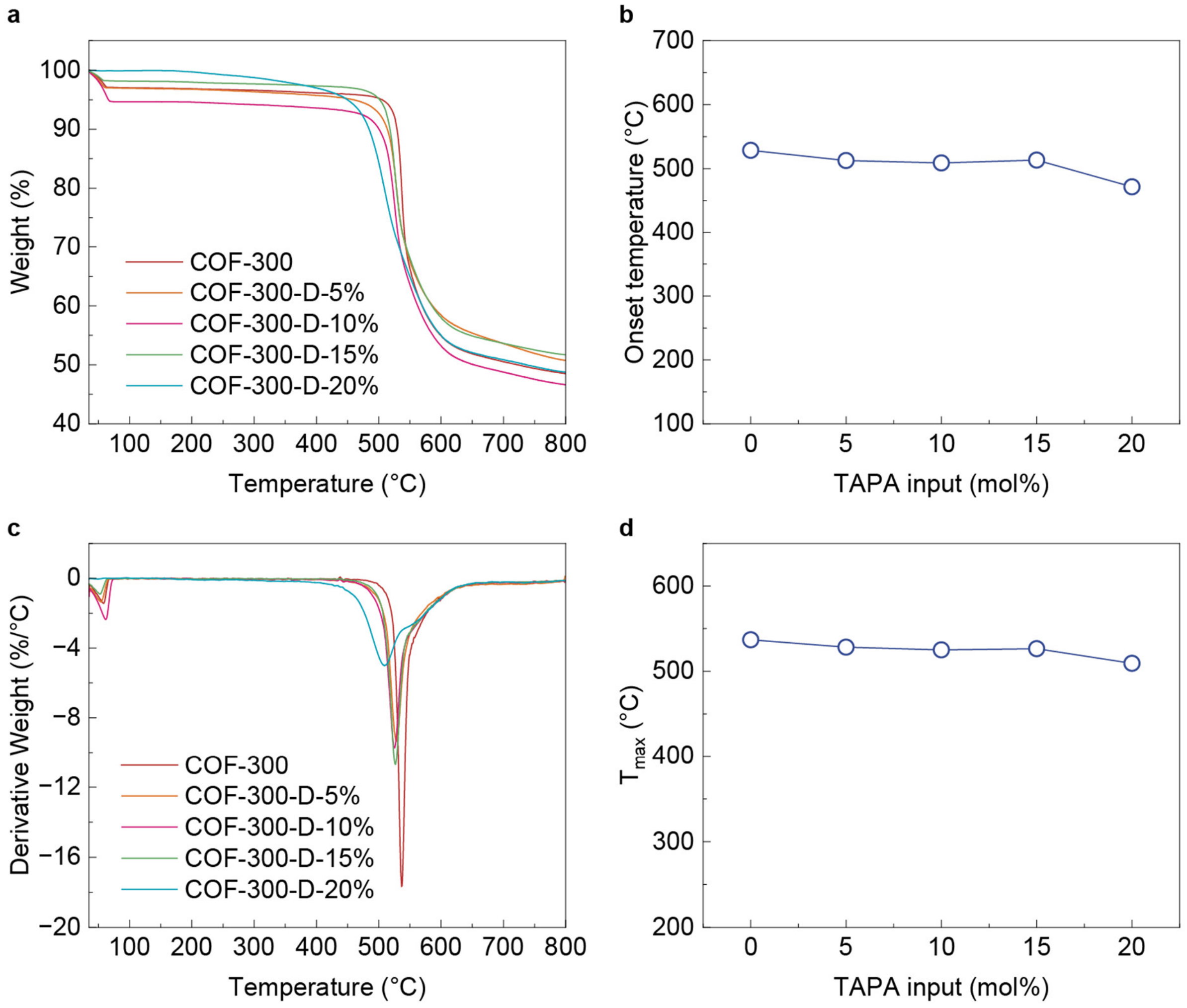

Supplementary Fig. 42 TGA trace of COFs. a, TGA trace of the activated COF-300, COF-300-D-5%, COF-300-D-10%, COF-300-D-15%, and COF-300-D-20%, respectively. **b,** The plot of the initial decomposition temperature of the COF-300-D and the amount of TAPA (0%~20%) introduced was plotted. **c,** DTG trace of the activated COF-300, COF-300-D-5%, COF-300-D-10%, COF-300-D-15%, and COF-300-D-20%, respectively. **d,** The plot of the fastest decomposition temperature of the COF-300-D and the amount of TAPA (0%~20%) introduced was plotted.

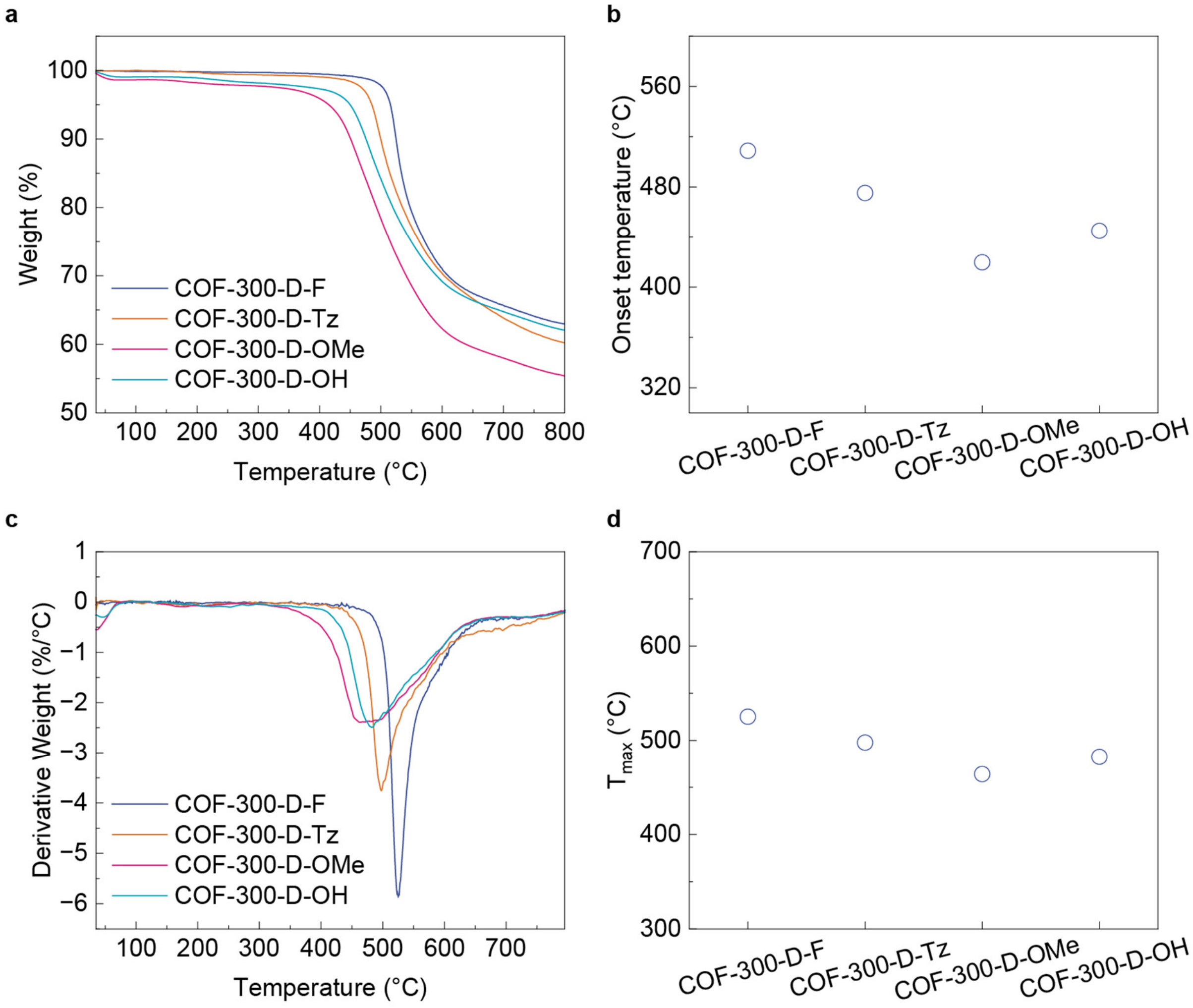

Supplementary Fig. 43 TGA trace of COFs. a, TGA trace of the activated COF-300-D-F, COF-300-D-Tz, COF-300-D-OMe, and COF-300-D-OH, respectively. **b,** Initial decomposition temperature of the activated COF-300-D-F, COF-300-D-Tz, COF-300-D-OMe, and COF-300-D-OH, respectively. **c,** DTG trace of the activated COF-300-D-F, COF-300-D-Tz, COF-300-D-OMe, and COF-300-D-OH, respectively. **d,** Fastest decomposition temperature of the activated COF-300-D-F, COF-300-D-Tz, COF-300-D-OMe, and COF-300-D-OH, respectively.

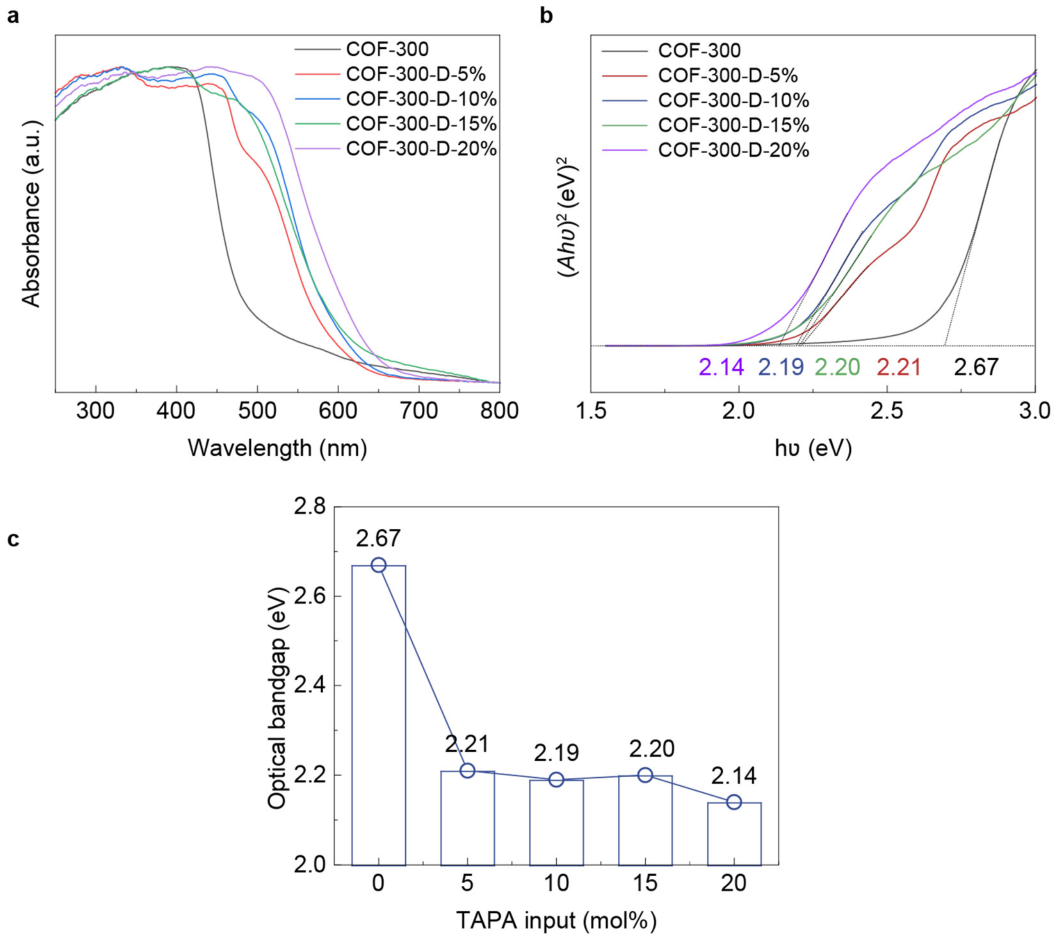

Supplementary Fig. 44 UV–vis DRS and optical bandgap characterization of COF-300-D. a, UV–vis DRS of COF-300, COF-300-D-5%, COF-300-D-10%, COF-300-D-15%, and COF-300-D-20%. **b,** Tauc plot (annotated values are the optical bandgap). *A*, absorption coefficient; h, Planck constant; υ, frequency. **c,** The plot of the optical bandgap of the COF-300-D and the amount of TAPA (0%~20%) introduced was plotted.

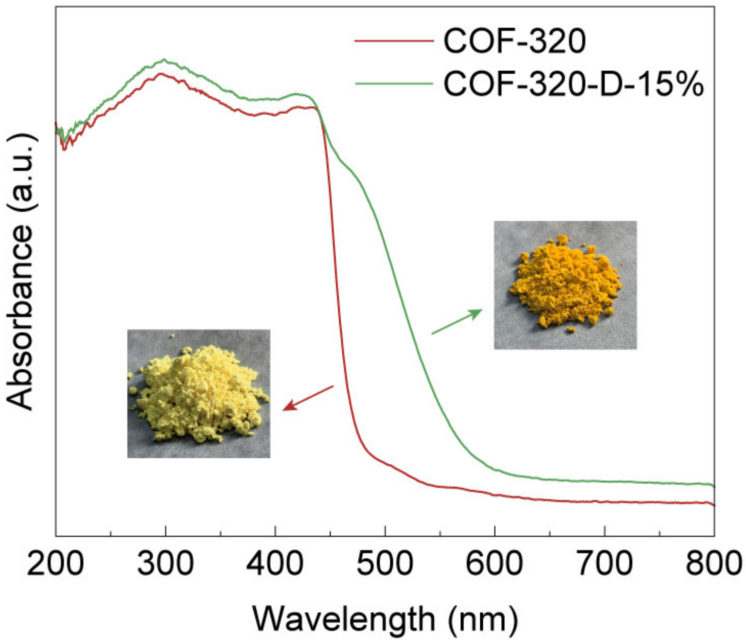

Supplementary Fig. 45 UV–vis DRS of COF-320 and COF-320-D-15%.

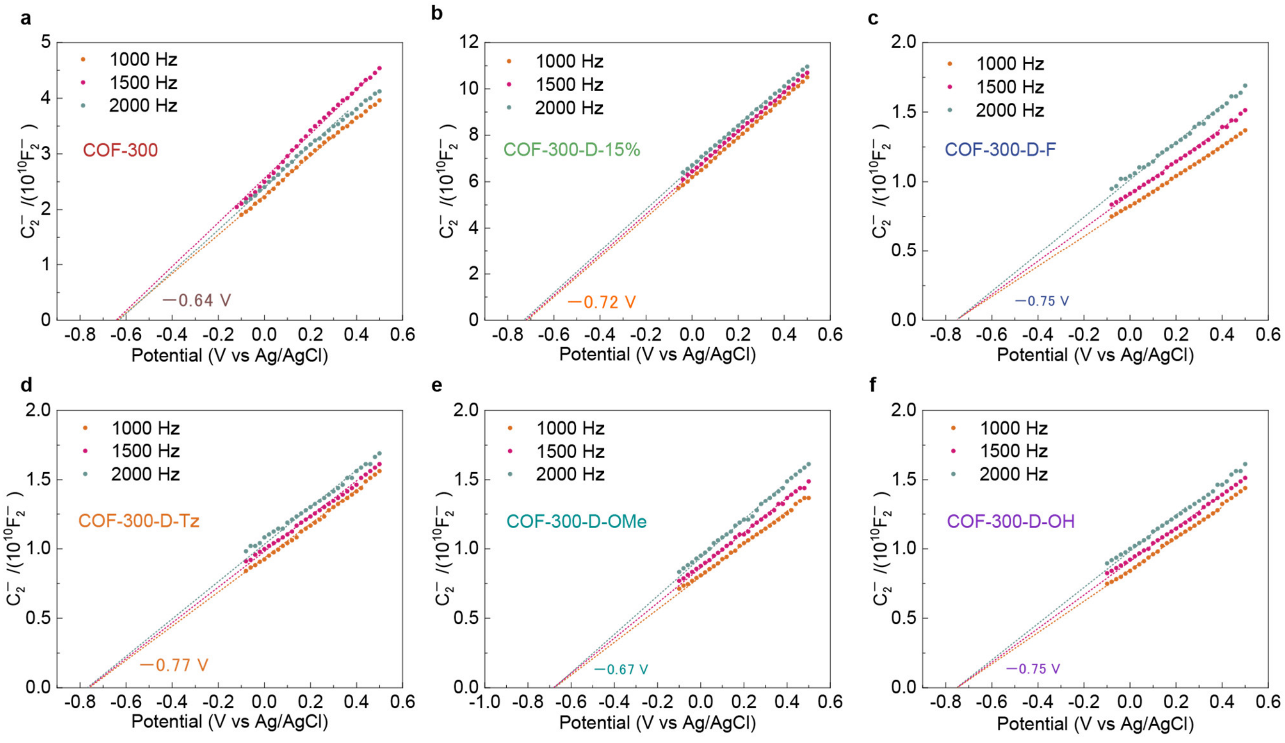

Supplementary Fig. 46 Mott-Schottky test. a-f, Mott-Schottky plots of COF-300 (**a**), COF-300-D-15% (**b**), COF-300-D-F (**c**), COF-300-D-Tz (**d**), COF-300-D-OMe (**e**), and COF-300-D-OH (**f**) at the isoelectric point. E_{CB} (V vs. NHE) was calculated according to the formula: E(NHE) = E(Ag/AgCl) + 0.197.

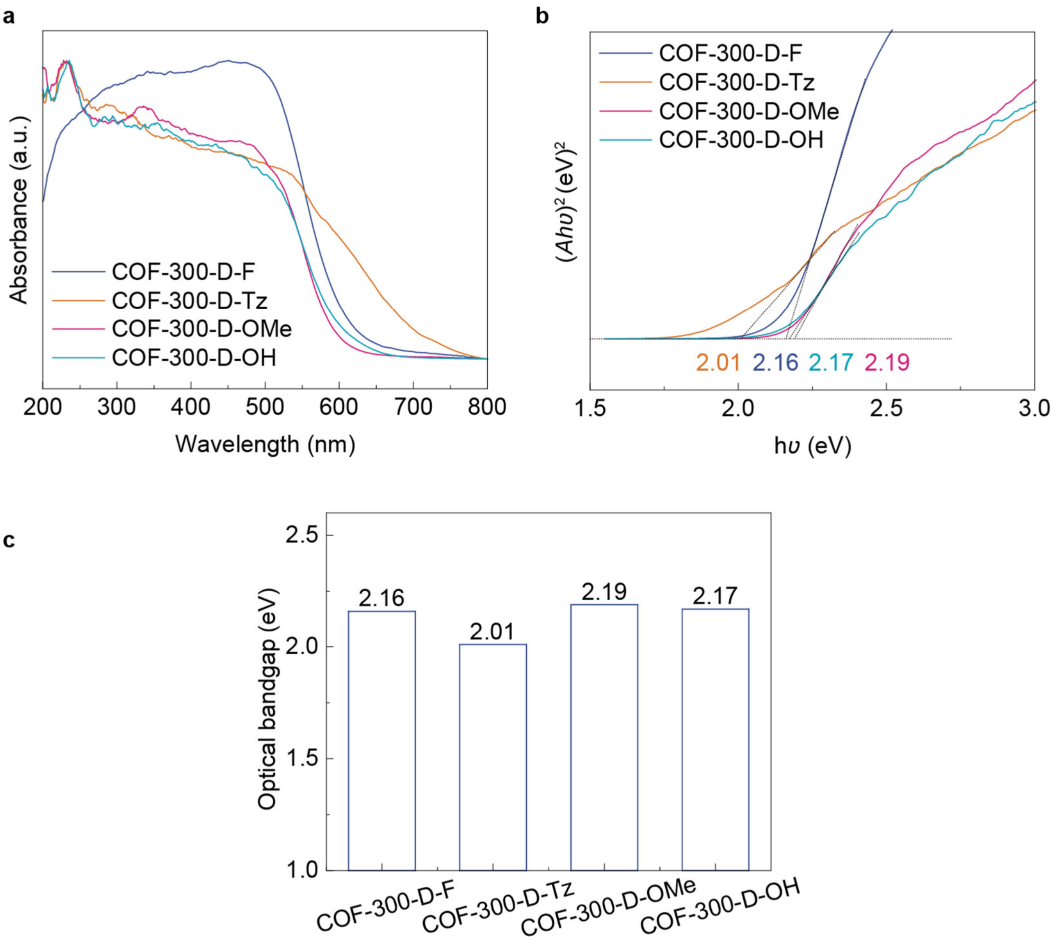

Supplementary Fig. 47 UV–vis DRS and optical bandgap characterization of COF-300-D-R. a, UV–vis DRS of COF-300-D-F, COF-300-D-Tz, COF-300-D-OMe, and COF-300-D-OH. **b,** Tauc plot (annotated values are the optical bandgap). *A*, absorption coefficient; *h*, Planck constant; *υ*, frequency. **c,** Optical bandgap of the activated COF-300-D-F, COF-300-D-Tz, COF-300-D-OMe, and COF-300-D-OH, respectively.

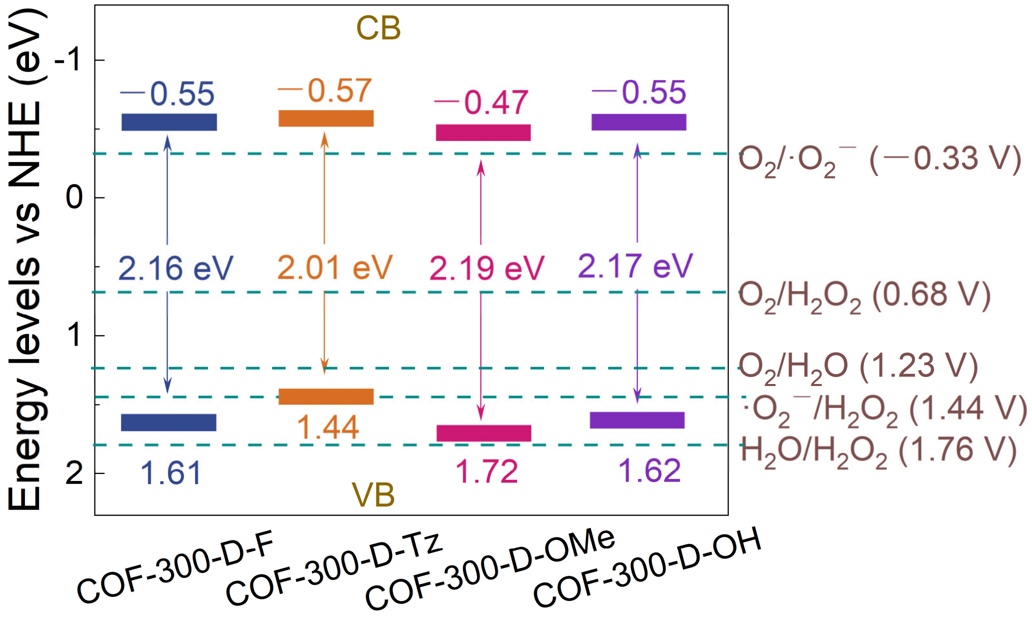

Supplementary Fig. 48 Experimentally derived energy band alignments of COFs compared to oxygen reduction (–0.33 V) and oxygen evolution (+1.23 V) potentials, along with other reactions.

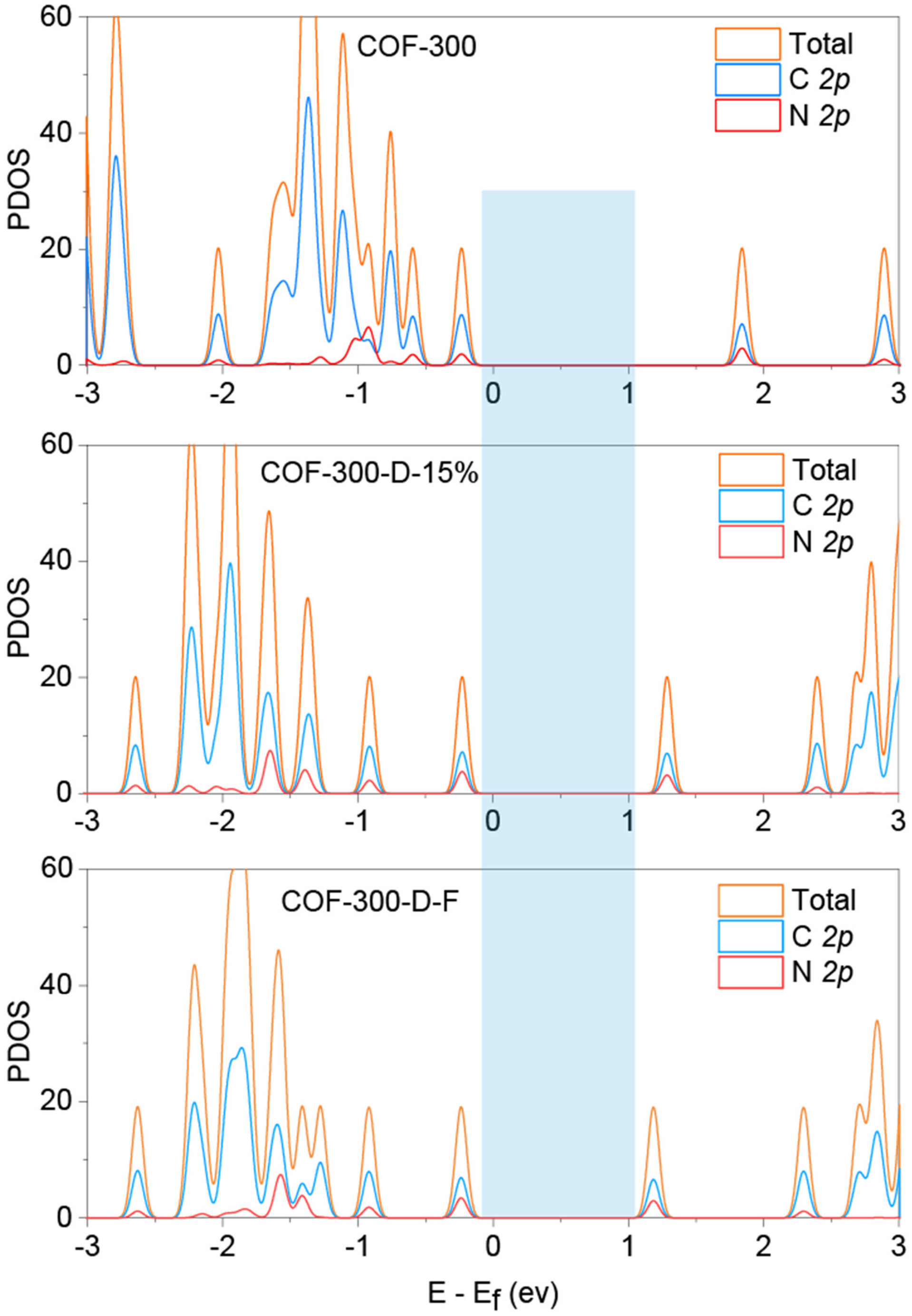

Supplementary Fig. 49 Partial density of states (PDOS) of COF-300, COF-300-D-15%, and COF-300-D-F. The PDOS, by theoretical calculation, suggests COF-300-D-F exhibits the narrowest bandgap among the series, a finding that is corroborated by experimental band structure analysis.

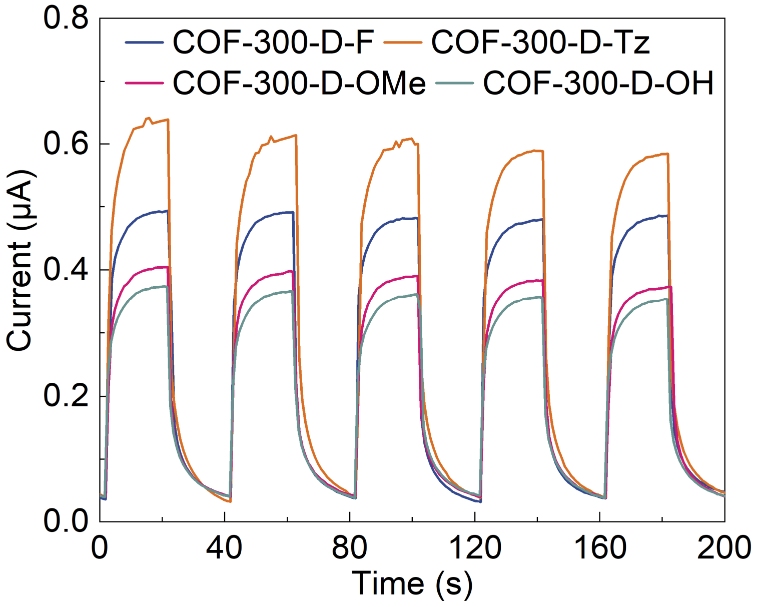

Supplementary Fig. 50 Photocurrent density measurements of COF-300-D-F, COF-300-D-Tz, COF-300-D-OMe, and COF-300-D-OH.

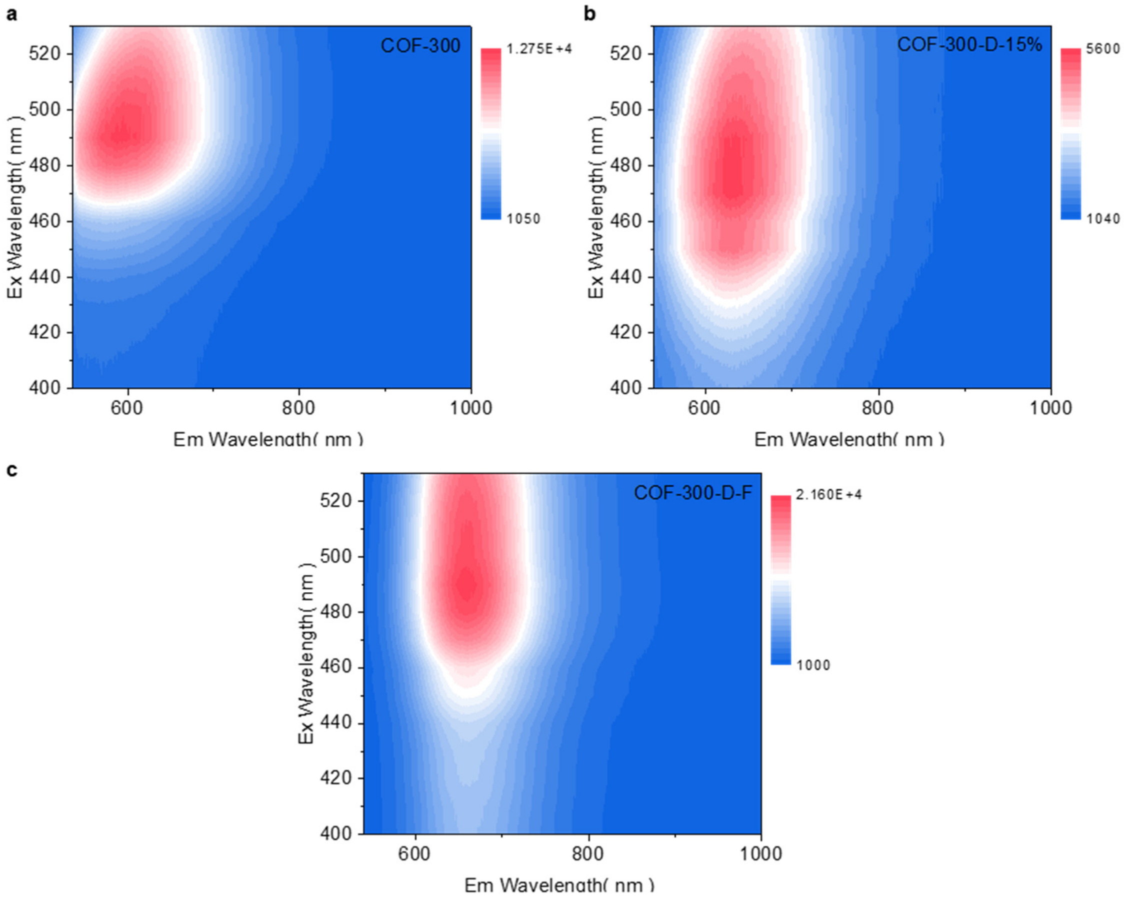

Supplementary Fig. 51 Three-dimensional fluorescence spectrum of COF-300 (**a**), COF-300-D-15% (**b**), and COF-300-D-F (**c**).

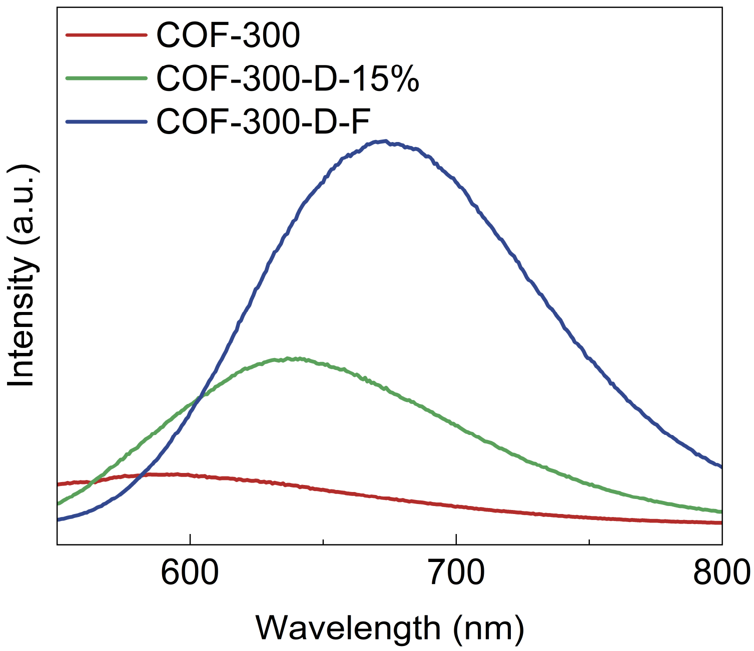

Supplementary Fig. 52 PL spectra of these three COFs at the excitation wavelength of 480 nm. The illustration is the normalized spectrum.

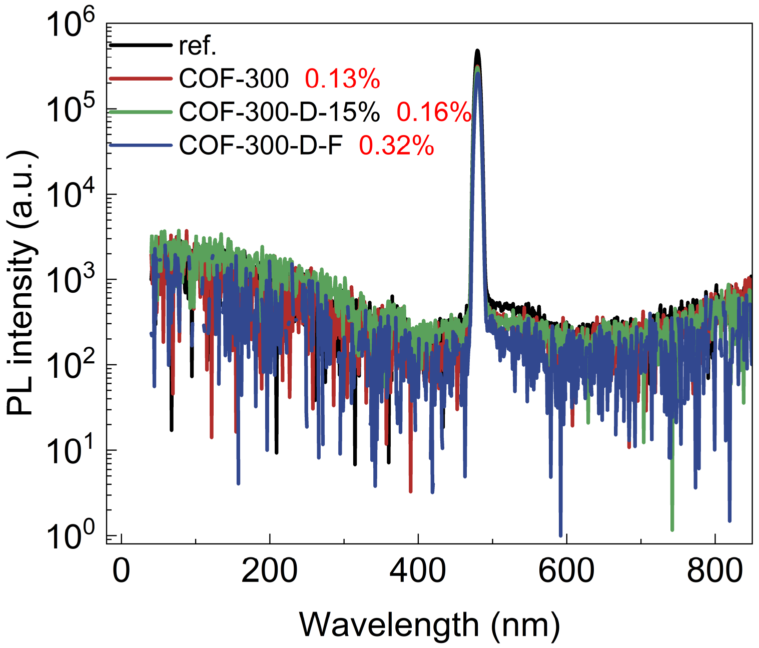

Supplementary Fig. 53 PLQY excited at 480 nm laser.

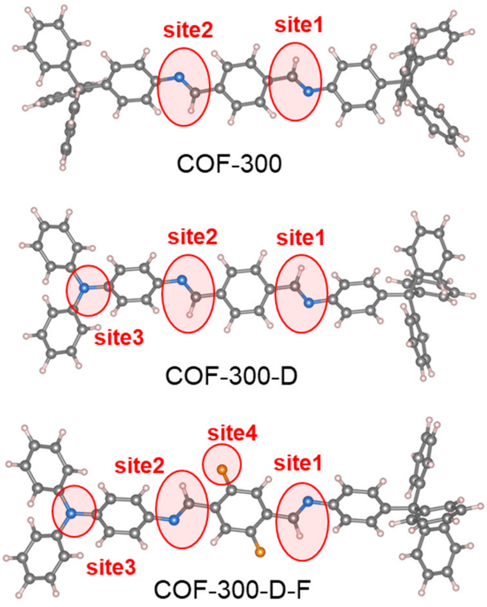

Supplementary Fig. 54 Different potential oxygen adsorption sites (Sites 1-4) were identified within the central building blocks of these COFs as determined by computational modeling. F, N, C, and H atoms are marked in orange, blue, grey and pink, respectively.

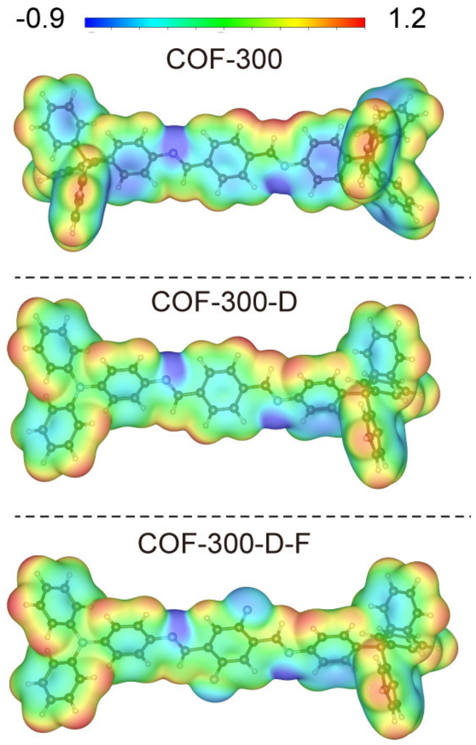

Supplementary Fig. 55 Electrostatic potential (ESP) on COF-300, COF-300-D, and COF-300-D-F, respectively.

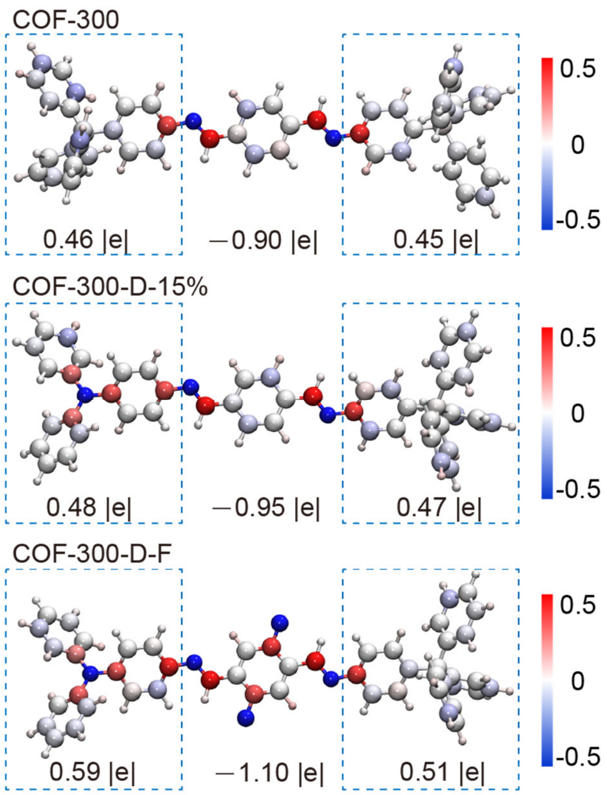

Supplementary Fig. 56 Bader charge of COF-300, COF-300-D-15%, and COF-300-D-F. Specific colors: blue (−0.5), white (0.0), red (0.5), and the middle color is defined according to the corresponding value (Bader value).

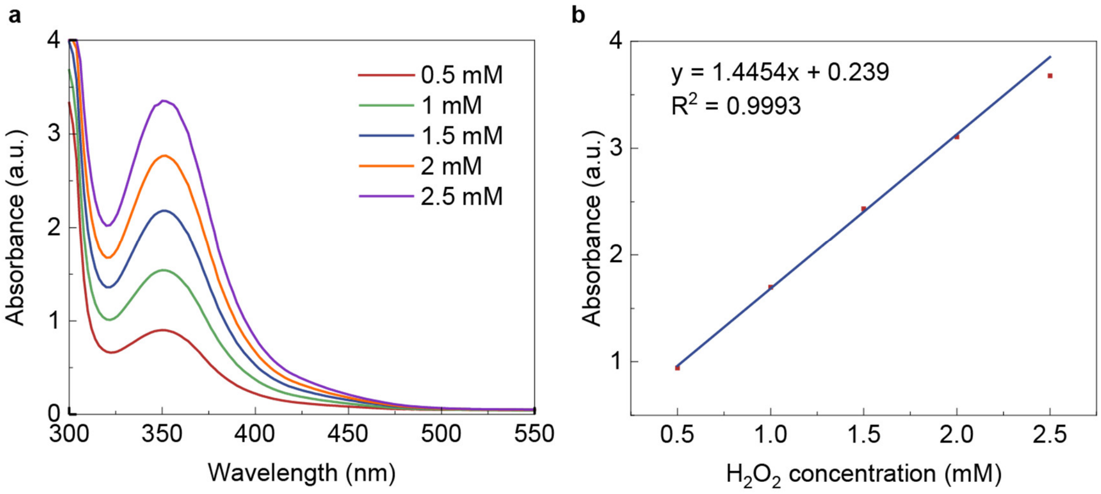

Supplementary Fig. 57 The formulation of the standard curve for Iodometric. a, A known concentration of H_2O_2 solution was added to the KI solution, and the change of absorption intensity at 350 nm was measured by a UV-vis spectrometer. **b,** The standard curve of H_2O_2 concentration-absorbance by Iodometry.

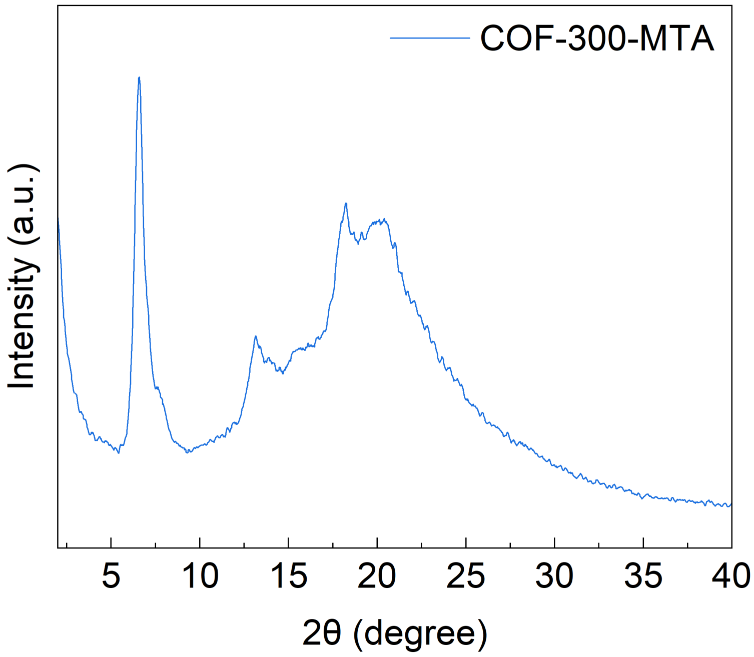

Supplementary Fig. 58 PXRD patterns of the COF-300 with 15% TAM knots replaced with tri(4-aminophenyl)methane (COF-300-MTA).

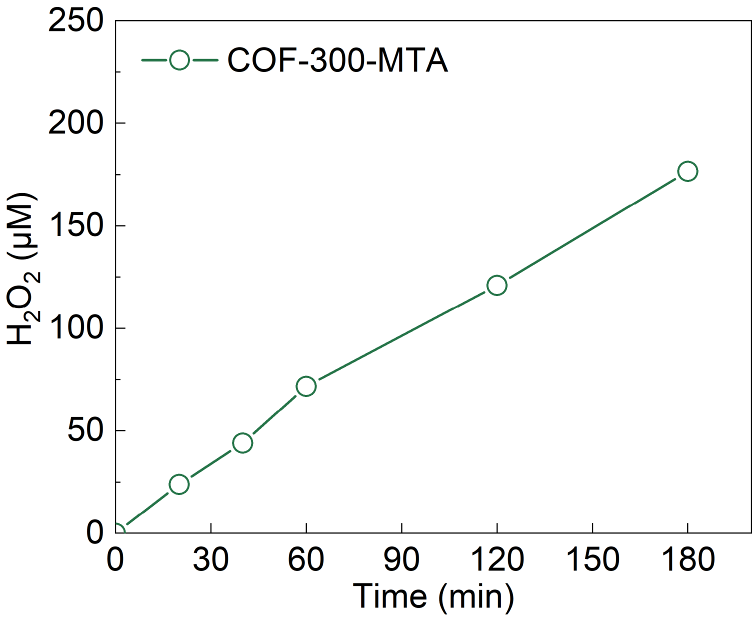

Supplementary Fig. 59 Photocatalytic activity of COF-300-MTA for H_2O_2 generation.

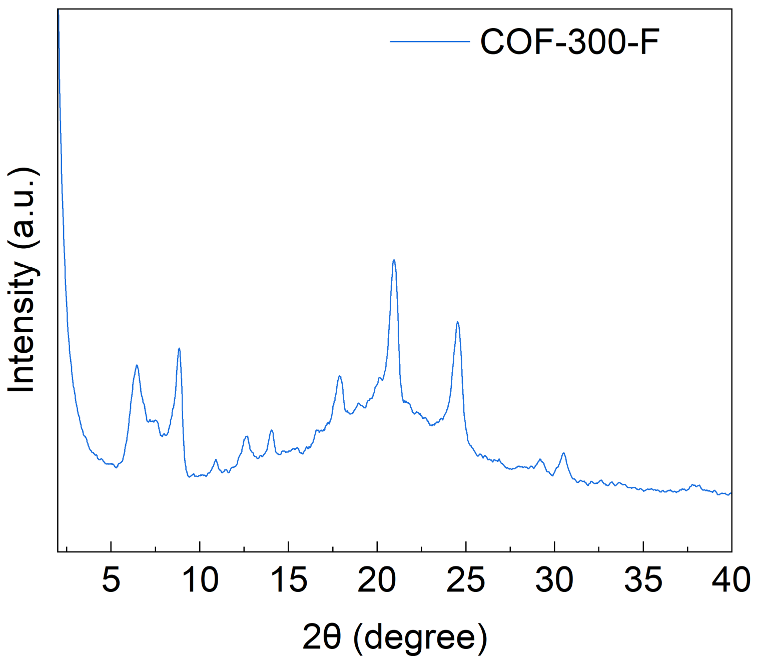

Supplementary Fig. 60 PXRD patterns of the BDA-F-based crystalline COF without TAPA (COF-300-F).

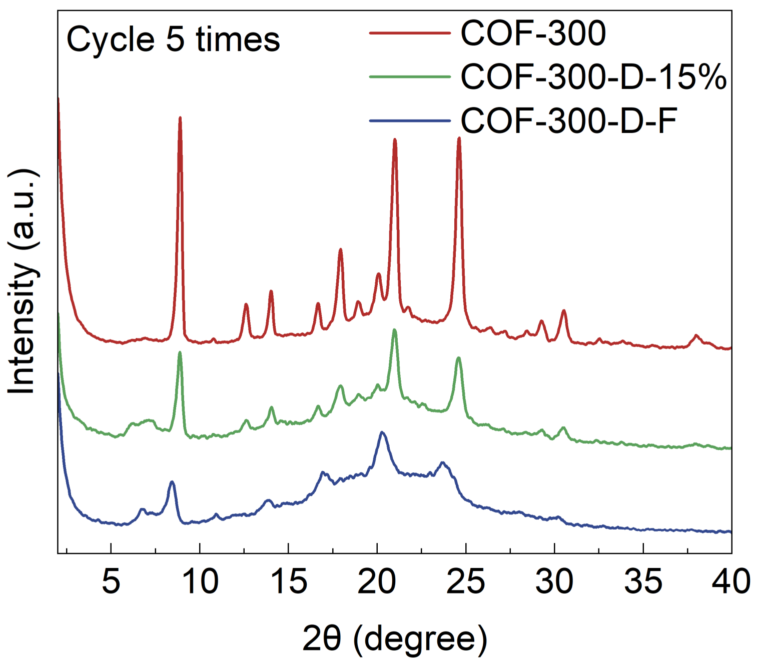

Supplementary Fig. 61 PXRD patterns of COF-300, COF-300-D-15%, and COF-300-D-F after 5 cycles of photocatalytic H_2O_2 production reaction.

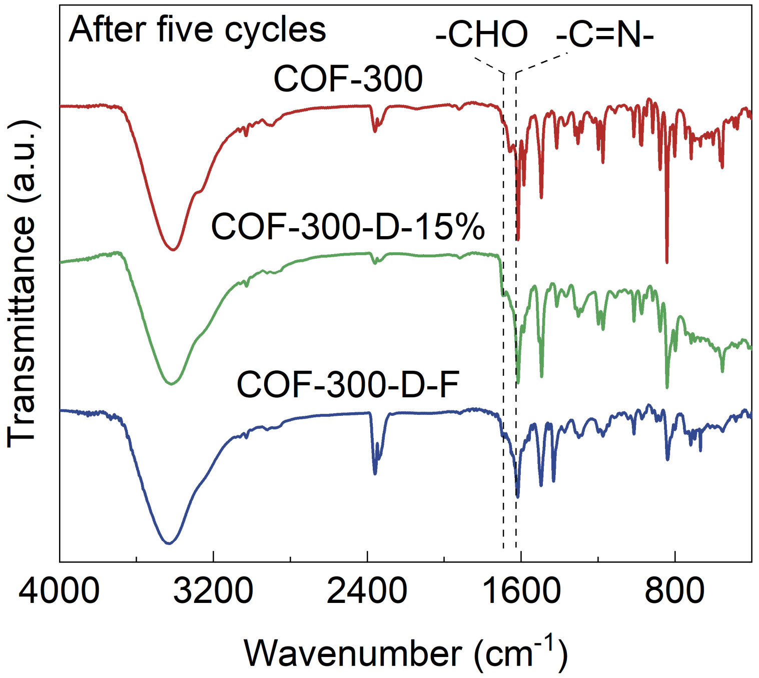

Supplementary Fig. 62 FT-IR spectra of COF-300, COF-300-D-15%, and COF-300-D-F after 5 cycles of photocatalytic H_2O_2 production reaction. The two dashed lines from left to right show the vibration bands of C=O and C=N, respectively.

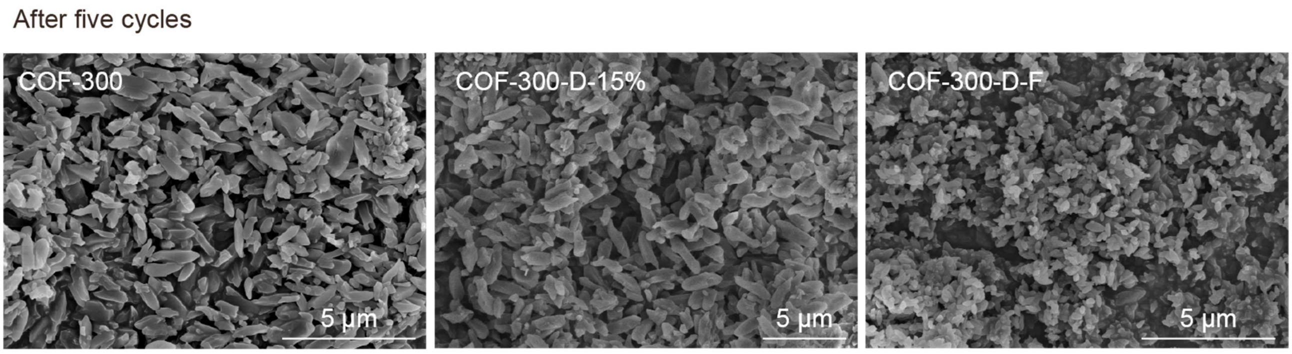

Supplementary Fig. 63 SEM images of COF-300, COF-300-D-15%, and COF-300-D-F after 5 cycles of photocatalytic H_2O_2 production reaction. Experiments were repeated at least three times independently with similar results.

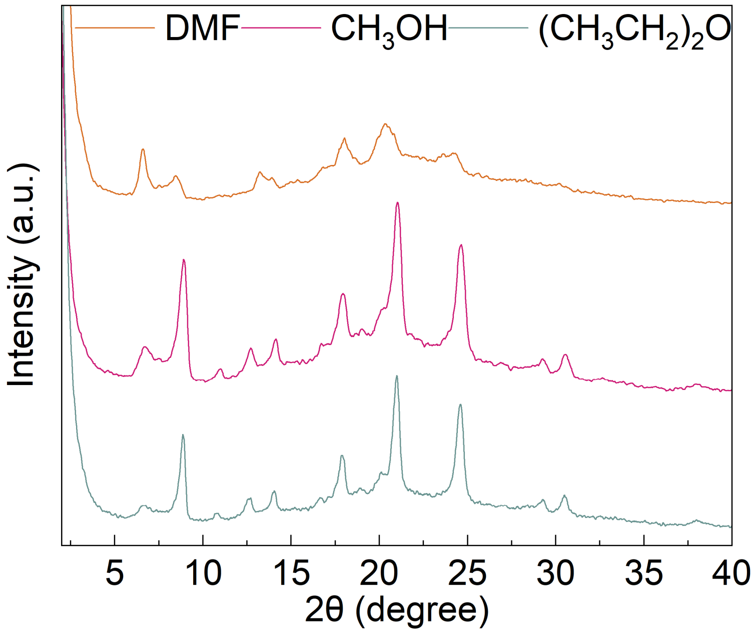

Supplementary Fig. 64 PXRD patterns of COF-300-D-F after soaking for 4 hours in N,N-dimethylformamide (DMF), methanol (CH_3OH), and diethyl ether ($(CH_3CH_2)_2O$), respectively.

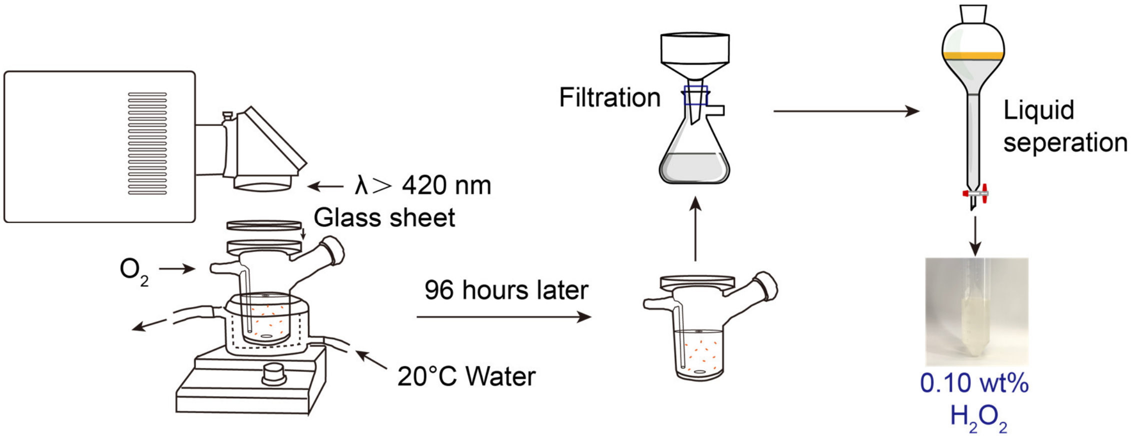

Supplementary Fig. 65 Schematic diagram of continuous and stable production of H_2O_2 over 96 hours and directly obtaining H_2O_2 solution.

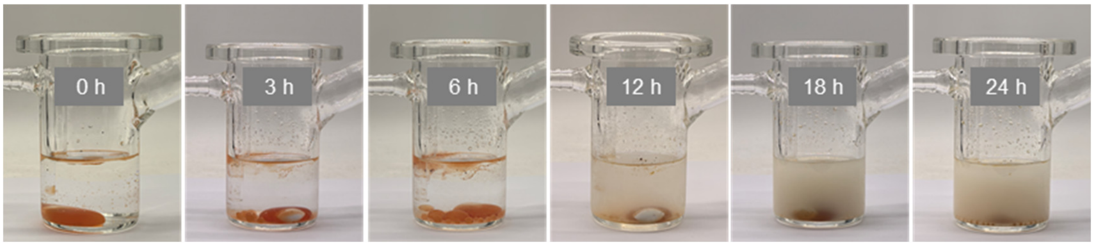

Supplementary Fig. 66 Photograph of the solution system in the long-term photocatalytic production of H_2O_2 (each image collected after stopping stirring for 10 min).

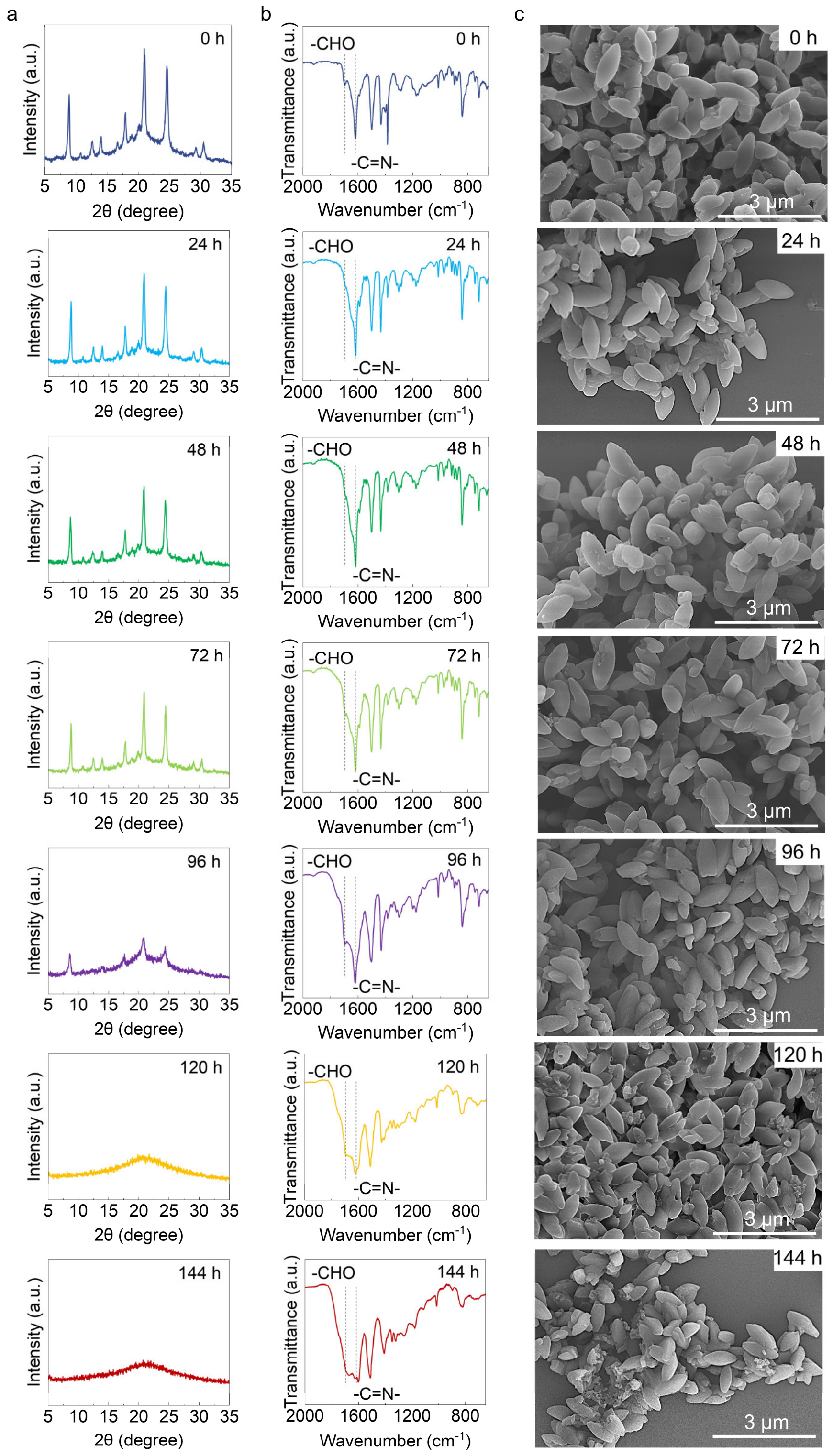

Supplementary Fig. 67 Structural stability test of COF-300-D-F after long-term photocatalysis. The long-term photocatalytic reaction of COF-300-D-F to produce H_2O_2 at different reaction times was characterized by PXRD (**a**), FTIR (**b**), and SEM (**c**). Experiments were repeated at least three times independently with similar results.

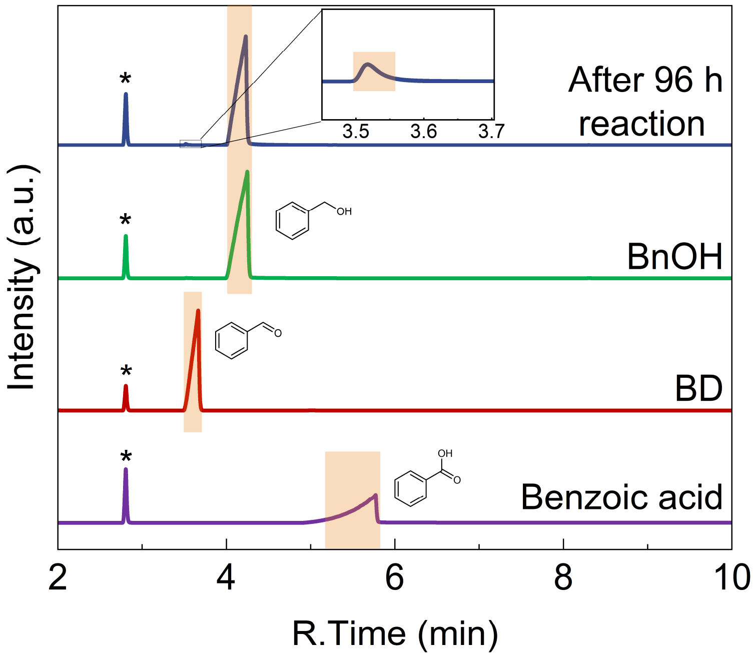

Supplementary Fig. 68 GC spectra of the reaction system solution of COF-300-D-F after 96 h of photocatalytic H_2O_2 production (benzyl alcohol as sacrificial agent) after extraction and the standard curves of benzyl alcohol, benzaldehyde, and benzoic acid. *, interior label.

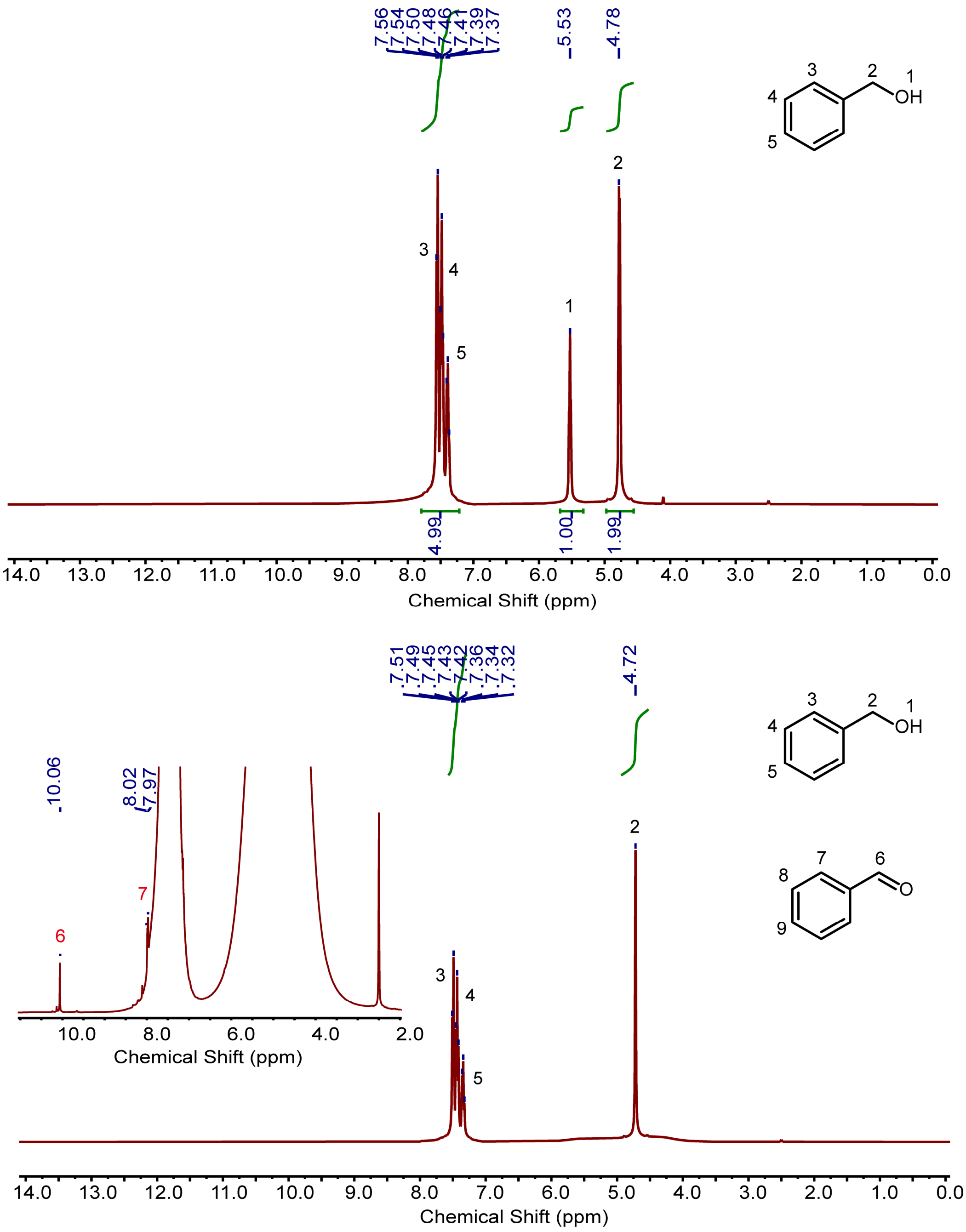

Supplementary Fig. 69 ^{1}H NMR spectra in the DMSO-d_6 solvent of the reaction system solution of COF-300-D-F before (above) and after (below) 96 h of photocatalytic H_2O_2 production (benzyl alcohol as sacrificial agent) after extraction.

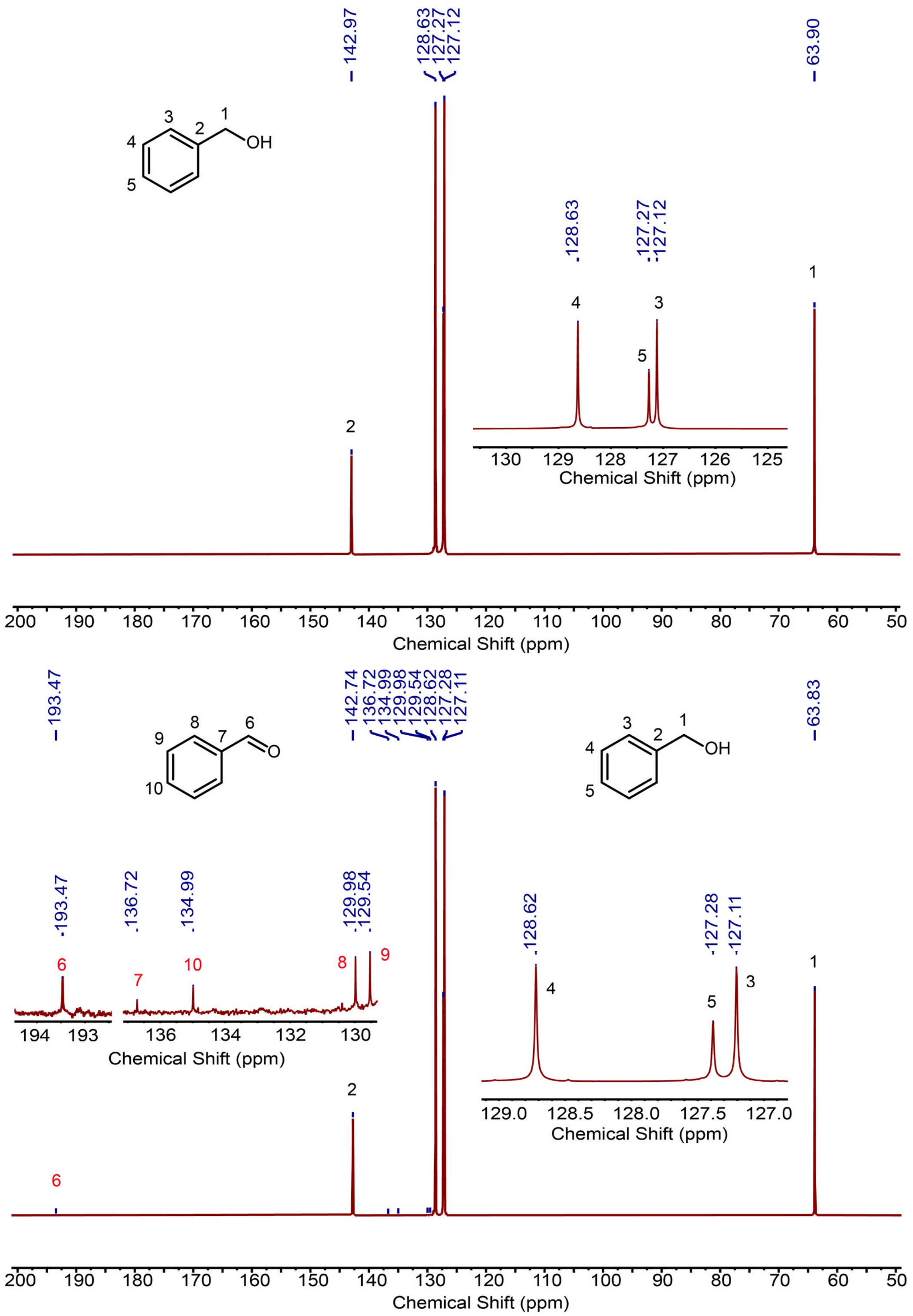

Supplementary Fig. 70 ^{13}C NMR spectra in the DMSO-d_6 solvent of the reaction system solution of COF-300-D-F before (above) and after (below) 96 h of photocatalytic H_2O_2 production (benzyl alcohol as sacrificial agent) after extraction.

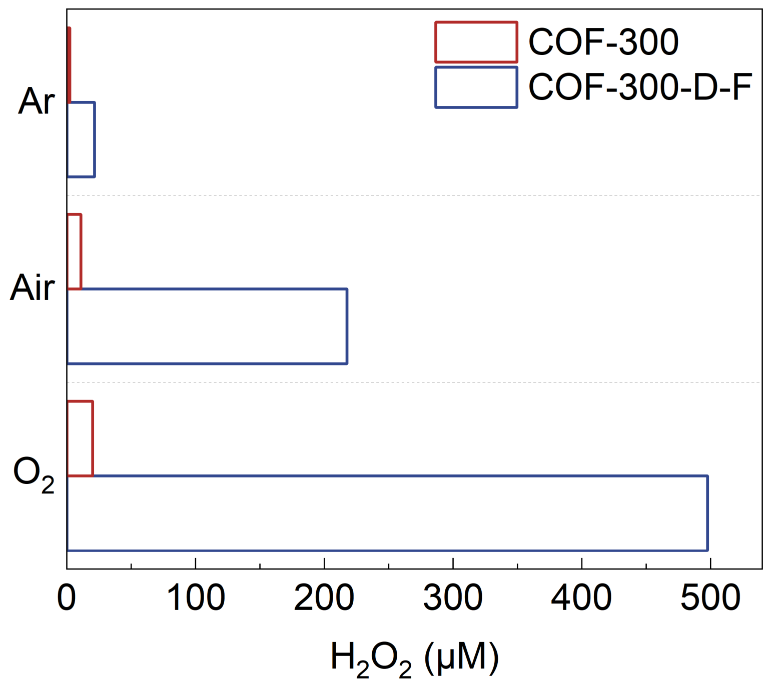

Supplementary Fig. 71 Comparison of H_2O_2 evolution catalyzed by COF-300 and COF-300-D-F in different gas atmospheres (Ar, Air, and O_2).

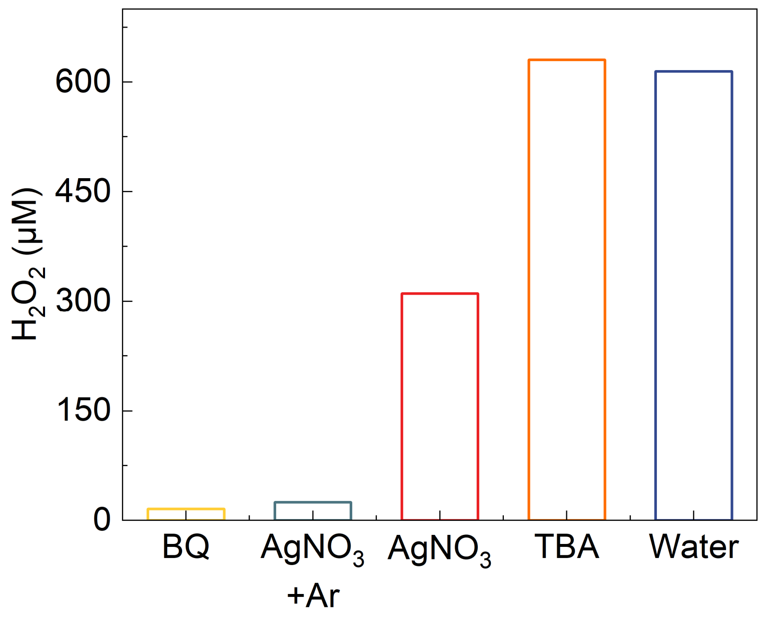

Supplementary Fig. 72 Comparison of H_2O_2 evolution catalyzed by COF-300-D-F in the presence and absence of scavengers. $AgNO_3$, tert-butyl alcohol (TBA), and benzoquinone (BQ) are electron (e^-), hydroxyl radical (·OH), and superoxide radical (·O_2^-) scavengers, respectively.

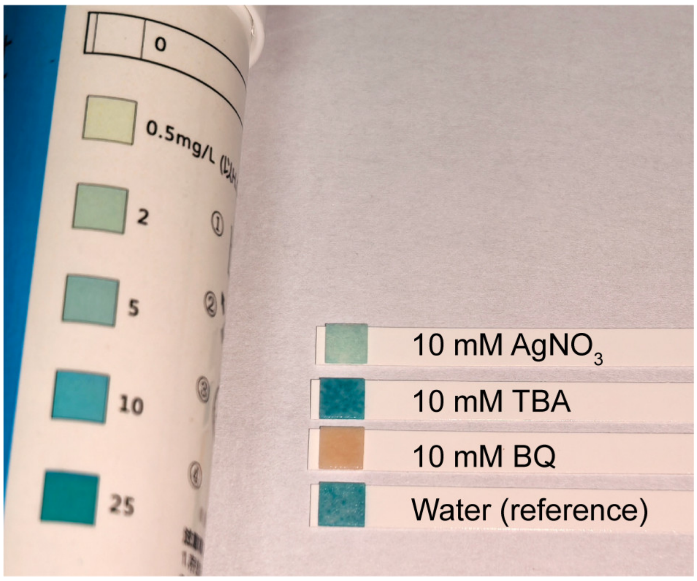

Supplementary Fig. 73 Images for photocatalytic H_2O_2 production for COF-300-D-F, with benzoquinone (BQ), tert-butyl alcohol (TBA), and $AgNO_3$ (20 mL 10 mM aqueous solution, 5 mg COF), all with 1 h illumination (Oriel Solar Simulator, 1.0 sun), as measured using Peroxide test sticks. Experiments were repeated at least three times independently with similar results.

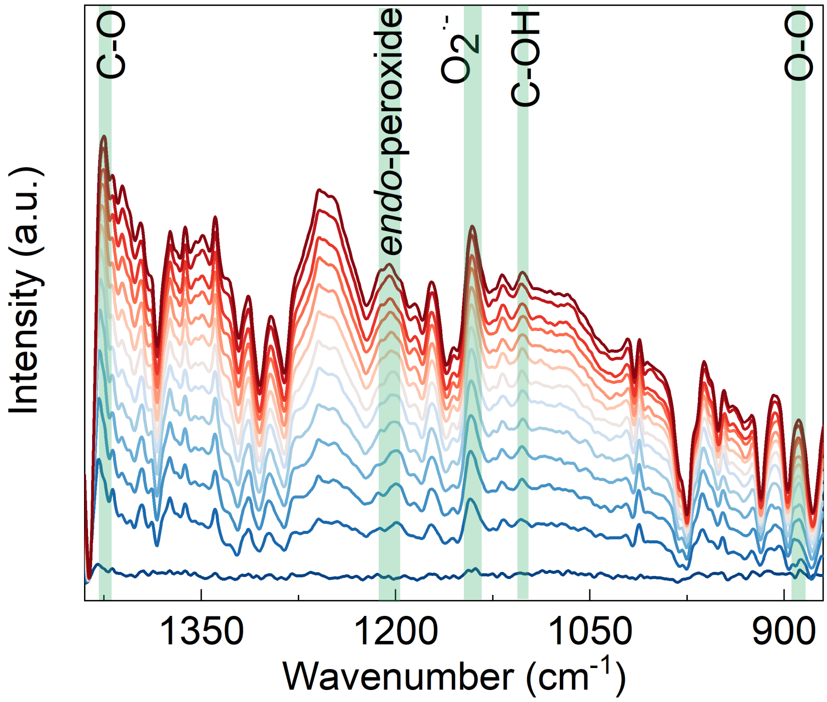

Supplementary Fig. 74 In situ diffuse reflectance infrared fourier transform spectroscopy (DRIFTS) study of COF-300-D-F collected at different photoirradiation times under O_2 and steam environment, from 1440–870 cm^{-1}.

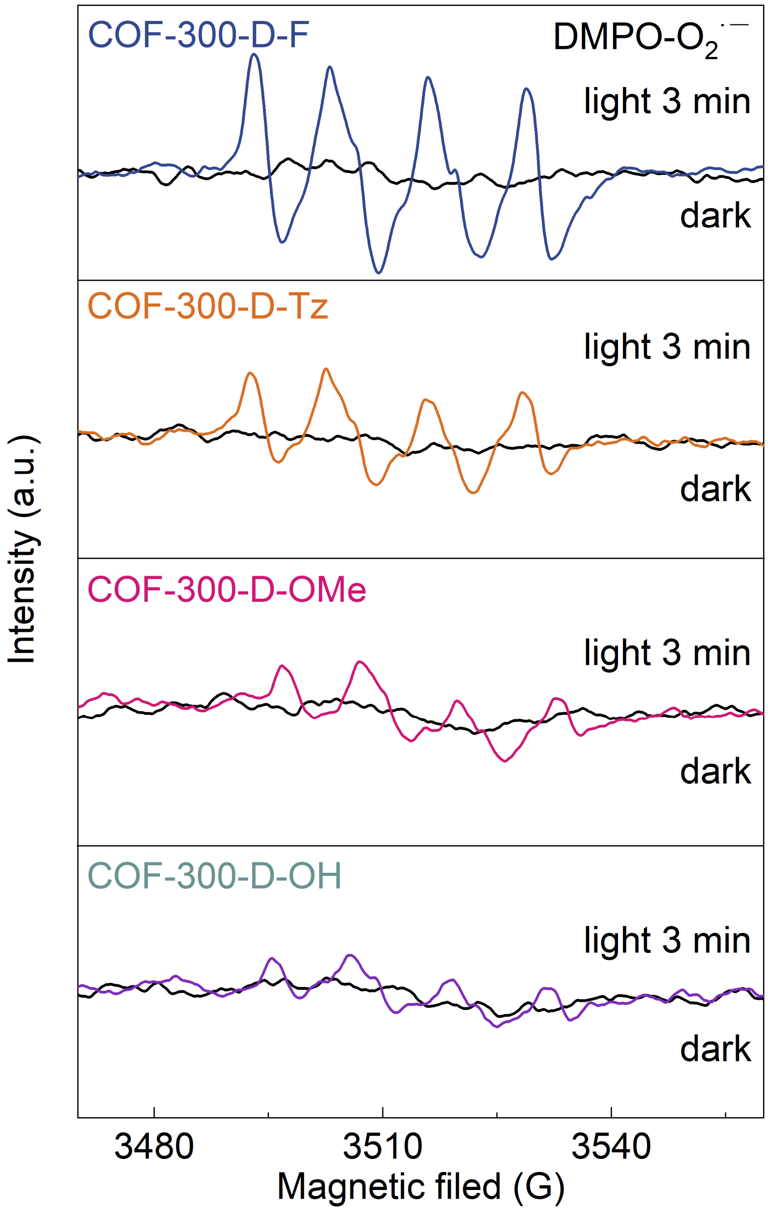

Supplementary Fig. 75 5,5-Dimethyl-1-pyrroline N-oxide (DMPO) spin trapping electron paramagnetic resonance (EPR) spectra of COF-300-D-F, COF-300-D-Tz, COF-300-D-OMe, and COF-300-D-OH for measuring $^{\bullet}O_2^-$ under dark and visible light (3 min).

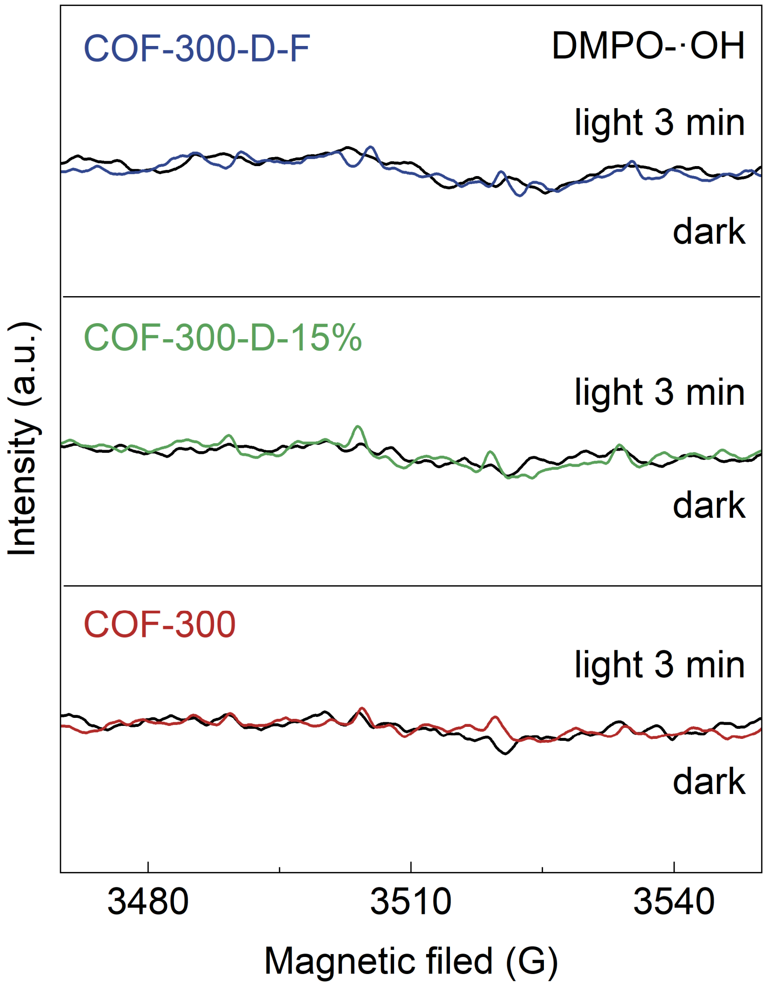

Supplementary Fig. 76 DMPO spin trapping EPR spectra of COF-300, COF-300-D, and COF-300-D-F for measuring ·OH under dark and visible light (3 min).

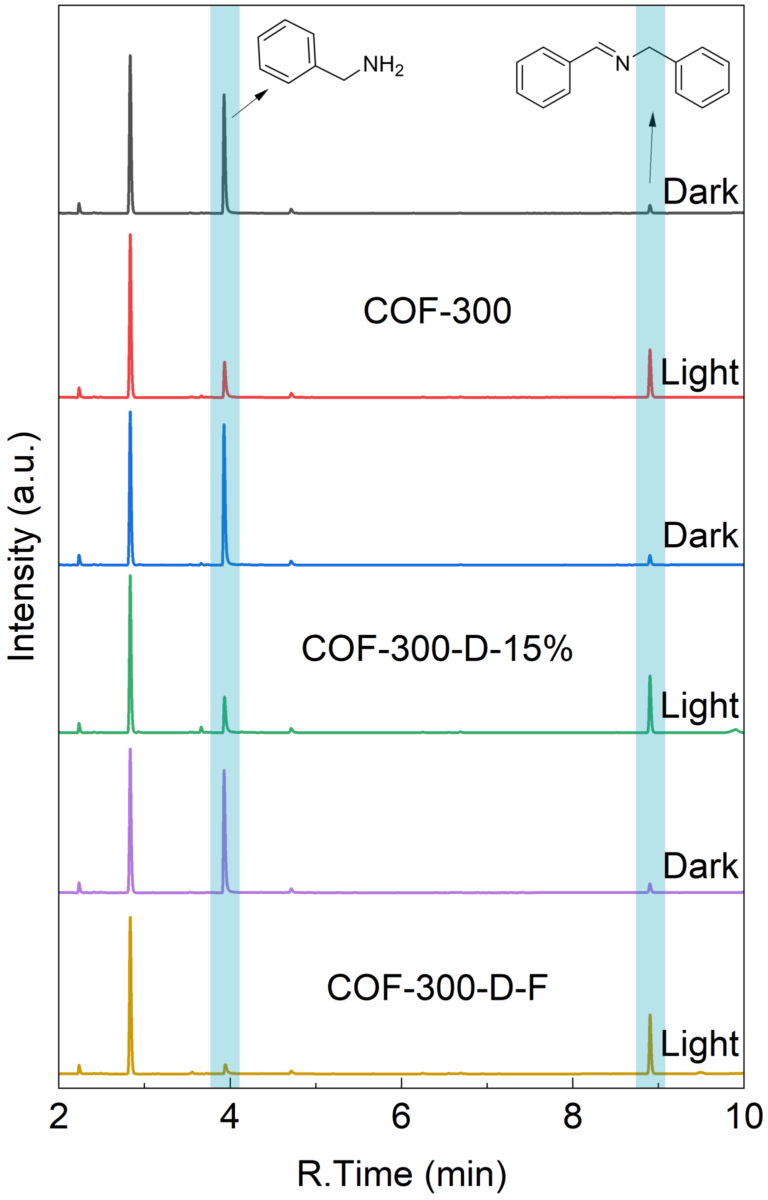

Supplementary Fig. 77 GC spectra of the reaction substrate and reaction product using benzylamine as substrate.

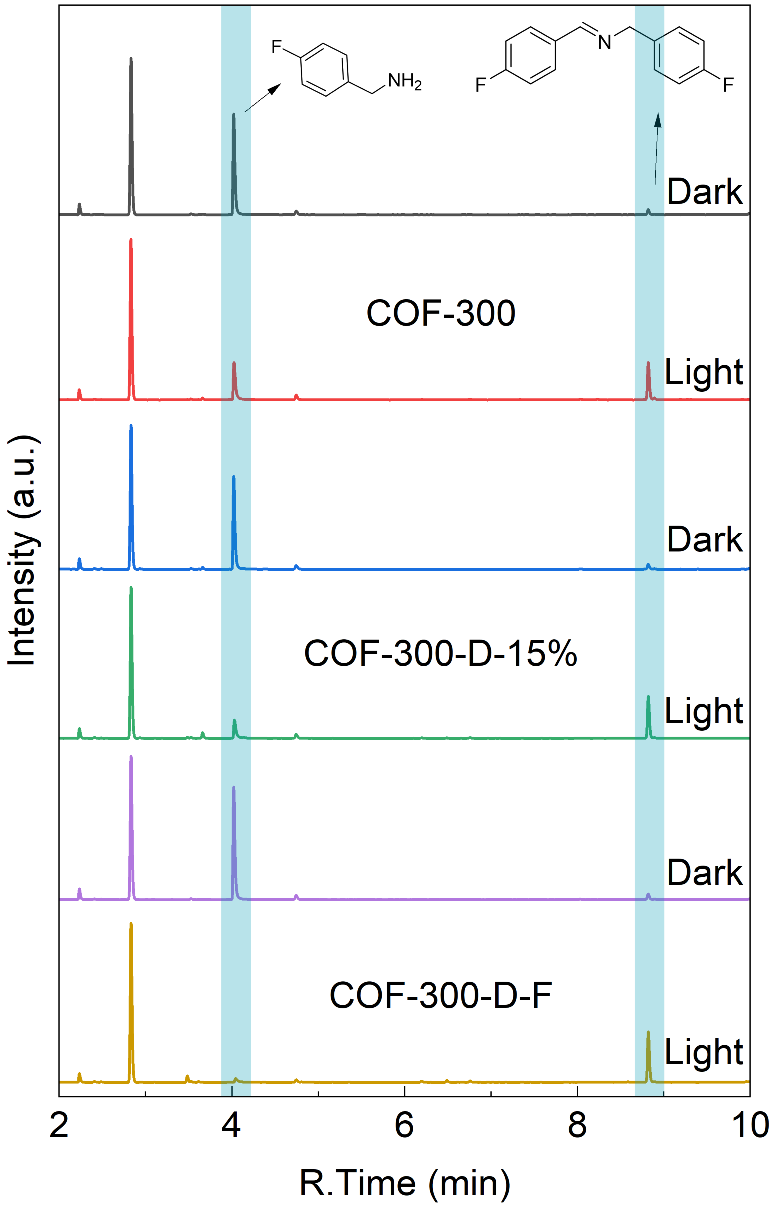

Supplementary Fig. 78 GC spectra of the reaction substrate and reaction product using 4-fluorobenzylamine as substrate.

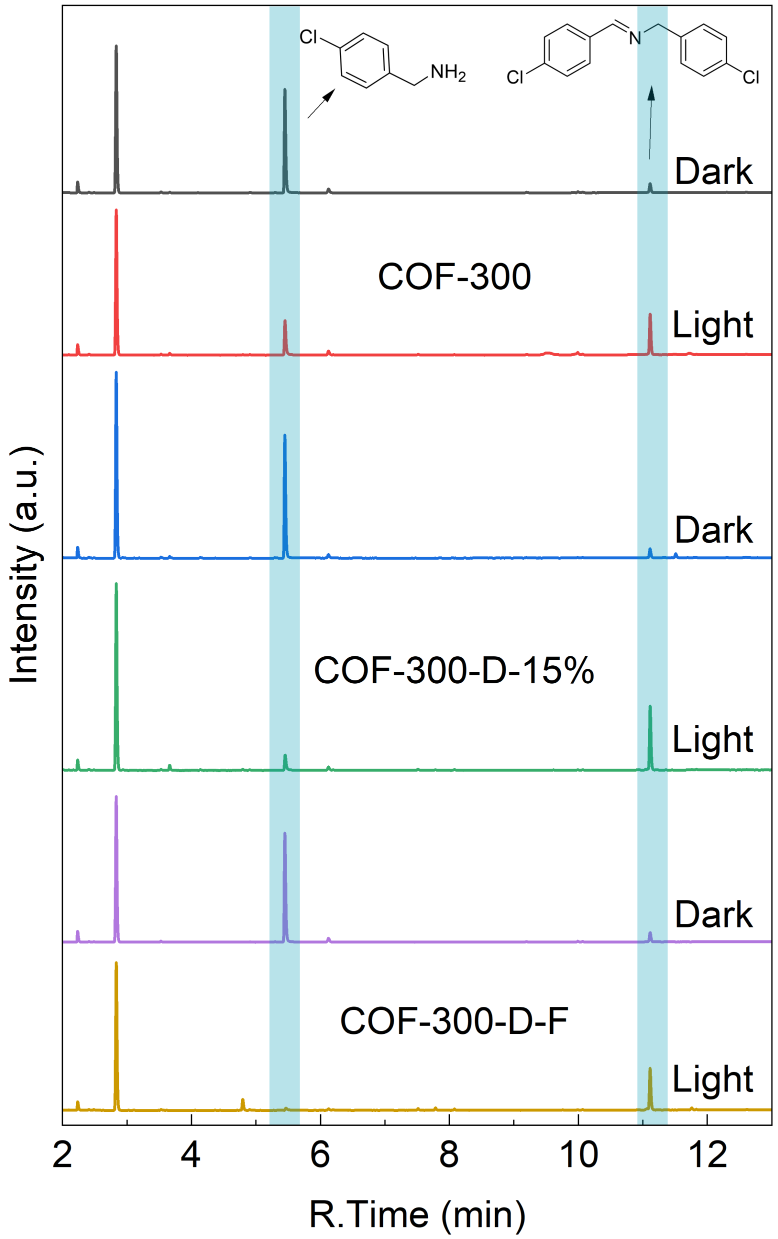

Supplementary Fig. 79 GC spectra of the reaction substrate and reaction product using 4-chlorobenzylamine as substrate.

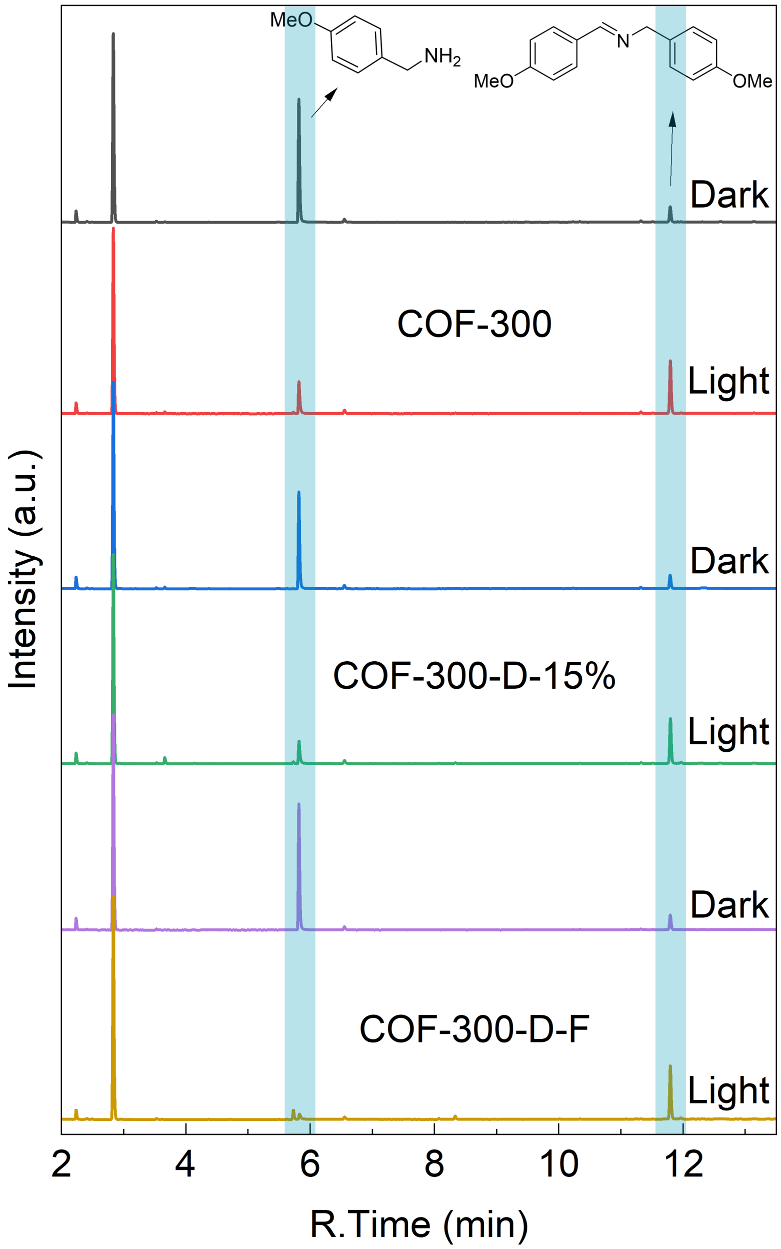

Supplementary Fig. 80 GC spectra of the reaction substrate and reaction product using 4-methoxybenzylamine as substrate.

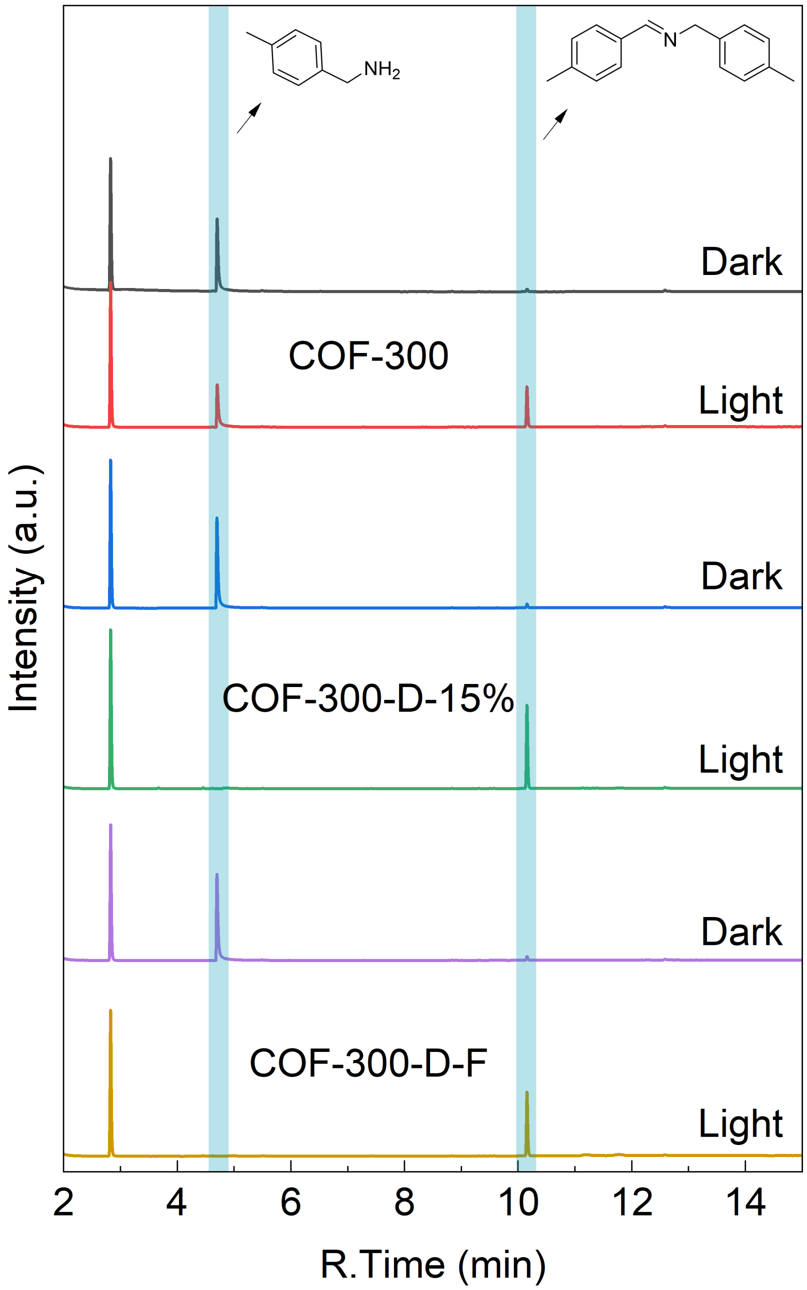

Supplementary Fig. 81 GC spectra of the reaction substrate and reaction product using 4-methylbenzylamine as substrate.

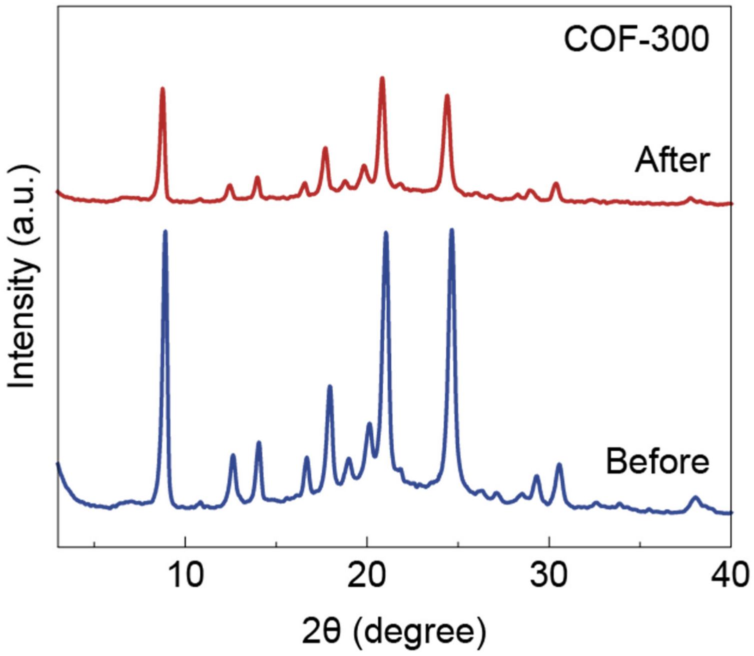

Supplementary Fig. 82 PXRD patterns of COF-300 before and after photocatalytic benzylamine coupling.

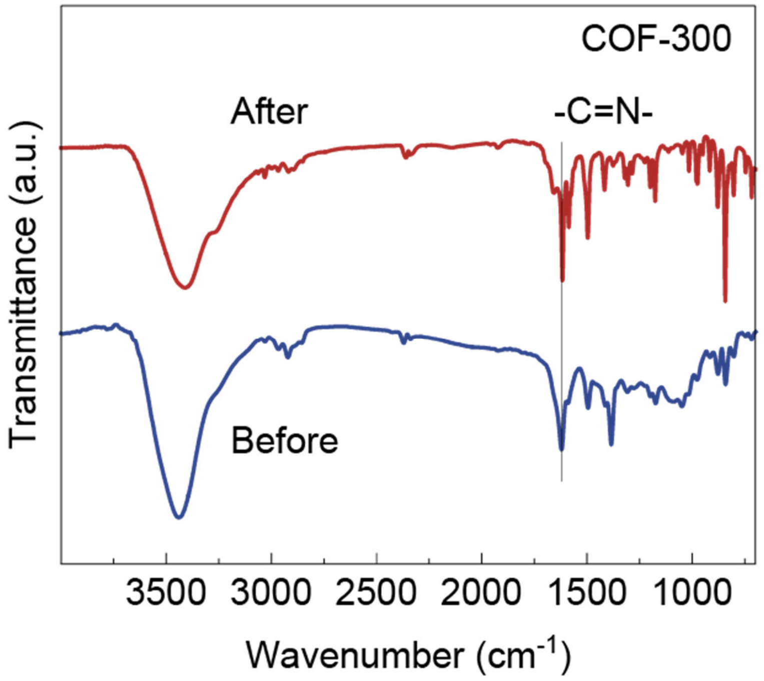

Supplementary Fig. 83 FT-IR spectra of COF-300 before and after photocatalytic benzylamine coupling.

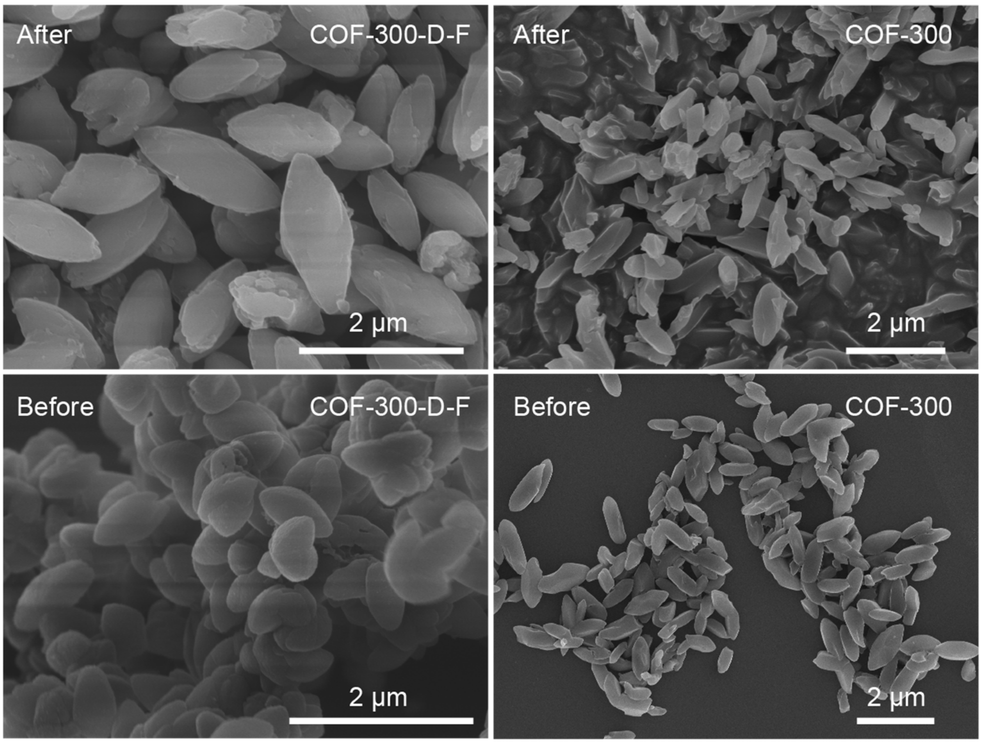

Supplementary Fig. 84 SEM images of COF-300 and COF-300-D-F before and after photocatalytic benzylamine coupling. Experiments were repeated at least three times independently with similar results.

Supplementary Table 1. Model Structures for COF-300, COF-300-D-15%, and COF-300-D-F were generated in BIOVIA Materials Studio 8.0. Simulation of PXRD patterns and Pawley Refinement were performed using the Reflex module.

	Space group	*a*	*c*	R_{wp}	R_p
COF-300	$I4_1/a$	27.23544 ± 0.00055	7.51159 ± 0.00031	2.68%	1.84%
COF-300-5%	$I4_1/a$	27.14229 ± 0.00063	7.54181 ± 0.00037	1.75%	1.26%
COF-300-10%	$I4_1/a$	27.15163 ± 0.00066	7.54749 ± 0.00039	1.59%	1.15%
COF-300-15%	$I4_1/a$	27.11790 ± 0.00067	7.55957 ± 0.00038	1.68%	1.24%
COF-300-20%	$I4_1/a$	27.10013 ± 0.00107	7.55770 ± 0.00058	1.39%	1.01%
COF-300-D-F	$I4_1/a$	27.07696 ± 0.00086	7.56828 ± 0.00047	1.54%	1.13%

Supplementary Table 2. Comparison of photocatalysts for H_2O_2 production (300 W Xe lamp; quartz reactor).

Type	Photocatalysts/Metal content (wt%)	Wavelength range / Light intensity (mW/cm^2)	Amount of catalyst (mg)/ Volume of solvent (mL)	Irradiation time (h)	H_2O_2 yields/ (μmol $g^{-1}h^{-1}$)	AQY (420 nm, %)	Solvent or sacrificial agent	Ref.
3D	TiCOF-spn /3.86	λ= 420-780 nm/ N/A	40/120	5	490	N/A	H_2O: EtOH (1:9)	1
3D	COF-NUST-16	λ ≥ 420 nm/ N/A	5/50	3	1081	N/A	H_2O: EtOH (9:1)	2
3D[a]	Au/TB-COF /0.44	λ > 420 nm/4.2	10/ N/A	5	6067	7.8	H_2O: EtOH (9:1)	3
3D[a]	NiPc-THHI-COF	λ > 400 nm/ N/A	5/50	1	4589	N/A	H_2O	4
3D	H_2Se-COF	λ > 420 nm/ N/A	5/20	4	6145	6.2	H_2O:BnOH (9:1)	5
3D	TAA-CTP-COF	full spectrum/200	10/5	1	1111	N/A	H_2O: IPA (8:2)	6
3D[b]	S-COF*	380 nm/192	5/10	1	3324	0.2	H_2O	7
3D[c]	TMB-COF-4	400-800 nm/100	10/50	4	1396	N/A	H_2O	8
2D[d]	TTB-TTA-Ph-3F	λ > 420 nm/ N/A	10/30	1	3497	13	H_2O	9
2D	PyIm-BT_F	λ > 420 nm	10/50	1	5342	2.7	H_2O: IPA (9:1)	10

2D	COF-N32	λ ≥ 420 nm/100	1/50	12	3168	6.2 (459 nm)	H_2O	11
2D	COF-2CN	λ > 420 nm/100	12.5/50	2	1601	6.8 (459 nm)	H_2O	12
2D[e]	TaptBtt	λ > 420 nm/ N/A	15/50	1.5	1407	4.6 (450 nm)	H_2O	13
Inorganic[a]	g-C_3N_4/PDI/$rGO_{0.05}$	λ ≥ 420 nm/4.33	50/30	24	23	6.1	H_2O	14
Resins[fg]	RF523	λ > 420 nm/14.03	50/30	24	51	~7.5	H_2O	15
Inorganic	OCN-500	λ ≥ 420 nm/35.2	50/50	10	106	10.2	H_2O	16
2D	CTF	λ ≥ 420 nm/44.5	30/50	24	97	N/A	H_2O	17
2D	CTF-NS-5BT	λ > 420 nm/ N/A	20/20	2	1630	6.7	H_2O:BnOH (9:1)	18
Inorganic[a]	ZnO/WO_3	300 ≤ λ ≤ 700/ N/A	50/50	1	6788	12.5 (365 nm)	H_2O:EtOH (9:1)	19
Inorganic[h]	CNIO-GaSA/1.2	λ > 420 nm/100-400	1-50/2-50	1	332	7.1 (459 nm)	H_2O	20
2D	TPE-AQ	λ ≥ 400 nm/100	10/20	1	909	N/A	H_2O	21
TAPA[e]	TpaBtt	λ > 420 nm/ N/A	15/50	1.5	252	N/A	H_2O	13
TAPA[ij]	C7	1.0 sun	3/5	1.5	200	N/A	H_2O	22

TAPA[ij]	D7	1.0 sun	3/5	1.5	22	N/A	H_2O	22
TAPA[ij]	E7	1.0 sun	3/5	1.5	44	N/A	H_2O	22
TAPA[ij]	F7	1.0 sun	3/5	1.5	67	N/A	H_2O	22
TAPA[ij]	G7	1.0 sun	3/5	1.5	44	N/A	H_2O	22
TAPA[ij]	H7	1.0 sun	3/5	1.5	89	N/A	H_2O	22
TAPA[ij]	I7	1.0 sun	3/5	1.5	22	N/A	H_2O	22
TAPA[ij]	K7	1.0 sun	3/5	1.5	67	N/A	H_2O	22
TAPA[a]	PDH-P	$\lambda > 420$ nm/ N/A	5/50	3	2195	10.5	H_2O	23
3D	COF-300-D-F	$\lambda > 420$ nm/500 $\pm$ 10	1/20	3	10086	10.6	H_2O:BnOH (9:1)	This work

EtOH = ethanol; BnOH = benzyl alcohol; IPA = isopropanol.

[a]: The type of reactor was not mentioned. [b]: Glass reaction tube. [c]: Multichannel photochemical reaction system (PCX-50C). [d]: CEL-APR100H-3 reactor. [e]: Beaker. [f]: 2 kW Xe lamp. [g]: A glass bottle sealed with a rubber stopper. [h]: Sealed round-bottomed flask. [i]: Oriel Solar Simulator, 1440 W xenon. [j]: Sample vial.

Supplementary Table 3. The amount of TAM and TAPA used for the synthesis of COF-300-D.

Samples	TAM/mg	TAPA/mg
COF-300	19.0	0.0
COF-300-D-5%	18.1	0.7
COF-300-D-10%	17.1	1.5
COF-300-D-15%	16.2	2.2
COF-300-D-20%	15.2	2.9

Supplementary Table 4. Elemental analysis of COF-300-D constructs.

Samples	N%	C%	H%
COF-300	8.41	71.63	5.29
COF-300-D-5%	8.60	74.23	5.19
COF-300-D-10%	8.92	76.08	5.29
COF-300-D-15%	9.71	78.06	5.25
COF-300-D-20%	9.74	78.71	5.24

Supplementary Table 5. The amount of different linear linkers used for the synthesis of COF-300-D-R.

Samples	BDA/mg	BDA-F/mg	BDA-Tz/mg	BDA-OMe/mg	BDA-OH/mg
COF-300-D-F	6.0	7.7	--	--	--
COF-300-D-Tz	6.0	--	8.6	--	--
COF-300-D-OMe	6.0	--	--	8.7	--
COF-300-D-OH	6.0	--	--	--	7.5

Supplementary Table 6. Abbreviation for different ligands used in the synthesis of 3D COFs in this study.

Connector 1	Connector 2	Connector 3	Abbreviation
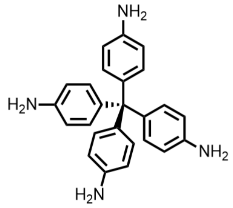			TAM
	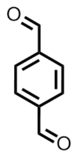		BDA
		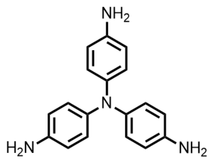	TAPA
		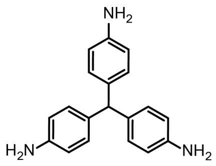	MTA

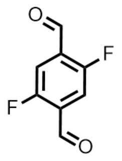

BDA-F

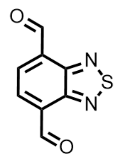

BDA-Tz

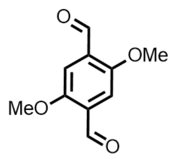

BDA-OMe

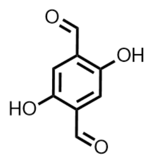

BDA-OH

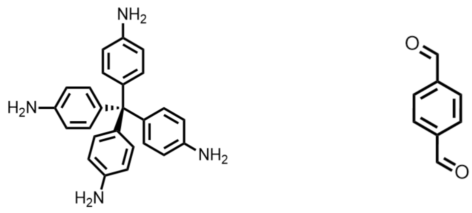

COF-300

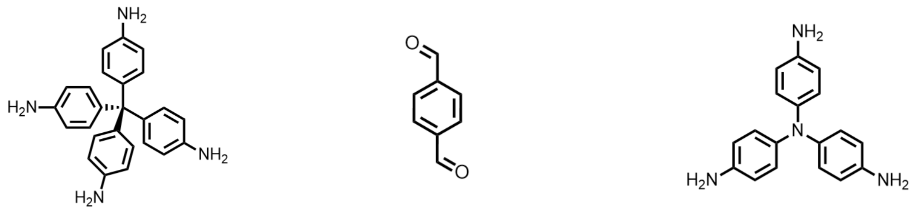

COF-300-D

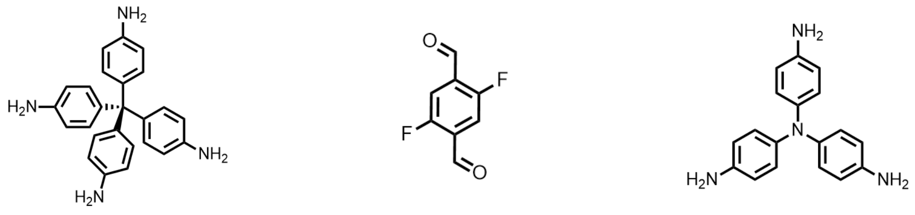

COF-300-D-F

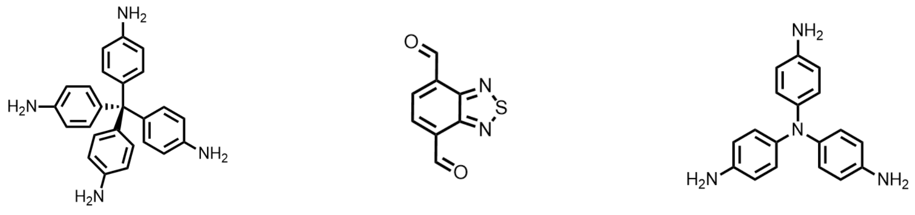

COF-300-D-Tz

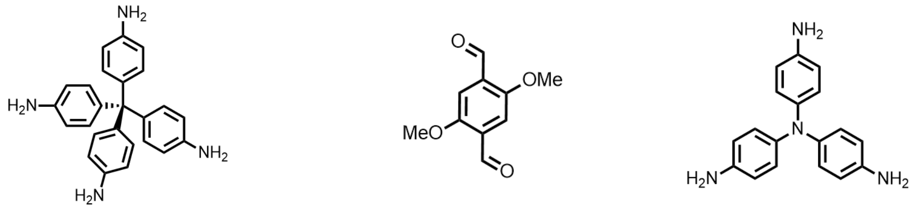

COF-300-D-OMe

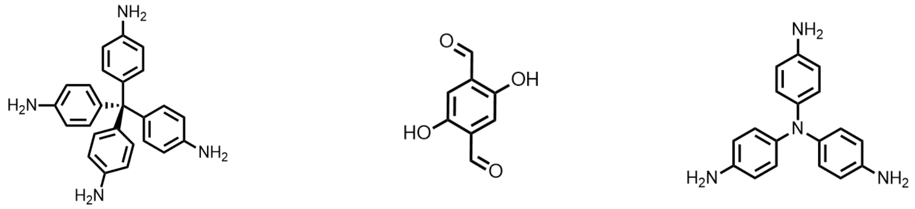

COF-300-D-OH

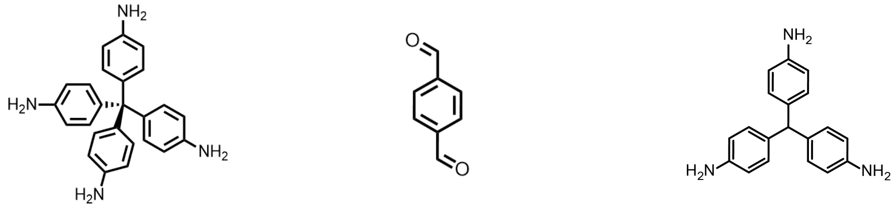

COF-300-MTA

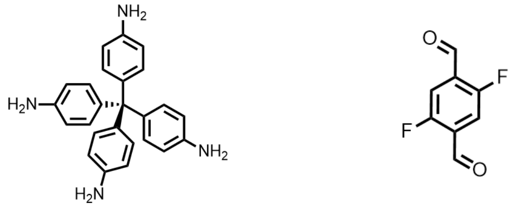

COF-300-F

Supplementary Methods

Measurement of apparent quantum yield (AQY)

For example, the calculation process of AQY at λ=400 nm is as follows:

$$AQY_{400} = \frac{(0.586\times10^{-3}\times 6.022\times10^{23}\times 6.626\times10^{-34}\times 3\times10^{8})\times2}{3.14\times4\times0.0649\times3600\times4\times10^{-7}} \times 100\% = 11.95\%$$

References：

1 Han, W.-K.*, et al.* Targeted construction of a three-dimensional metal covalent organic framework with spn topology for photocatalytic hydrogen peroxide production. *Chem. Eng. J.* **449**, 137802, (2022).

2 Wu, M.*, et al.* Three-dimensional covalent organic framework with tty topology for enhanced photocatalytic hydrogen peroxide production. *Chem. Eng. J.* **454**, 140121, (2023).

3 Zhang, Y.*, et al.* Hollow spherical covalent organic framework supported gold nanoparticles for photocatalytic H2O2 production. *Chinese Journal of Catalysis* **57**, 143-153, (2024).

4 Wang, X., *et al.* 12 Connecting Sites Linked Three-dimensional Covalent Organic Frameworks with Intrinsic Non-interpenetrated shp Topology for Photocatalytic H2O2 Synthesis. *Angew. Chem. Int. Ed.* **63**, e202401014, (2024).

5 Dong, P., *et al.* Stepwise Protonation of Three-Dimensional Covalent Organic Frameworks for Enhancing Hydrogen Peroxide Photosynthesis. *Angew. Chem. Int. Ed.* **63**, e202405313, (2024).

6 Liao, J.-P., *et al.* Flexible Units Induced Three-Dimensional Covalent Organic Frameworks with a Heteromotif Molecular Junction for Photocatalytic H2O2 Production. *ACS Catal.* **14**, 3778-3787, (2024).

7 Ma, T.-T., *et al.* Photochromic radical states in 3D covalent organic frameworks with zyg topology for enhanced photocatalysis. *National Science Review* **11**, nwae177, (2024).

8 Zhu, R.-M., *et al.* Three-Dimensional Covalent Organic Frameworks Based on Linear and Trigonal Linkers for High-Performance H2O2 Photosynthesis. *Angew. Chem. Int. Ed.* **64**, e202412890, (2025).

9 Rong, Q., *et al.* Modulating Quinoline-Linked Covalent Organic Frameworks via Fluorination for Boosting the Photocatalytic Air Reductive H2O2 Production. *ACS Sustainable Chem. Eng.* **12**, 13306-13315, (2024).

10 Li, Z., *et al.* Pyridyl-Imine-Functionalized Donor–Acceptor Covalent Organic Frameworks for Optimal Photosynthesis of Hydrogen Peroxide. *Adv. Energy Mater.* **n/a**, 2500341, (2025).

11 Liu, F., *et al.* Covalent organic frameworks for direct photosynthesis of hydrogen peroxide from water, air and sunlight. *Nat. Commun.* **14**, 4344, (2023).

12 Hou, Y., *et al.* Efficient Photosynthesis of Hydrogen Peroxide by Cyano-Containing Covalent Organic Frameworks from Water, Air and Sunlight. *Angew. Chem. Int. Ed.* **63**, e202318562, (2024).

13 Qin, C., *et al.* Dual donor-acceptor covalent organic frameworks for hydrogen peroxide photosynthesis. *Nat. Commun.* **14**, 5238, (2023).

14 Kofuji, Y., *et al.* Carbon Nitride–Aromatic Diimide–Graphene Nanohybrids: Metal-Free Photocatalysts for Solar-to-Hydrogen Peroxide Energy Conversion with 0.2% Efficiency. *J. Am. Chem. Soc.* **138**, 10019-10025, (2016).

15 Shiraishi, Y., *et al.* Resorcinol–formaldehyde resins as metal-free semiconductor photocatalysts for solar-to-hydrogen peroxide energy conversion. *Nat. Mater.* **18**, 985-993, (2019).

16 Wei, Z., *et al.* Efficient visible-light-driven selective oxygen reduction to hydrogen peroxide by oxygen-enriched graphitic carbon nitride polymers. *Energy Environ. Sci.* **11**, 2581-2589, (2018).

17 Chen, L., *et al.* Acetylene and Diacetylene Functionalized Covalent Triazine Frameworks as Metal-Free Photocatalysts for Hydrogen Peroxide Production: A New Two-Electron Water Oxidation Pathway. *Adv. Mater.* **32**, 1904433, (2020).

18 Yu, X., *et al.* Electronic Tuning of Covalent Triazine Framework Nanoshells for Highly Efficient Photocatalytic H2O2 Production. *Adv. Sustainable Syst.* **5**, 2100184, (2021).

19 Jiang, Z., *et al.* S-scheme ZnO/WO3 heterojunction photocatalyst for efficient H2O2 production. *Journal of Materials Science & Technology* **124**, 193-201, (2022).

20 Tan, H., *et al.* Photocatalysis of water into hydrogen peroxide over an atomic Ga-N5 site. *Nat. Synth.* **2**, 557-563, (2023).

21 Ye, Y.-X., *et al.* A solar-to-chemical conversion efficiency up to 0.26% achieved in ambient conditions. *PNAS* **118**, e2115666118, (2021).

22 Zhao, W., *et al.* Accelerated Synthesis and Discovery of Covalent Organic Framework Photocatalysts for Hydrogen Peroxide Production. *J. Am. Chem. Soc.* **144**, 9902-9909, (2022).

23 Lei, Y., *et al.* A High-Throughput Preparation Strategy for Microporous Polyimides and Their Photocatalysis Performance: Understanding Polymerization Kinetics and Thermodynamics in a Solvothermal System. *Macromolecules* **57**, 745-755, (2024).
