# Supplementary material for: Defective three-dimensional covalent organic frameworks for enhanced hydrogen peroxide photosynthesis and organic transformation
Source: Nat Commun. 2026 Mar 27;17:4505. doi: 10.1038/s41467-026-71137-0 (PMC13187191; doi:10.1038/s41467-026-71137-0)
Supplement: Supplementary file 2 — Description of Additional Supplementary File [file 41467_2026_71137_MOESM2_ESM.pdf]

Description of Additional Supplementary File

Supplementary Data 1. The atomic coordinates of the optimized computational models of COF-300, COF-300-D, and COF-300-D-F.
